# Expansion and stress responses of AP2/EREBP superfamily in *Brachypodium Distachyon*

**DOI:** 10.1038/srep21623

**Published:** 2016-02-12

**Authors:** Lihong Chen, Jiapeng Han, Xiaomin Deng, Shenglong Tan, Lili Li, Lun Li, Junfei Zhou, Hai Peng, Guangxiao Yang, Guangyuan He, Weixiong Zhang

**Affiliations:** 1 The Institute for Systems Biology, Jianghan University, Wuhan 430056, China; 2 The Genetic Engineering International Cooperation Base of Chinese Ministry of Science and Technology, Chinese National Center of Plant Gene Research (Wuhan) HUST Part, College of Life Science and Technology, Huazhong University of Science & Technology (HUST), Wuhan 430074, China; 3 Ministry of Agriculture Key Laboratory of Biology and Genetic Resources of Rubber Tree/State Key Laboratory Breeding Base of Cultivation and Physiology for Tropical Crops, Rubber Research Institute, Chinese Academy of Tropical Agricultural Sciences, Danzhou 571737, China; 4 School of Information Engineering, Hubei University of Economics, Wuhan 430205, China; 5 Department of Computer Science and Engineering and Department of Genetics, Washington University, St. Louis, MO 36130, USA

## Abstract

APETALA2/ethylene-responsive element binding protein (AP2/EREBP) transcription factors constitute one of the largest and most conserved gene families in plant, and play essential roles in growth, development and stress response. Except a few members, the AP2/EREBP family has not been characterized in *Brachypodium distachyon*, a model plant of Poaceae. We performed a genome-wide study of this family in *B. distachyon* by phylogenetic analyses, transactivation assays and transcript profiling. A total of 149 AP2/EREBP genes were identified and divided into four subfamilies, i.e., ERF (ethylene responsive factor), DREB (dehydration responsive element binding gene), RAV (related to ABI3/VP) and AP2. Tandem duplication was a major force in expanding *B. distachyon* AP2/EREBP (BdAP2/EREBP) family. Despite a significant expansion, genomic organizations of BdAP2/EREBPs were monotonous as the majority of them, except those of AP2 subfamily, had no intron. An analysis of transcription activities of several closely related and duplicated BdDREB genes showed their functional divergence and redundancy in evolution. The expression of BdAP2/EREBPs in different tissues and the expression of DREB/ERF subfamilies in *B. distachyon*, wheat and rice under abiotic stresses were investigated by next-generation sequencing and microarray profiling. Our results are valuable for further function analysis of stress tolerant AP2/EREBP genes in *B. distachyon*.

The APETALA2/ethylene-responsive element binding protein (AP2/EREBP) superfamily is one of the largest groups of plant specific transcription factors. They play vital roles in plant growth, development and response to such diverse stresses as extreme temperature (freezing damage, and high temperature stress), drought, high salinity and pathogen infection^1^,^2^,^3^,^4^,^5^,^6^,^7^. They have also been implicated in various hormones-related signal transduction pathway including abscisic acid (ABA), ethylene, cytokinin and jasmonates (JAs)^8^,^9^,^10^,^11^,^12^.

Many AP2/EREBP genes have been identified in diverse plant species, such as *Arabidopsis* (145)^13^, poplar (200)^14^, soybean (98)^15^, grape (149)^16^, tomato (85)^17^, rice (163)^7^, potato (155)^18^ and foxtail millet (171)^19^. These genes have been studied in domain conservation, features of amino acid sequence and biological functions. The AP2/EREBP genes share a highly conserved AP2 DNA binding domain with 50 to 70 amino acid residues, which consist of a three-stranded anti-parallel β-sheet and an α-helix^20^. This AP2 domain was first discovered in the amino acid sequence of a homeotic gene *APETALA2* (*AP2*) when studying *Arabidopsis* flower and seed development^21^. The AP2/EREBP genes are divided into three families based on the number of AP2 domain and sequence similarity, i.e., the AP2 family, the RAV (related to ABI3/VP1) family and the ERF (ethylene response factor) family. The AP2 family, containing two AP2 domains and essential for plant development^22^,^23^, is further categorized into the AP2 and ANT (AINTEGUMENTA) groups in seed plants. The RAV family, consisting of a AP2 domain and a B3 DNA binding domain, functions as negative regulators in plant development and is important for mediating abiotic and biotic stress responses^24^,^25^,^26^,^27^. The ERF family, hosting a single AP2 domain and being the largest in the AP2/EREBP superfamily, has been revealed to be critical regulators of diverse processes in plant. Based on the amino acid sequence of the AP2 domain, the ERF family can be further divided into the dehydration-responsive element-binding protein (DREB) and ERF subfamilies, or the groups A and B, each of which consists of six groups individually. Nevertheless, based on a phylogeny, the ERF family has been divided into 12 and 15 groups in *Arabidopsis* and rice, which is different from DREB and ERF, two major subfamilies used before^28^.

Much effort has been devoted to elucidation of the functions of the DREB and ERF subfamily. The DREB genes activate multiple stress-responsive genes by interacting with the C-repeat/dehydration responsive element (CRT/DRE), which has a core motif of A/GCCGAC^29^,^30^, in the promoters of target genes. They regulate stress-responsive genes under various abiotic stresses, including low temperature^31^,^32^,^33^, heat^34^,^35^, drought^5^,^6^,^36^,^37^ and high salinity^36^. Thus they are excellent candidates for genetic breeding to enhance crop stress tolerance. The genes of the ERF subfamily, which play more diverse functions than the genes of the DREB subfamily, have been shown to induce or repress gene expression in response to external stimuli of ethylene, cytokinin and abiotic stresses, such as wounding, cold, high salinity, and drought^9^,^38^,^39^,^40^ by recognizing the GCC-box^20^.

Coupled with a small genome, short life cycle, and easy cultivation and transformation, *B. distachyon*, becomes an excellent model plant for functional genomics of temperate cereals and forage grasses^41^ and for developing sustainable energy and feed stocks^42^,^43^. With the genome of the diploid inbred line Bd21 being sequenced^44^,^45^,^46^, several gene families of *B. distachyon*, including *WRKY*^47^,^48^,^49^, *MAPK* and *MAPKK*^50^,^51^, *bZIP*^52^, and *NAC*^53^, have been studied. However, the AP2/EREBP superfamily remains largely unexplored in *B. distachyon*, although the C-repeat binding factors (CBFs or DREBs) have been identified and investigated^54^,^55^ in *B. distachyon* and other monocotyledon plants^56^,^57^,^58^,^59^,^60^,^61^. Genomic comparison, an effective means to transferring knowledge of one taxon to another^62^, could be adopted to understand the expansion and functions of this superfamily of genes among *Brachypodium*, rice, wheat, and sorghum after divergence during evolution.

In this study, we systematically investigated the AP2/EREBP superfamily in *B. distachyon* by adopting and combining comparative and phylogenetic analyses, transactivation assay, and gene expression profiling. We report here our findings on expansion and conservation of this large and critical set of transcription factors in *B. distachyon* and their functions as gene regulators in response to abiotic stresses.

## Results and Discussion

### Identification of the AP2/EREBP superfamily in *B. distachyon*

Based on a stringent HMM and using restrictive BLAST searches (see Methods), 189 putative AP2/EREBP proteins were identified in the *B. distachyon* reference genome (version 3.0) and represent 0.44% of the annotated proteins in *B. distachyon*^19^,^44^. This ratio is much closer to that of *Setaria italica* (0.44%)^19^ and rice^7^ (0.43%), but less than that of *Arabidopsis* (0.55%)^19^,^28^,^63^. Among the 189 putative AP2/EREBP proteins, 13 were splicing variants and thus discarded from further consideration, and 17 additional proteins (Table S1) were also excluded from further analysis because their AP2 domains were too short or divergent to fit into an acceptable phylogeny. The remaining 149 putative BdAP2/EREBP genes (Table S1) were used for further analysis.

Based on sequence similarities, the composition of domains, and the number of the AP2 domain, the 149 BdAP2/EREBP genes could be divided into three subfamilies. Specifically, 23 genes encoded two complete AP2 domains and were thus assigned to the AP2 subfamily; 4 genes had a single AP2 domain and a single B3 domain and were classified into the RAV subfamily; and the remaining 122 genes carried a single AP2/ERF domain and were assigned to the DREB/ERF subfamilies. The DREB/ERF subfamily members were further divided into DREB and ERF subfamilies based on the phylogeny in the following analysis. Since no standard nomenclature has been adopted for the AP2/EREBP family genes, each subfamily gene was named with a two-letter code corresponding to *B. distachyon* (Bd), followed by a subfamily designation (AP2, RAV, DREB or ERF), and a number based on the order of its chromosomal locations. This scheme followed the naming convention suggested for the AP2/EREBP family genes in *Setaria italic*^19^. Table S1 lists the detailed information of these *B. distachyon* AP2/EREBP genes, including accession numbers, number of amino acids, and expression sequence tag (ESTs).

### Phylogeny of the AP2/EREBP genes in *B. distachyon*

**Figure 1:**
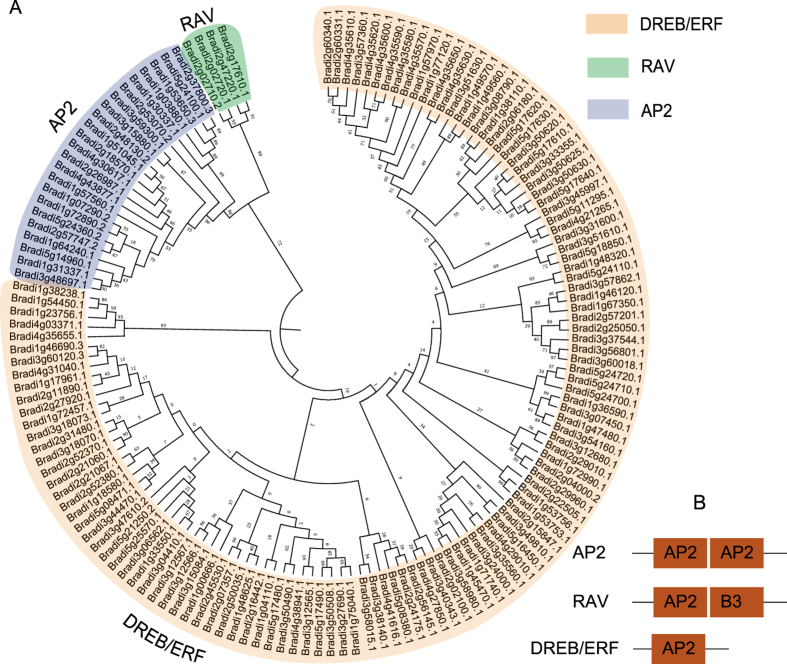
(**A**) Phylogeny of the *B. distachyon* AP2/EREBP (APETALA2/ethylene-responsive element binding protein) family. (**B**) Domain structures of the AP2/EREBP genes.

In order to appreciate the phylogenetic relationship of the *BdAP2/EREBP* genes, a phylogenetic tree was constructed using the 149 AP2/EREBP protein sequences. The 149 genes clustered into distinct RAV, AP2 and DREB/ERF clades comprising of 4, 23 and 122 proteins, respectively (Fig. 1). The result was consistent with the above classification result based on domain compositions and the number of AP2 domains. The number of *B. distachyon* RAV subfamily genes was nearly the same as that in *Setaria italica*^1^, *rice*^7^, *Arabidopsis*^13^, *Populus trichocarpa*^14^, *Vitis vinifera*^16^ and *Salix arbutifolia*^64^, showing a strong conservation of this subfamily and suggesting these RAV genes in all of these plants may share a common ancestor prior to the separation of *Brachypodium* from the other plants. However, the number of DREB/ERF subfamily members varied significantly in these plants, indicating that they might have independently undergone different expansions in evolution.

**Figure 2:**
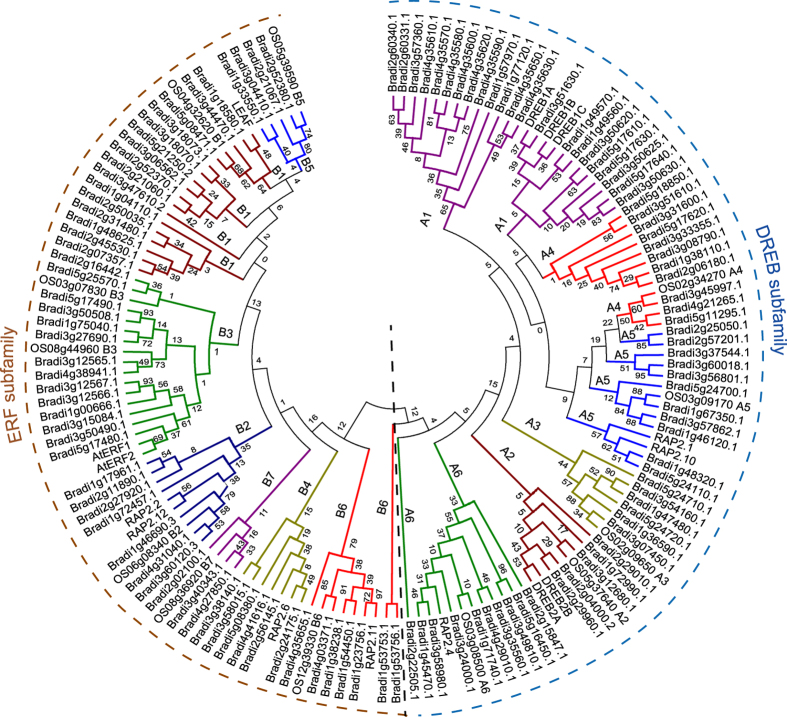
Phylogeny of the *B. distachyon* DREB (dehydration responsive element binding gene) and ERF (ethylene responsive factor) subfamily proteins based on their conserved domain sequences. Different subgroups of DREB and ERF subfamily proteins are highlighted in different colors.

The AP2 domain sequences of the 122 ERF/DREBs and 25 published ERF/DREBs^7^,^28^ were used to infer the phylogenetic history of the ERF/DREB genes. As shown in the phylogenic tree in Fig. 2, the 122 BdERF/DREBs were further classified into two subfamilies based on the amino acid sequences of the AP2 domains: the DREB subfamily has 65 (53% of the 122) genes encoding DREB/CBF-like proteins and the ERF subfamily contains 57 (47%) genes encoding ERF-like proteins. Moreover, the DREB subfamily was further divided into six groups as in *Arabidopsis*^28^, among which the A1 group was the largest and the A2 group the smallest (Fig. 2). Likewise, the ERF subfamily was classified into seven groups as in rice^7^, with the B1 subgroup being the largest (Fig. 2). Interestingly, the ratio of the DREB subfamily members in the 122 BdERF/DREBs is higher than that of the ERF subfamily (Table S1). This case indicated each subfamily of AP2/EREBPs evolved with species-specific characteristics.

The DREB subfamily members (Fig. 2) included or overlapped with the BdCBF members reported in previous study^54^,^55^. In particular, all the 19 DREB/CBFs identified by Ryu *et al*.^55^ fell into the A1 subgroup (Table S2), while six members (Bradi1g49560, Bradi1g49570, Bradi3g50630, Bradi5g17610, Bradi5g17630 and Bradi5g17640) were missed in the study by Li *et al*.^54^. Among the 18 DREB/CBFs reported in Li *et al*.^54^, 14 fell into A1 subgroup (Table S2); Bradi4g35640 which was not identified in our study nor in Ryu *et al*.^55^, had no obvious AP2 domain, two other CBFs, Bradi3g45997 and Bradi4g21265 in the A4 subgroup (Table S2), were missed by Ryu *et al*.^55^; and Bradi3g45997 was counted twice. These results suggested that our work was rigorous for identification of AP2/EREBP genes in *B. distachyon*.

### Conservation and divergence of DREB/ERF subfamily genes during monocot evolution

DREB/ERF subfamily genes are important regulators of stress response in plants. The size of this subfamily varies greatly across different species relative to the AP2 and RAV subfamilies (Table S3). To understand the expansion and variation of DREB/ERF subfamily in Poaceae, the phylogenetic tree of each group of the DREB/ERF subfamily in *B. distachyon* and several other plants of Poaceae was constructed based on their conserved AP2 domains. The Poaceae evolved into several subfamilies 55–70 million years ago (MYA)^57^. Compared to members of the Pooideae including the temperate cereals wheat, barley and oat, the subfamilies of Panicoideae (maize, sorghum) and Oryzaceae (rice) are preferentially distributed in tropical regions^57^. The AP2/EREBP superfamily in rice^7^, sorghum^59^ and wheat^60^ has been identified recently. Therefore, the three species were used as representatives of the Oryzaceae, Panicoideae and Pooideae subfamilies, respectively, for phylogenic analysis of the DREB/ERF subfamilies.

The genes with substantially divergent sequences were discarded in constructing the phylogenic tree of each group of DREB/ERF subfamilies. As shown in Figure S1, the A1 group (CBF) of DREB subfamily could be classified into four subgroups (I, II, III and IV) as similarly done for wheat^57^. Subgroup I contains only one gene (*Ta.47061*, also named as *CBFIa-A11*) in wheat, two in rice, and four in *B. distachyon*, but none in sorghum, suggesting that CBFIa homologs in *B. distachyon* and rice may have expanded following divergence from wheat. The second subgroup (CBFII) contains three genes in wheat (Pooideae), one each in sorghum (Panicoideae) and *B. distachyon* (Pooideae), but none in rice (Oryzaceae). Such differences indicated that the subgroup II CBF genes of rice may have separated earlier from other subfamilies of the Poaceae. Within the four subgroups of the DREB A1 group, the third subgroup is the largest and can be further organized in four distinct branches of the phylogenic tree, named as IIIa, IIIb, IIIc and IIId following an early report^57^. Group IIIa is the only one containing CBF homologs from the Oryzaceae, Panicoideae and Pooideae subfamilies. This result indicated that the CBFIIIa genes were already present before the separation of these subfamilies. Groups IIIb and IIIc were grouped together in the phylogenetic tree (Figure S1) and contained CBF homologs exclusively from the two subfamilies of Oryzaceae and Pooideae. Interestingly, group IIId contained CBF genes only from *B. distachyon* (11 BdCBFs, 7 were originated from gene duplication) and wheat (6 TaCBFs, 4 were originated from gene duplication) of Pooideae subfamily and the CBF genes from each of the two species clustered together, respectively. This result revealed an obvious monophyletic origin of the CBFIIId genes and indicated that this group evolved following the appearance of the Pooideae. Compared with subgroup III, the CBF subgroup IV (CBFIV) had fewer members. It formed two clades; the big one included CBF genes exclusively from wheat of Pooideae subfamily, and the small clade contained CBF genes from rice of Oryzaceae and *B. distachyon* of Pooideae subfamilies. However, none of the CBFIV genes was found in the Panicoideae subfamily, as similarly described in wheat^57^. This result indicated that these CBFIV genes may evolve to serve some special function in the three species of wheat, rice and *B. distachyon* and evolve much faster in wheat than in other two species after their separation. For some other CBF genes, such as *Os09g35010,* that do not cluster together with any of the above described subgroups (Figure S1), we did not force to classify them, since they may have evolved with species specific characteristics or they may be more adequately grouped with some genes in other subfamilies of Poaceae to better reflect their evolutionary relationships. The other DREB/ERF subgroups were processed similarly. In general, the CBF group has more genes in wheat and *B. distachyon* than in the two other Poaceae species, and its subgroups IIId and IV have expanded significantly (Figure S1), which might contributed to their specific function against stresses such as cold.

Beyond wheat and *B. distachyon*, the DREB family seemed to evolve differently in the two other Poaceae plants we examined. DREBs in sorghum expanded mainly in the subgroup II of the A2 group and the subgroup IV of the A5 group, which formed an independent branch in the phylogenetic tree (Figure S2). In comparison, DREBs in rice expanded primarily in the subgroup II of the A4 group and the subgroup I of the A5 group (Figure S2). The A3 and A4 group do not contain DREBs in wheat, while only two subgroups (subgroup III of A1 and subgroup I of A6) contain the DREBs in the four species we studied. These results showed the specificity and conservation of DREB subfamily genes after the separation of the four species.

Similar analysis was conducted on each subgroup of ERF subfamily. As shown in Figure S3, B1 group had the largest number, followed by B2 and B3 groups. Each group of ERF subfamily genes could form several hybrid or monophyletic clades. For example, some ERF genes from rice (subgroup II of B1) or sorghum (subgroup VI of B1 and subgroup IV of B4) developed into an independent branch as observed in the DREB genes. All the subgroups of B2 group contain the ERF homologs from the four species of wheat, *B. distachyon*, rice and sorghum, while the DREB subgroups have only two like this, which showed that these genes may have a common ancestor prior to the separation of the four species. The genes forming a monophyletic clade may evolve fast and specifically into their own subfamily.

In general, the DREB/ERF subfamily genes evolved into distinct lineages and expanded with their own specificity after the separation of the four monocot plants, showing their conservation and divergence during evolution and in the process of adaptation to the environment. These results would be helpful for understanding the expansion mechanism of this DREB/ERF subfamily in the monocot plants.

### Tandem and segmental duplications contributed to BdAP2/EREBP expansion

**Figure 3:**
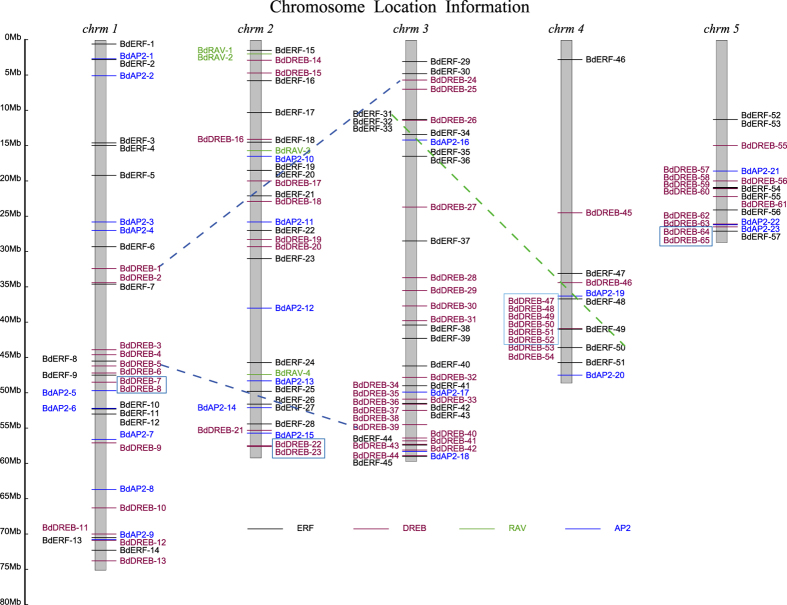
Chromosomal distributions of the *B. distachyon* AP2/EREBP genes. *chrm* is an abbreviation for chromosome. Tandem duplicated gene clusters were highlighted in boxes. The dotted lines, connecting the AP2/EREBP genes in different chromosomes, show the chromosomal segment duplication events. The genes in different subfamilies were color coded. AP2/EREBP is shorted for APETALA2/ethylene-responsive element binding protein.

The 149 BdAP2/EREBP genes scattered unevenly over the 5 chromosomes of *B. distachyon* (Fig. 3). A large portion (74.5%) of the 149 genes was located on chromosomes 1, 2 and 3. The majority of the BdAP2/EREBP genes on chromosomes 4 and 5 congregated closely to one end of the chromosomes. Interestingly, all of the BdRAV genes resided on chromosome 2. Such uneven distribution of these genes provided a clue to their evolution.

In plants, tandem and segmental duplications are known to be major forces for expansion of gene families^65^. Adopting the method and criteria we used previously^50^, we analyzed possible tandem and segmental duplication events in the *B. distachyon* AP2/EREBP family. We found that 12 BdDREB genes belonging to four gene clusters (genes in 4 boxes in Fig. 3, Table S4) were involved in tandem duplication. Two pairs of BdERF genes (*Bradi3g12565/Bradi4g38941* and *Bradi2g21060/Bradi2g52370*) and two pairs of BdDREBS genes (*Bradi1g47480/Bradi3g54160* and *Bradi1g36590/Bradi3g07450*) were implicated in segmental duplication. The largest gene cluster consisted of 6 tandem duplicated genes (the box on chromosome 4 in Fig. 3). Surprisingly, no duplication event was recognizable in the AP2 and RAV subfamilies under the relatively stringent criteria we used.

If the criteria for gene duplication were relaxed, i.e., with a lower similarity (≥40%) between amino acids sequences and a shorter sequence matching length (≥60%), more AP2/EREBP genes, including all members of the BdRAV subfamily and most genes clustered together in the phylogenetic tree of the BdAP2/EREBP proteins (Fig. 1), could be recognized as involved in segmental or tandem duplication. In other words, this result indicated that tandem and/or segmental duplications had great contributions to the expansion of the BdAP2/EREBP family. *BdDREB-53*, *BdDREB-54* and the 6 tandem duplicated genes could form a super gene cluster. Within this super cluster, *BdDREB-53* and *BdDREB-54* shared approximately 52% and 46% similarity of amino acids sequences with the other 6 tandem duplicated proteins, respectively, which were far below that within the 6 tandem duplicated genes. Interestingly, *BdDREB-51* of this super cluster together with the gene cluster consisting of *BdDREB-22* and *BdDREB-23* (the blue box on chromosome 2, Fig. 3) happened to be involved in segmental gene duplication (Table S4), which may be the reason that these duplicated genes were originated from a common ancestral chromosomal segment but subsequently diverged under different selective environmental pressures.

### The exon/intron organizations of BdAP2/EREBP genes are relatively simple

To obtain deep insight into the gene structures of the *B. distachyon* AP2/EREBP genes, their exon/intron organizations were analyzed and ranked based on the phylogenetic tree. All members of BdAP2 subfamily had four to nine introns, while the majority of members of BdRAV, BdDREB and BdERF subfamilies had no intron and the gene structures of them were relatively simple (Figure S4), which was corresponding with that in rice^7^. In particular, only 12 (18% of the total) DREBs and one (25%) RAV gene had introns. The number of introns of the two subfamilies members was small compared with that of BdAP2 subfamily and almost all of them with introns had only one intron except Bradi2g29960 and Bradi2g04000 which had 3 and 2 introns, respectively. For the BdERF subfamily, sixty-one percent of them had no intron and all the other members had only one intron. In addition, the exon/intron structures of 20 genes originated from tandem or segmental duplication and most of Ap2/EREBP members clustering together in the phylogenetic tree tended to share similar structure organizations (Figure S4).

### Synteny of AP2/EREBP genes across *B. distachyon* and other grass species

A genomic analysis of the synteny of this gene family across several grass plant species could provide insights to its evolution. We comparatively analyzed the synteny across *B. distachyon*, sorghum, maize and rice (Figure S5). The maximum orthology of the *B. distachyon* AP2/EREBP genes was found to be with sorghum (89, 60%), followed by maize (84, 56%) and rice (76, 51%); the result was consistent with the evolutionary relationships among these species. Several BdAP2/EREBP genes were syntenic with the same orthologs of the other grass plants. For example, both orthologs of a pair of duplicated genes (*Bradi4g38941/Bradi3g12565*) correspond to the same gene of sorghum, maize or rice, indicating the two genes may have evolved after *B. distachyon* was separated from the other three grass plants. Interestingly, many BdAP2/EREBP genes are biased towards particular chromosomes or chromosomal regions of sorghum, maize and rice, which may be caused by chromosomal rearrangement events, e.g., genomic duplication and inversion.

### Transactivation activity analyses of BdDREBs

**Figure 4:**
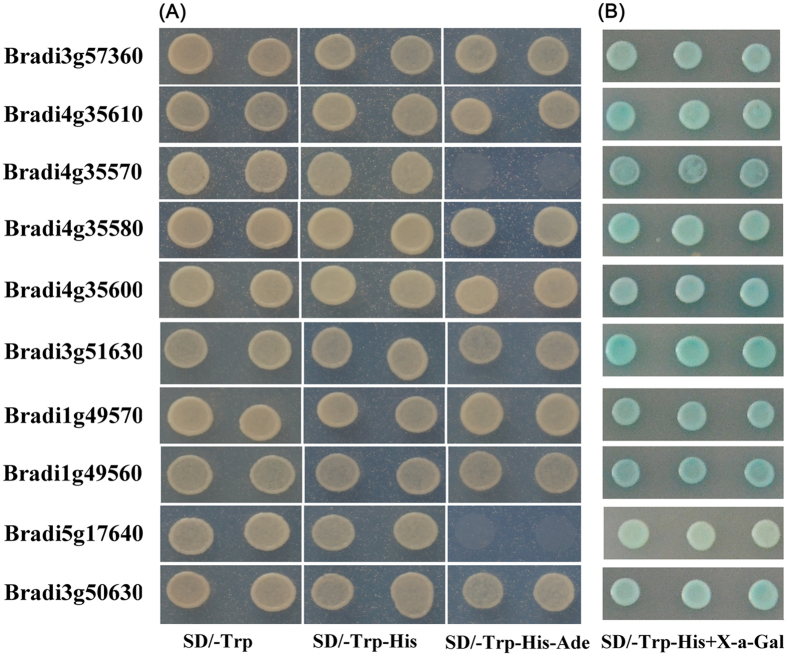
Transactivation activity assays of 10 *B. distachyon* DREB proteins. AH109 yeast cells, transformed with plasmid containing different BdDREB genes individually, were cultivated in nutritional selective medium (**A**) SD/-Trp, SD/-Trp-His, SD/-Trp-His-Ade or (**B**) SD/-Trp-His-X-α-gal. The empty vector pGBKT7 was negative control. Photos were taken after incubating at 28 °C for three days. DREB is shorted for dehydration responsive element binding gene.

The DREB proteins are important transcription factors for gene regulation in response to various environmental stimuli and the A1 group members of the DREB subfamily expand greatly in *B. distachyon*. To investigate the functional divergence of these genes, 10 closely related and representative members from the A1 subgroup were chosen for transactivation activity analysis (see Methods). As shown in Fig. 4, eight of the ten members tested grew well on the SD/-Trp-His-Ade medium, while Bradi5g17640 and Bradi4g35570 failed to show sustainable growth, indicating that they might have weak transactivation activities. Furthermore, the transactivation activities of the 10 BdDREBs were further assessed according to the growth status and color changes of yeast cells on the SD-Trp-His-X-gal medium for 3 d. All these proteins on the selective medium containing X-gal appeared blue (Fig. 4B), showing that they have obvious transactivation activities, but the single colony color of Bradi5g17640 was much weaker than the other nine, which was consistent with the result from the experiment of yeast survivability discussed above. Nevertheless, these results should not be interpreted as the members detected having definitive transactivation activities in plant. To address this issue, their transactivation activities should be further confirmed by other biological technologies such as ChIP (Chromatin Immunoprecipitation) or transient expression assays in *Nicotiana benthamiana*^66^.

### Involvement of BdAP2/EREBP genes in *B. distachyon* development

**Figure 5:**
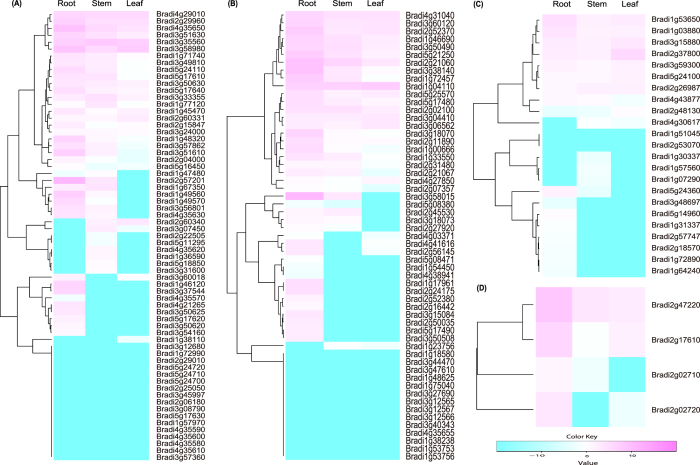
Expression profiles of BdAP2/EREBP (APETALA2/ethylene-responsive element binding protein) genes in three tissues of roots, stems and leaves. Expression profiles (in log_2_ based values) of the BdDREB (**A**), BdERF (**B**), BdAP2 (**C**) and BdRAV (**D**) genes in three tissues. Bd is shorted for *B. distachyon*. DREB, ERF, AP2 and RAV were shorted for dehydration responsive element binding gene, ethylene responsive factor, APETALA2 and related to ABI3/VP, respectively.

Profiling gene expression can gain insights into gene functions. We investigated the tissue-specific expressions of the 149 *BdAP2/EREBP* genes in three tissues, namely roots, stems and leaves, and under four abiotic stress conditions. The expressions of some of these genes varied significantly across the three tissues (Fig. 5, Table S5). For example, nine BdDREB genes such as *Bradi1g49560, Bradi1g49570* and *Bradi3g37544*) had higher expression in root than in the other two tissues, suggesting they may play vital roles in root development. In addition, the genes in the same subfamily had quite different expression profiles. For example, *Bradi3g04410*, *Bradi2g21060, Bradi2g52370, Bradi3g06562*, *Bradi5g21250* and *Bradi5g25570*) in the ERF subfamily were expressed abundantly, whereas the expressions of several other BdERF genes such as *Bradi3g47610*, *Bradi3g40343* and *Bradi2g16442* were barely detectable in the 3 tissues (Fig. 5, Table S5). Similar observations could be made on the other BdAP2/EREBP subfamily genes. These results indicated that the dynamic expression change of each BdAP2/EREBP gene was correlated with its specific function. In addition, closely related genes generally showed highly similar expression profiles (Table S5), further supporting that they may have similar or overlapping functions.

Moreover, to investigate the reliability of deep sequencing based expression profiling, the expressions of 32 representative members of the BdDREB subfamily, which play import roles in development and in response to external stimuli and expand greatly in *B. distachyon,* were analyzed by RT-PCR. Among the 32 BdDREB genes, twelve were selected from the biggest subgroup A1 and four were chosen from each of the other five subgroups of the DREB subfamily. However, all the members of the A3 subgroup could not be detected by RT-PCR, in agreement with their low abundance detected by RNA-seq profiling. The remaining 28 representatives were profiled by RT-PCR. The result showed that the majority of the 28 BdDREB genes had similar expression profiles in all three tissues using the two profiling methods (Figure S6). However, several genes exhibited different expression patterns between the two methods. For example, *Bradi4g35580, Bradi4g35600*, *Bradi1g57970*, *Bradi3g45997* and *Bradi2g25050* were expressed at a low level in some tissues using RT-PCR, but were barely detected by deep sequencing. This discrepancy reflected the difference and biases of the two profiling platforms. Nevertheless, despite such difference in profiling, the results were overall consistent.

### Many BdAP2/EREBP genes were functional under abiotic stresses

To appreciate the potential functions of the BdAP2/EREBP genes under environmental stresses, we investigated their expressions under cold, heat, drought and high-salinity conditions. A total of 147 (98.7% of the 149) BdAP2/EREBP genes (56 BdERFs, 64 BdDREBs, 4 BdRAVs and 23 BdAP2s) were subjected to expression analyses under these stresses, as they were probed on the Affymetrix *Brachypodium* Genome Array (BradiAR1b520742 chip, see Methods).

**Figure 6:**
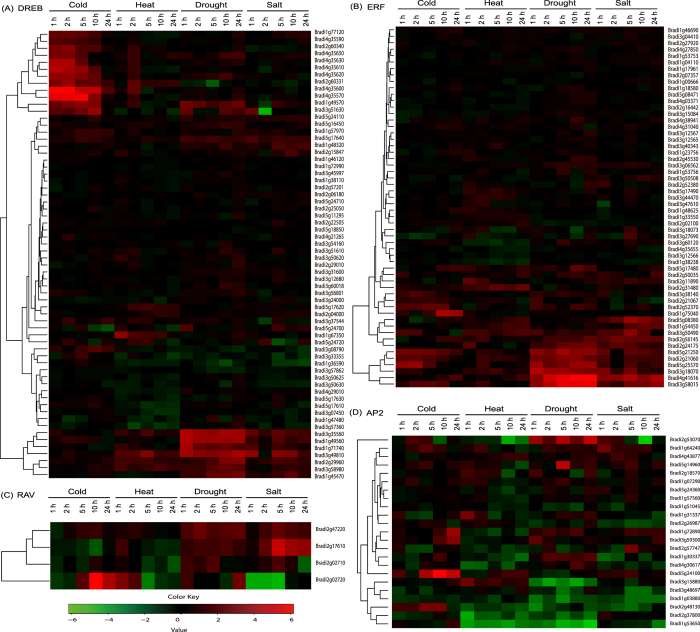
Expression profiles (in log_2_ based fold change) of BdDREB (**A**), BdERF (**B**), BdRAV (**C**) and BdAP2 (**D**) genes under four abiotic stress conditions. Bd is shorted for *B. distachyon*. DREB, ERF, AP2 and RAV were shorted for dehydration responsive element binding gene, ethylene responsive factor, APETALA2 and related to ABI3/VP, respectively.

The result revealed that the BdDREB subfamily genes could be classified into 3 subgroups based on their expression profiles (Fig. 6A, Table S6). Except the ones in the middle subgroup, the genes in the other two subgroups were all differentially expressed under at least one of the stresses considered. Among the 64 BdDREBs, 12 might be involved in cold signaling pathways (Fig. 6B), consistent with a previous report^1^. For example, the tandem duplicated genes of *Bradi2g60340*, *Bradi2g60331*, *Bradi4g35570, Bradi4g35590, Bradi4g35600, Bradi4g35610, Bradi4g35620* and *Bradi4g35630* in the first subgroup were significantly up-regulated at all the first three time points after cold treatment. *Bradi4g35600*, particularly, exhibited quick cold responses with the highest expression abundance at 1 h point after cold stress, indicating that it may be an early response factor of cold. *Bradi4g35630*, also known as Cold-Binding Factor 1 (BdCBF1), has been shown to be significantly up-regulated under cold stress^55^. Transgenic *Brachypodium* plants overexpressing *Bradi4g35630* also exhibited enhanced resistance to drought and salt stresses as well as low temperatures^55^. These results, plus *Bradi4g35630* being a transcription factor, evidently showed *Bradi4g35630* as a regulator of stress response, particularly of cold response. Moreover, not only a single BdCBF1, i.e., *Bradi4g35630,* more than half of BdCBF genes in the A1 subgroups (Fig. 2) respond to cold stress at different developmental stages of *B. distachyon*^54^,^67^, showing their critical roles in cold response.

Compared with the differentially expressed genes (DEG) of the BdDREB subfamily induced mainly by cold, the DEGs of the BdERF subfamily were induced mainly by drought and salt stresses (Fig. 6B), suggesting their potential roles in resistance to drought and salt. In particular, the genes (*Bradi2g56145*, *Bradi2g24175*, *Bradi5g21250, Bradi2g21060*, *Bradi5g25570*, *Bradi3g18070, Bradi4g41616* and *Bradi3g58015)* in the last subgroup of the heatmap were significantly up-regulated during the whole period of drought stress profiled except *Bradi2g56145* at the 1 h point. Interestingly, *Bradi4g41616* and *Bradi3g58015* were also significantly up-regulated at all the time during the salt stress. The two genes may be involved in the same pathway or have redundant or complementary functions in response to the two stresses. For these BdERF genes with differential expressions, they might be selected as potential candidate genes for further functional validation and for utilization in crop improvement plants.

RAV genes have been shown to be participated in plant growth and development^68^, leaf maturation, and senescence^69^, and to control the flowering time under long-day growth conditions^70^. They have also been shown to be responsive to biotic and abiotic stresses^25^,^27^,^71^,^72^,^73^, which were supported by our results as well. For example, *Bradi2g17610* was significantly induced at 5 h point after salt treatment (Fig. 6C); similar observation can be made on its paralogs *Bradi2g47220* under salt stress condition, indicating that the two genes might be positive regulators of salt stress in *B. distachyon* as their orthologs in *Capsicum annuum*^71^ and *Arabidopsis*^72^. For the two other BdRAV genes, the expression of *Bradi2g02720* gradually increased over time and reached a peak at 10 h point after cold treatment, and was significantly down-regulated by the first three points of salt treatment. However, the expression of *Bradi2g02710* had little change across the four stress conditions except that at 24 h point after salt treatment. Such diverse responsive behavior of the four BdRAVs suggested their distinct functions and their functions in different signaling pathways.

In contrast to the effort into the three subfamilies discussed above, the work on BdAP2 genes in response to abiotic stress was limited. As was shown in the heatmap (Fig. 6D), the DEGs of the BdAP2 subfamily were also few under the four stress conditions. Several genes were significantly down-regulated by at least one stress. For example, the expression of *Bradi1g53650* decreased at all the time points after heat and drought stresses. However, there was no single gene significantly induced at all the time points after the corresponding stress treatment.

### Comparison of DREB/ERF subfamily gene expressions in three monocot plants

DREB/ERF subfamilies play important roles in response to abiotic stresses such as cold, drought, salt and heat. The expression profiling of the genes in the two subfamilies in the four representatives (rice, sorghum, wheat (Winter Manitou) and *B. distachyon*) of the Poaceae would help understand the role of these transcription factors in response to abiotic stresses. However, the transcriptome data of sorghum under abiotic stresses were not publicly available. At last three species of wheat, rice and *B. distachyon* were selected for the expression analysis of DREB/ERF subfamily genes.

Expression profiling of wheat DREB/ERF subfamily genes was examined under cold, drought and heat stresses. A total of 20 TaDREBs and 22 TaERFs corresponding to 42 probe sets were identified in the three experiments. Details of the analytic result were provided in Table S7. As shown in Figure S7, the heatmaps of the expressions of both TaDREBs and TaERFs were grouped into three groups. All TaERF genes of group I (Figure S7B) were significantly up-regulated at 42 d point after cold treatment. Three TaDREBs (*Ta.41731*, *Ta.41203* and *Ta.34828*) of DREB group I were significantly up-regulated at least at one time point of cold treatment (Figure S1). The paralogs of the three up-regulated genes, *TaCBF14* and *TaCBF15*, happened to be involved in the cold acclimation process and in frost tolerance^74^. The expression of *Ta.45422* in the group IIId of A1 group also increased, although less than twofold compared with the control. Interestingly, its orthologs in *B. distachyon* grouped together in the subgroup IIId (Figure S1) were all up-regulated significantly at least at three points after cold stress; while the expression of its paralogs in wheat were not profiled since the probe sets of these genes were not on the microarray used. These results suggested functional conservation and divergence of DREBs during the Pooideae evolution. In addition, the transcripts of three DREBs (*Ta.13471*, *Ta.920* and *Ta.57800*) and five TaERFs of group III (*Ta.17378*, *Ta.33090*, *Ta.41713*, *Ta.14000* and *Ta.41713*) (Figure S7) significantly declined at least at one time point of cold treatment, especially *Ta.13471*. However, none of TaDREB/ERF genes have significant differential expression under drought stress. There are only two TaDREBs (*Ta.49353* and *Ta.5364*) and two TaERFs (*Ta.33090* and *Ta.35532*) with obvious change in the expression under the heat stress. Generally, wheat had more differentially expressed genes under cold stress than under heat and drought stresses, which may be correlated with that winter wheat need to develop more DREBs to survive the cold winter.

Similarly, the two subfamily genes were also investigated in rice using the available microarray gene expression data under cold, drought and salt stress conditions. Thirty OsDREB/ERF genes (22.4% of 134 OsEREB/ERF members) including 12 OsDREBs and 18 OsERFs were significantly induced under drought stress (Figure S8 and Table S8), while only 3 OsDREB and one OsERF genes were repressed in this stress condition. Of the 12 induced OsDREB genes, five (*Os06g07030*, *Os04g34970*, *Os02g52670*, *Os08g31580* and *Os03g09170*) happened to be significantly induced by salt. A similar observation was made on OsERF subfamily. Particularly, five OsERF members (*Os01g54890*, *Os02g43790*, *Os05g34730*, *Os11g06770* and *Os05g29810*) were significantly induced under drought and salt stresses, suggesting their vital roles in regulating stress response. In addition, of the 18 OsERF genes induced by drought, 6 were evenly distributed across all subgroups of B1 group (Figure S3) except the subgroup VI, and 7 genes were in the B4 group. Interestingly, four of the 7 induced genes clustered together with their orthologs in *B. distachyon* in the subgroup I of B4 group (Figure S3) and these orthologs were also significantly induced by drought in *B. distachyon*, while their only one othologs in wheat did not show similar expression change, indicating the functional conservation and divergence of these orthologs in evolution. Compared with the differentially expressed genes under drought stress, the number (12) of OsDREB/ERF genes with differential expression was relatively small under salt stress and no single OsDREB/ERF gene was significantly down-regulated.

Expression analysis of the DREB/ERF subfamily genes in wheat, rice and *B. distachyon* under cold, high-salinity, drought or heat stresses revealed diverse differential expression of these genes and their possible roles in response to these stresses. Especially in *B. distachyon*, 11 BdDREB genes that clustered together in the subgroup IIId of A1 group (Figure S1) and were all significantly induced by cold could be further studied and be exploited to enhance stress tolerance. In summary, these results would help expand our knowledge of this important class of transcription factors in plant stress resistance. Some of the results might be utilized to explore the mechanisms of gene regulation under abiotic stresses.

### Functional divergence of duplicated BdAP2/EREBP genes in evolution

Gene duplication is an essential source of genetic novelty that can lead to evolutionary innovation. Three possible outcomes have been suggested for the evolution of gene duplication: subfunctionalization, neofunctionalization and non-functionalization^75^. Profiling gene expression may provide an ample amount of information of the mode and tempo of duplicated genes. We thus compared the expression patterns of duplicated genes under cold, heat, drought and salt stresses.

**Figure 7:**
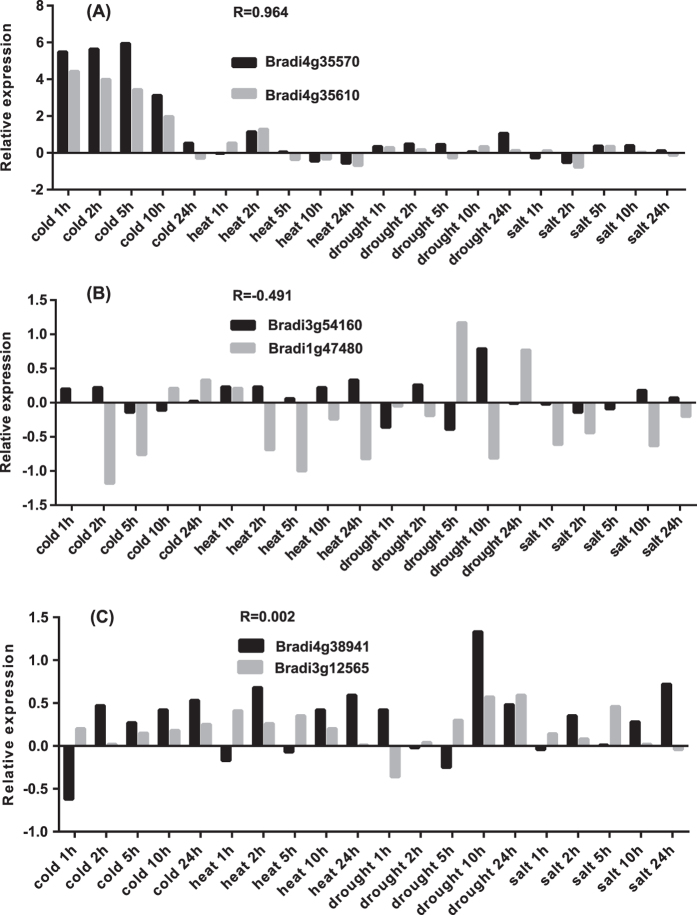
Comparisons of expression profiles of three representative pairs of duplicated AP2/EREBP genes in *B. distachyon* across 20 time points (in hours) after cold, heat, drought and high-salinity stress treatments. Represented in y-axes are the expressions, in log_2_ based fold changes of the corresponding treatments. The correlation (R) between two duplicated genes is measured by the Pearson correlation coefficient of the expressions of the pair of genes. AP2/EREBP is shorted for APETALA2/ethylene-responsive element binding protein.

Our analysis focused on 19 (95%) of the 20 duplicated genes that were profiled by the microarray gene expression data. Among the 19 genes, a gene cluster consisting of five tandem duplicated genes (the box on chromosome 4, Fig. 3) had highly similar expression patterns (Table S6), indicating subfunctionalization during evolution. Specifically, all the pairs of the five duplicated genes had positive expression correlations, measured by the Pearson correlation coefficients (PCCs), being greater than 0.5, and there was one extreme case of the *Bradi4g35570*/*Bradi4g35610* pair with the PCC value greater than 0.96 (Fig. 7A). Such high correlations among the five duplicated genes suggested subfunctionalization of the paralogs through division of labor by retaining different parts (subfunctions) of their original ancestral functions through gene duplication.

Note that out of the 17 pairs of the duplicated genes, 13 were positively correlated with positive PCC values. However, the most noticeable were the two pairs, *Bradi3g54160/Bradi1g47480* (PCC, −0.491) and *Bradi5g24720/Bradi5g24710* (PCC, −0.156), that had negative PCC values (Fig. 7B). In addition, two pairs of duplicated genes, *Bradi1g36590/Bradi3g07450* (PCC, 0.361) and *Bradi4g38941/Bradi3g12565* (PCC, 0.002), had small PCC values (Fig. 7C), exhibiting little correlation in gene expression across the four abiotic stresses. This implied a neofunctionalization as one copy of the pair acquired, after duplication, a novel and beneficial function that was preserved by natural selection, with the other copy retaining its ancestral function. Moreover, under loose criteria for gene duplication, more BdAP2/EREBP genes would be identified as being produced via gene duplication. For example, under the relaxed criteria of a lower similarity (≥40%) between amino acids sequences and a shorter sequence matching length (≥60%) as mentioned earlier, *Bradi4g35630* and *Bradi4g35650* would be paralogs. Some of these duplicated genes still had obviously positive correlation in gene expression, although their amino acid sequences had a lower similarity. For example, the expressions of *Bradi4g35630* and *Bradi4g35650* with 47% similarity in amino acid sequences had a PCC of 0.89, indicating the functional conservation of the two genes.

In short, the expression correlation analysis on duplicated genes revealed their functional roles for subfunctionalization and neofunctionalization in the expansion of BdAP2/EREBP superfamily. In other words, the functions of the superfamily genes were expanded and enhanced through gene duplication.

## Materials and Methods

### Database search and sequence retrieval

The *B. distachyon* genome assembly and annotation version 3.0 (http://genome.jgi.doe.gov/pages/dynamicOrganismDownload.jsf?organism=Bdistachyon) were used, and the putative genes of the AP2/EREBP family in *B. distachyon* were extracted following the strategy for *Setaria italica*^19^ and *Vitis vinifera*^16^. The AP2/EREBP family gene sequences in *Arabidopsis* and rice were downloaded from the *Arabidopsis* Information Resource (TAIR, http://www.arabidopsis.org/, release 10.0) and the rice genome annotation database (http://rice.plantbiology.msu.edu/, release 7.0), respectively. Blast search was then performed using sequences of *Arabidopsis* and rice AP2/EREBP genes as queries to search for putative AP2/EREBP genes in *B. distachyon* as described in *Vitis vinifera*^16^. At the same time, we also used the Hidden Markov Model (HMM) profile of the AP2/EREBP domain (PF00847, from the Pfam database http://pfam.xfam.org/) to search the *B. distachyon* protein sequences. All hits with expected (E) values less than 1.0^19^ were retained and further manually analyzed using InterProScan program (http://www.ebi.ac.uk/Tools/InterProScan/). Finally, the resulting putative AP2/EREBP genes identified by the above two strategies were further verified with available ESTs (http://www.plantgdb.org/download/download.php?dir=/PublicPlantSeq/Dump/B/Brachypodium_distachyon/FASTA) (Table S1). For phylogenic analysis of this superfamily in Poaceae, the AP2/EREBP family gene sequences in sorghum were downloaded from Phytozome (http://phytozome.jgi.doe.gov/pz/portal.html#!info?alias=Org_Sbicolor_er). The *T. aestivum* EST sequences were retrieved from the website of the National Center for Biotechnology Information (Ta.seq.all.gz), and then assembled and translated into amino acids as described earlier as for wheat^60^.

### Phylogenetic analysis and sequence alignment

The identified AP2/EREBP sequences or domain sequences of *B. distachyon* and other plants were aligned against that of *Arabidopsis* and rice using Clustal 2.0^76^ with default parameters. Phylogenetic trees were constructed based on the bootstrap neighbor-joining (NJ) method and bootstrap analysis (1,000 replicates) by MEGA^77^.

### Chromosomal distribution, gene structure, gene duplication and synteny analysis

The exon/intron structures and chromosomal distribution of *B. distachyon* AP2/EREBP genes were determined using the Blat search tool^78^ and CD-HIT^79^. Gene duplication events were analyzed according to our previous description^50^. Tandem duplicated genes were defined as neighboring homologous AP2/EREBP genes on the same chromosome of *B. distachyon*, with no more than one intervening gene^80^. For synteny analysis of this gene family across sorghum, maize, rice and *Brachypodium*, synteny blocks among them were downloaded from the Plant Genome Duplication Database^81^ and those containing BdAP2/EREBP genes were identified. The orthologs relationships among these four plants were displayed using Circos^82^.

### cDNA cloning, plasmid construction and transcriptional activation analysis in yeast cells

Ten closely related and representative BdDREBs were selected to be amplified by RT-PCR, with primers designed based on the predicted sequences and cloned into pMD18-T vectors for sequencing. They were then fused to the GAL4 DNA binding-domain vector pGBKT7 to construct plasmid pGBKT7-BdDREBs for transactivation assays. All of the primers used for RT-PCR, cDNA cloning and expression studies were listed in Table S9. Transcriptional activation assays were performed in the yeast strain AH109 with *Ade2* and *His* reporter genes. The PCR products of these genes were cloned separately into the pGBKT7 vector. The constructed BdDREB vectors along with the pGBKT7 plasmid as a negative control were transformed into yeast strain AH109 following the manufacturer’s protocol (Clontech, USA). Transformed strains were confirmed by PCR and then plated on selective synthetic dropout (SD) media without tryptophan (SD/-Trp), without tryptophan and histidine (SD/-Trp-His), or without tryptophan, histidine, and adenine (SD/-Trp-His-Ade), to determine their survivability. In addition, the transcription activation of these genes was also evaluated based on growth status and color variation of yeast cells on incubating plates with SD/-Trp-His-X-gal for 3 d. All transcriptional activation assays were performed in triplets.

### Tissue-specific expression profiling using RNA-seq and RT-PCR

Roots, stems and leaves of over three-week old Bd21 were collected and stored separately for RNA isolation for tissue-specific expression analysis. Ten samples for each tissue randomly selected from ten individual plants were pooled together for RNA extraction. Sequencing using SOLiD™ 5500 next-generation sequencing platform, with main reagents and supplies in SOLiD^TM^ Total RNA-Kit (Life Technologies), and preliminary data acquisition were performed in house. The alignment of RNA-seq reads to *B. distachyon* genome (version 3.0) was performed by TopHat (v2.0.13)^83^. The cuffquant and cuffnorm functions of Cufflinks (v2.2.1)^84^ were applied to compute fragments per kilobase of exon per million fragments mapped (RPKM) of each transcript and the cuffdiff function was used to determine the significantly differential expressed genes (q-value ≤ 0.05). Tissue-specific expression profiles were visualized in heatmaps using R packages (version 2.15.1), based on the log_2_-based RPKM values of genes in different tissues.

For expression analysis of some BdAP2/EREBPs using RT-PCR, total RNA was isolated with Trizol reagent (Invitrogen) following the manufacture’s protocols and purified using an RNeasy Plant Kit (Qiagen). The integrity and purity of RNA samples were checked by gel electrophoresis and OD 260/280 nm absorption ratios and the RNA concentration was quantified using a micro-volume spectrophotometer (Quawell Technology). RT-PCR analysis was performed as described previously^50^.

### Expression analysis of AP2/EREBP genes in *B. distachyon*, rice and wheat under abiotic stresses

Gene expression profiling of *B. distachyon,* wheat, and rice under drought, high-salinity, cold, or heat stress conditions^67^ were downloaded from ftp://brachypodium.org/brachypodium.org/Stress/(dataset ID BradiAR1b520742) and Gene Expression Omnibus (dataset ID: GSE23889, GSE70443, GSE60351 and GSE6901). These data from Affymetrix microarray experiments were analyzed following the method previously described^67^. Briefly, the probes were aligned to the *B. distachyon* reference genome (version 3.0), cDNA sequences of rice AP2/EREBP genes or unigenes of wheat by Burrows-Wheeler Aligner (BWA)^85^, and the probes that had no mismatches in the alignment and were uniquely mapped to the exonic region of a unique gene were retained for further probe-set analysis. Probe set summarization and expression estimates for each gene were conducted using the apt-probeset-summarize tool (version 1.16.0) from Affymetrix^67^. Expression intensities under the normal, drought, high-salinity, cold, and heat conditions were used for expression analysis of AP2/EREBP genes under abiotic stresses. A gene was called up- or down-regulated across two conditions (e.g., cold vs normal condition) if the log_2_-based fold change of its expression abundance across the two conditions was greater than 1 or less than −1 as done in our previous report^50^. The significance levels of the differential expression genes were estimated by an R package ‘samr’ and genes with q-values ≤ 0.05 were deemed as significant. Hierarchical clustering of expression profiles of *B. distachyon* AP2/EREBP genes was performed using R packages (version 2.15.1).

## Additional Information

**How to cite this article**: Chen, L. *et al*. Expansion and stress responses of AP2/EREBP superfamily in *Brachypodium Distachyon*. *Sci. Rep.* **6**, 21623; doi: 10.1038/srep21623 (2016).

## Supplementary Material

Supplementary Information
