# Supplementary material for: Expansion and stress responses of AP2/EREBP superfamily in Brachypodium Distachyon
Source: Sci Rep. 2016 Feb 12;6:21623. doi: 10.1038/srep21623 (PMC4751504; doi:10.1038/srep21623)
Supplement: Supplementary Information [file srep21623-s1.pdf]

**Expansion and stress responses of AP2/EREBP superfamily in *Brachypodium Distachyon***

Lihong Chen[1a], Jiapeng Han[2a], Xiaomin Deng[3a], Tan shenglong[4], Lili Li[1], Lun Li[1], Junfei Zhou[1], Hai Peng[1], Guangxiao Yang[2], Guangyuan He[2]*, Weixiong Zhang[1,5]*

[1] The Institute for Systems Biology, Jianghan University, Wuhan 430056, China.

[2] The Genetic Engineering International Cooperation Base of Chinese Ministry of Science and Technology, Chinese National Center of Plant Gene Research (Wuhan) HUST Part, College of Life Science and Technology, Huazhong University of Science & Technology (HUST), Wuhan 430074, China.

[3] Ministry of Agriculture Key Laboratory of Biology and Genetic Resources of Rubber Tree/State Key Laboratory Breeding Base of Cultivation and Physiology for Tropical Crops, Rubber Research Institute, Chinese Academy of Tropical Agricultural Sciences, Danzhou 571737, China.

[4] School of Information Engineering, Hubei University of Economics, Wuhan 430205, China.

[5] Department of Computer Science and Engineering and Department of Genetics, Washington University, St. Louis, MO 36130, USA.

[a] These authors contributed equally to this work.

[*] Correspondence: weixiong.zhang@wustl.edu; hegy@mail.hust.edu.cn

**Supplementary figure and table legends**

**Supplemental Figures**

**Figure S1.** Phylogeny of A1 group of DREB subfamily genes of four monocot plants. Bd, Ta, Os and Sb were the abbreviations of *B. distachyon*, *Triticum estivum*, *Oryza sativa* and *Sorghum bicolor*. DREB was shorted for dehydration responsive element binding gene. Different species genes were highlighted with different color circles before each gene name. Different subgroups of DREB proteins are highlighted in different colors.

**Figure S2.** Phylogeny of each group (A2-A6) of DREB subfamily genes of four monocot plants. Bd, Ta, Os and Sb were the abbreviations of *B. distachyon*, *Triticum estivum*, *Oryza sativa* and *Sorghum bicolor*. DREB was shorted for dehydration responsive element binding gene. Different species genes were highlighted with different color circles before each gene name. Different subgroups of DREB proteins are highlighted in different colors.

**Figure S3.** Phylogeny of each group (B1-B6) of ERF (ethylene responsive factor) subfamily genes of four monocot plants. Bd, Ta, Os and Sb were the abbreviations of *B. distachyon*, *Triticum estivum*, Oryza sativa and *Sorghum bicolor*. Different species genes were highlighted with different color circles before each gene name. Different subgroups of ERF proteins are highlighted in different colors.

**Figure S4.** The exon/intron structural organizations of *B. distachyon* AP2/EREBP genes. AP2/EREBP was shorted for APETALA2/ethylene-responsive element binding protein.

**Figure S5.** The synteny relationships of AP2/EREBPs across *B. distachyon* and three other close related grass species including rice (A), maize (B) and sorghum (C). AP2/EREBP was shorted for APETALA2/ethylene-responsive element binding protein.

**Figure S6.** The comparisons of expression profiles of 28 *B. distachyon* DREBs (dehydration responsive element binding genes) between the RT-PCR (**A**) and deep sequencing (**B**). DREB was shorted for dehydration responsive element binding gene.

**Figure S7.** Expression profiles (in $\log_2$ based fold change) of wheat DREB/ERF subfamily genes under three abiotic stress conditions. DREB and ERF were shorted for dehydration responsive element binding gene and ethylene responsive factor.

**Figure S8.** Expression profiles (in $\log_2$ based fold change) of rice DREB/ERF subfamily genes

under three abiotic stress conditions. DREB and ERF were shorted for dehydration responsive element binding gene and ethylene responsive factor.

**Supplemental Tables**

Table S1. Annotation of the *B. distachyon* AP2/EREBP (APETALA2/ethylene-responsive element binding protein) gene family.

Table S2. The comparison *of B. distachyon* CBFs (C-repeat binding factors) among three papers.

Table S3. Summary of the AP2/EREBP family of *Arabidopsis*, sorghum, rice, wheat and *B. distachyon*. AP2/EREBP was shorted for APETALA2/ethylene-responsive element binding protein.

Table S4. The *B. distachyon* AP2/EREBPs genes derived from gene duplication. AP2/EREBP was shorted for APETALA2/ethylene-responsive element binding protein.

Table S5. The expression profiles (log2-based RPKM and q values) of *B. distachyon* AP2/EREBPs (APETALA2/ethylene-responsive element binding proteins) in different tissues.

Table S6. The expression profiles (log2-based fold change and q values) of *B. distachyon* AP2/EREBPs (APETALA2/ethylene-responsive element binding proteins) under different treatments.

Table S7. The expression profiles (log2-based fold change and q values) of wheat DREB/ERF (dehydration responsive element binding gene/ethylene responsive factor) subfamily genes under different treatments.

Table S8. The expression profiles (log2-based fold change and q values) of rice DREB/ERF (dehydration responsive element binding gene/ethylene responsive factor) subfamily genes under different treatments.

Table S9. Primers for (A) gene amplification and pGBKT7 vector construction and (B) RT-PCR to investigate the expression profiles of some *B. distachyon* AP2/EREBPs genes in different tissues. AP2/EREBP was shorted for APETALA2/ethylene-responsive element binding protein.

Supplementary Figure 1

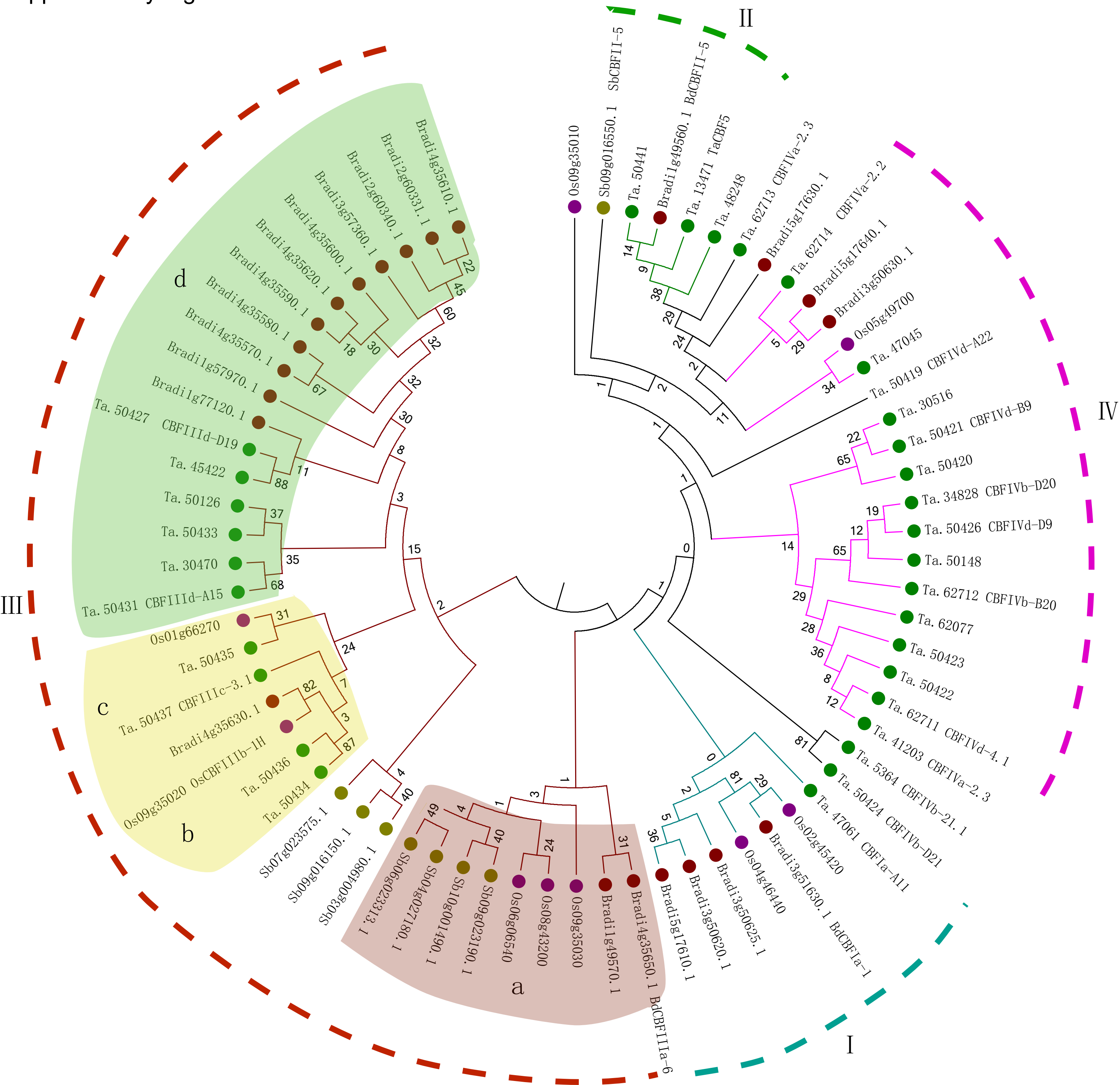

Supplementary Figure 2

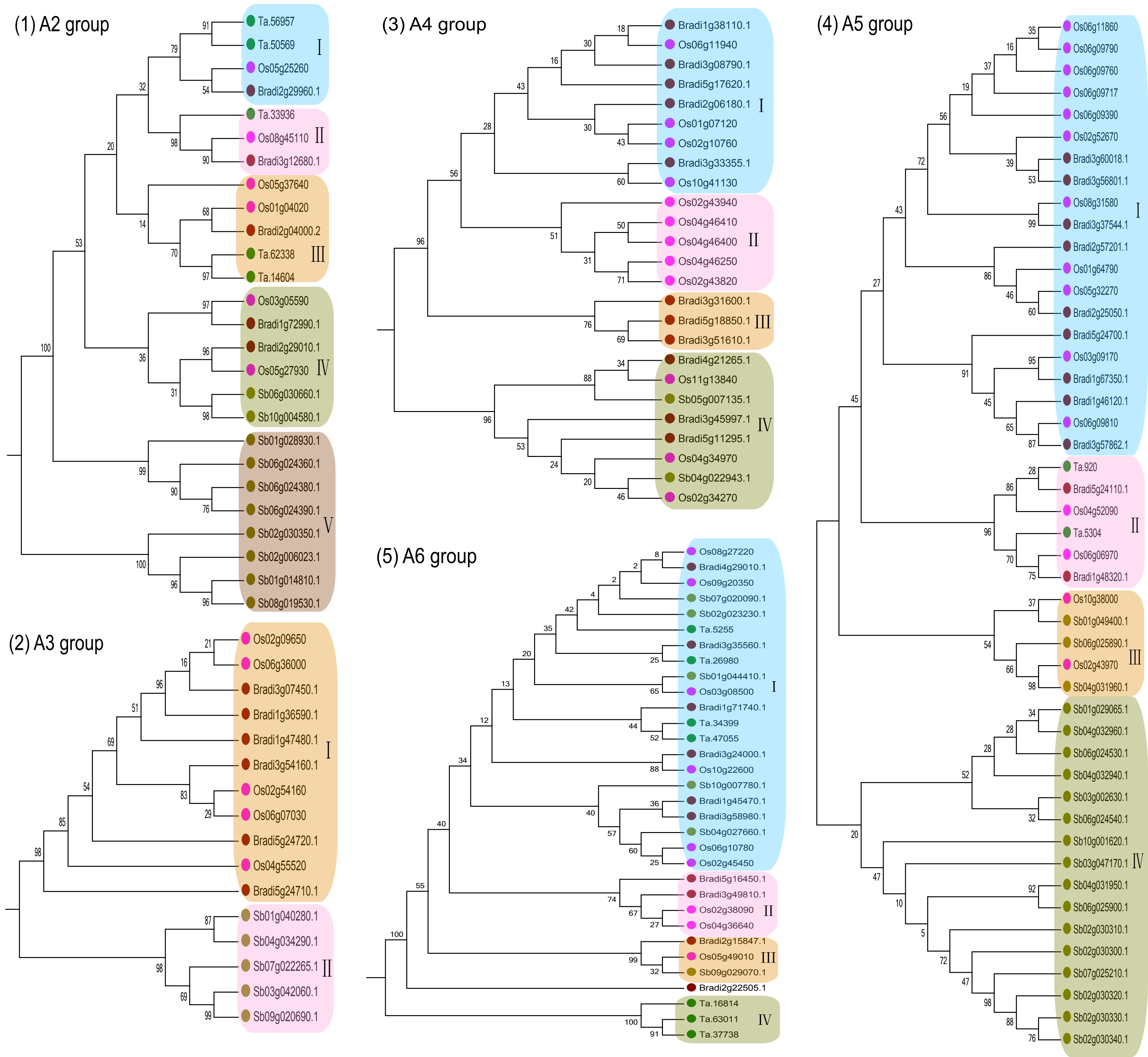

Supplementary Figure 3

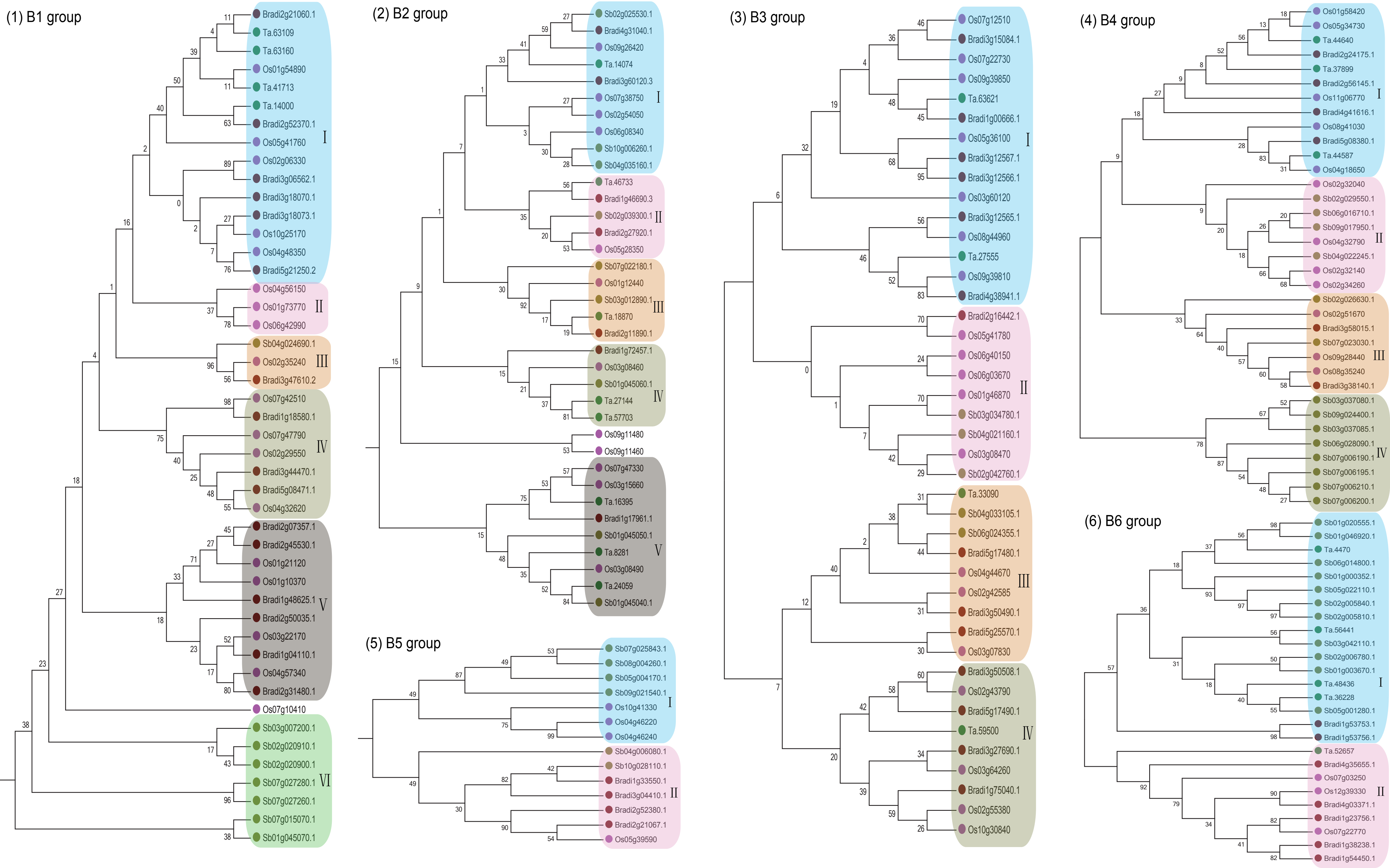

Supplementary Figure 4

# Exon and Intron Information

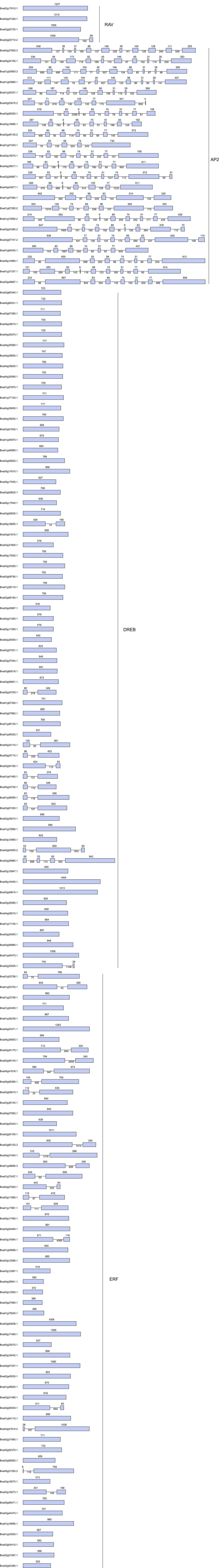

Supplementary Figure 5

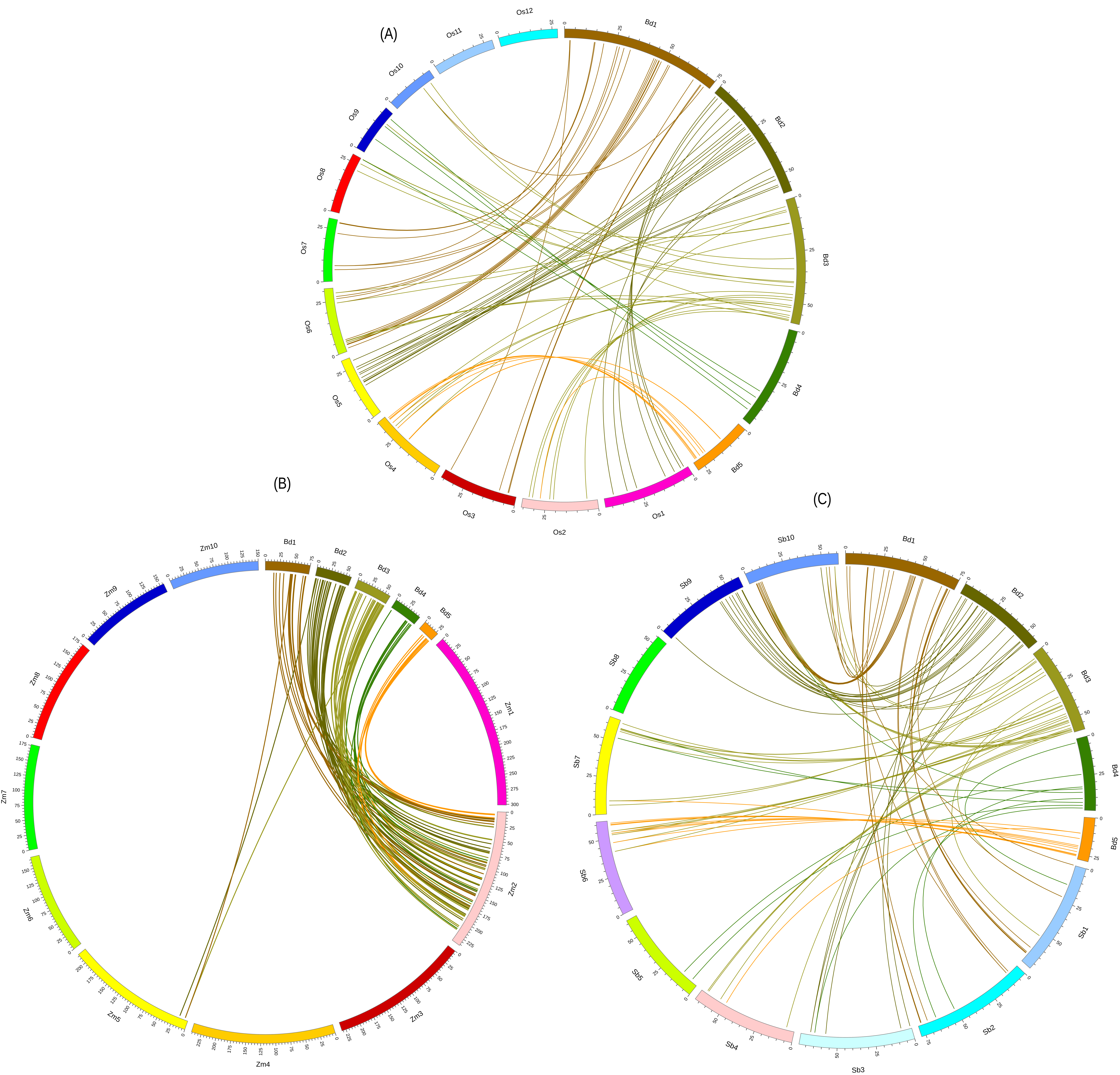

**Supplementary Figure 6**

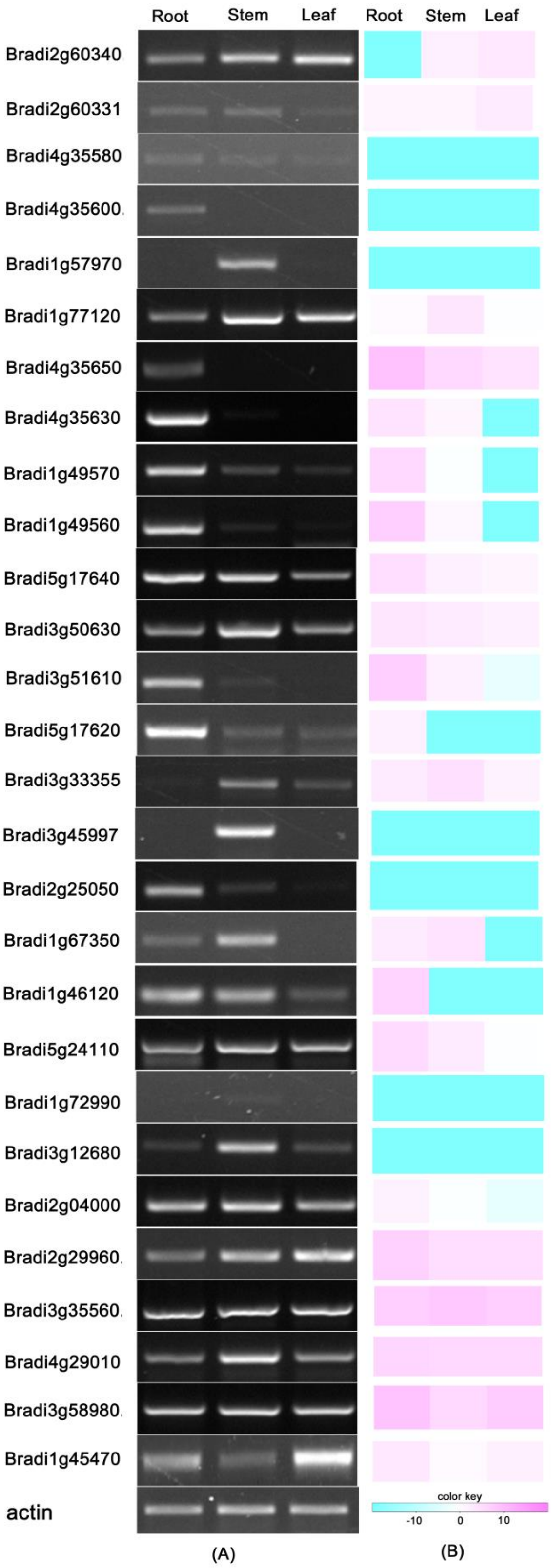

Supplementary Figure 7

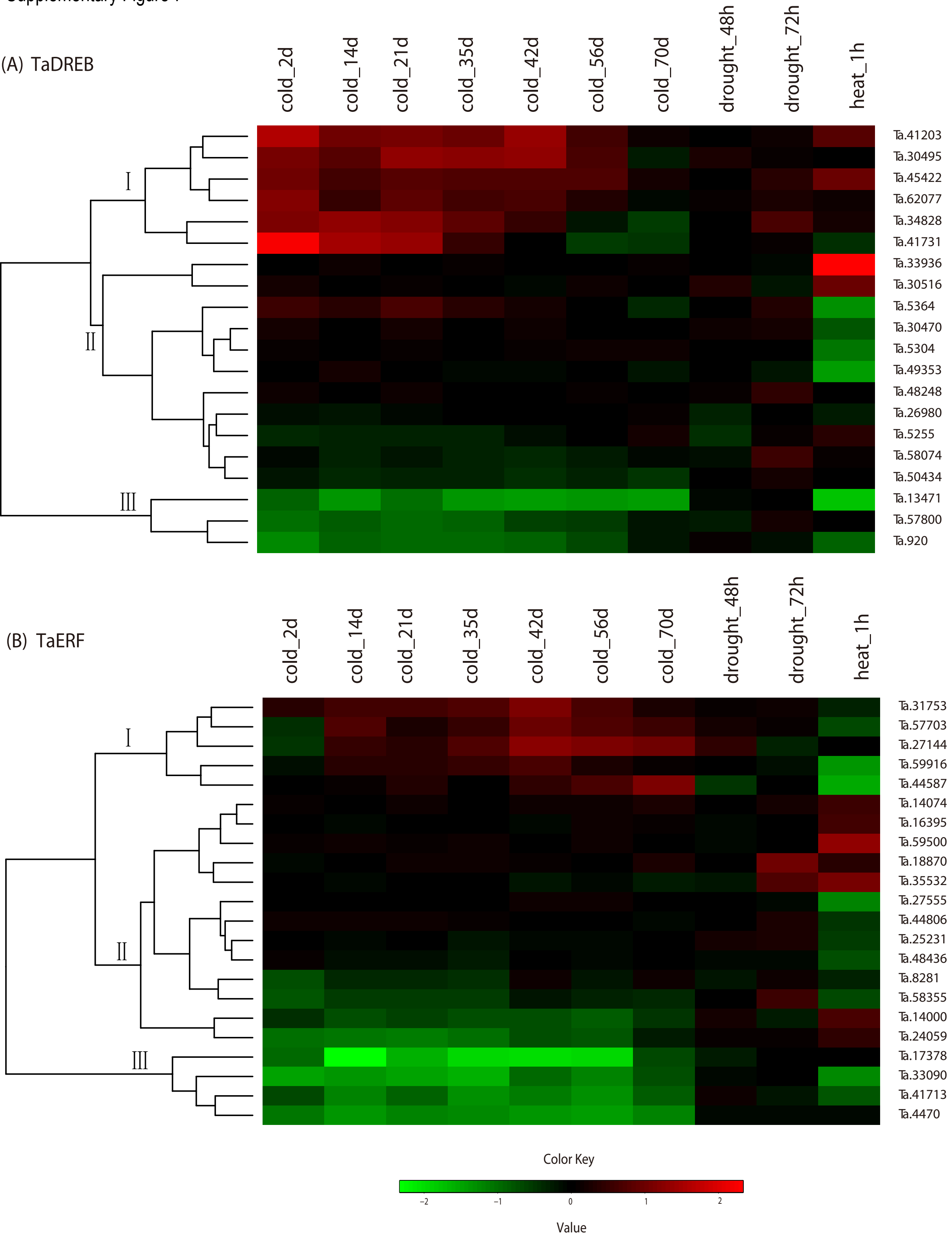

Supplementary Figure 8

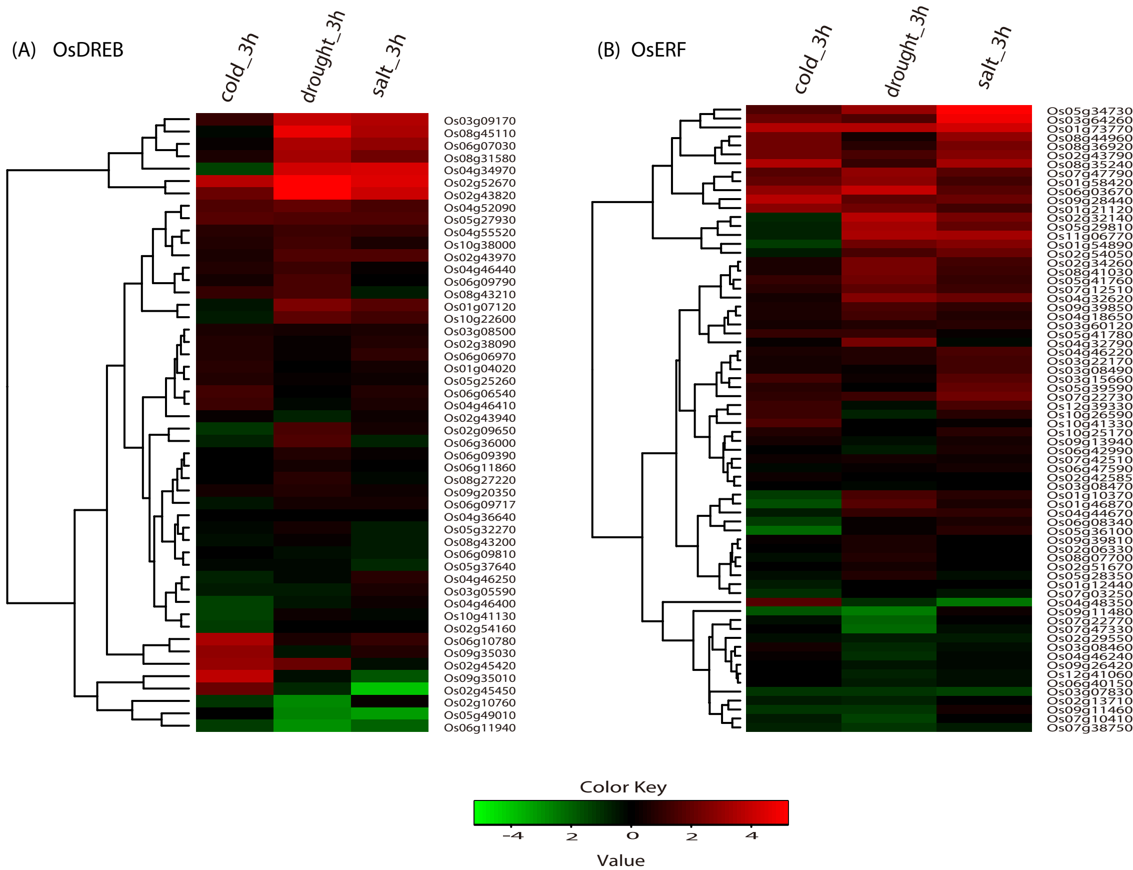

Supplementary Table 1: Annotation of the *Brachypodium distachyon* AP2/EREBP gene families.

| subfamily | gene model | gene name | chromosome | The amino acid length | ESTs |
|---|---|---|---|---|---|
| RAV | Bradi2g17610 | BdRAV-3 | 2 | 408 | 0 |
| | Bradi2g47220 | BdRAV-4 | 2 | 403 | 2 |
| | Bradi2g02720 | BdRAV-2 | 2 | 364 | 0 |
| | Bradi2g02710 | BdRAV-1 | 2 | 374 | 0 |
| AP2 | Bradi2g37800 | BdAP2-12 | 2 | 493 | 4 |
| | Bradi5g24100 | BdAP2-22 | 5 | 466 | 1 |
| | Bradi1g53650 | BdAP2-6 | 1 | 438 | 7 |
| | Bradi1g03880 | BdAP2-1 | 1 | 436 | 0 |
| | Bradi1g30337 | BdAP2-3 | 1 | 378 | 0 |
| | Bradi2g53070 | BdAP2-14 | 2 | 376 | 0 |
| | Bradi3g59300 | BdAP2-18 | 3 | 372 | 8 |
| | Bradi3g15880 | BdAP2-16 | 3 | 287 | 2 |
| | Bradi2g48130 | BdAP2-13 | 2 | 392 | 0 |
| | Bradi1g51045 | BdAP2-5 | 1 | 413 | 0 |
| | Bradi2g18570 | BdAP2-10 | 2 | 458 | 0 |
| | Bradi4g30617 | BdAP2-19 | 4 | 393 | 0 |
| | Bradi2g26987 | BdAP2-11 | 2 | 413 | 0 |
| | Bradi4g43877 | BdAP2-20 | 4 | 420 | 3 |
| | Bradi1g57560 | BdAP2-7 | 1 | 604 | 0 |
| | Bradi1g07290 | BdAP2-2 | 1 | 547 | 0 |
| | Bradi1g72890 | BdAP2-9 | 1 | 528 | 4 |
| | Bradi5g24360 | BdAP2-23 | 5 | 491 | 0 |
| | Bradi2g57747 | BdAP2-15 | 2 | 684 | 0 |
| | Bradi1g64240 | BdAP2-8 | 1 | 394 | 0 |
| | Bradi5g14960 | BdAP2-21 | 5 | 686 | 0 |
| | Bradi1g31337 | BdAP2-4 | 1 | 466 | 0 |
| | Bradi3g48697 | BdAP2-17 | 3 | 689 | 0 |
| DREB | Bradi2g60340 | BdDREB-23 | 2 | 240 | 2 |
| | Bradi2g60331 | BdDREB-22 | 2 | 243 | 2 |
| | Bradi3g57360 | BdDREB-41 | 3 | 236 | 0 |
| | Bradi4g35610 | BdDREB-51 | 4 | 244 | 0 |
| | Bradi4g35570 | BdDREB-47 | 4 | 244 | 0 |
| | Bradi4g35580 | BdDREB-48 | 4 | 258 | 0 |
| | Bradi4g35600 | BdDREB-50 | 4 | 248 | 0 |
| | Bradi4g35620 | BdDREB-52 | 4 | 251 | 0 |
| | Bradi4g35590 | BdDREB-49 | 4 | 250 | 0 |
| | Bradi1g57970 | BdDREB-9 | 1 | 242 | 0 |
| | Bradi1g77120 | BdDREB-13 | 1 | 256 | 0 |
| | Bradi4g35650 | BdDREB-54 | 4 | 238 | 7 |
| | Bradi4g35630 | BdDREB-53 | 4 | 254 | 1 |
| | Bradi3g51630 | BdDREB-38 | 3 | 227 | 1 |
| | Bradi1g49570 | BdDREB-8 | 1 | 224 | 0 |
| | Bradi1g49560 | BdDREB-7 | 1 | 220 | 3 |
| | Bradi3g50620 | BdDREB-34 | 3 | 262 | 0 |
| | Bradi5g17610 | BdDREB-57 | 5 | 295 | 0 |

| | | | | | |
|---|---|---|---|---|---|
| | Bradi5g17630 | BdDREB-59 | 5 | 208 | 0 |
| | Bradi3g50625 | BdDREB-35 | 3 | 235 | 0 |
| | Bradi5g17640 | BdDREB-60 | 5 | 211 | 0 |
| | Bradi3g50630 | BdDREB-36 | 3 | 237 | 0 |
| | Bradi5g18850 | BdDREB-61 | 5 | 197 | 0 |
| | Bradi3g51610 | BdDREB-37 | 3 | 285 | 0 |
| | Bradi3g31600 | BdDREB-28 | 3 | 191 | 0 |
| | Bradi5g17620 | BdDREB-58 | 5 | 251 | 0 |
| | Bradi3g33355 | BdDREB-29 | 3 | 263 | 0 |
| | Bradi3g08790 | BdDREB-25 | 3 | 249 | 0 |
| | Bradi1g38110 | BdDREB-2 | 1 | 264 | 0 |
| | Bradi2g06180 | BdDREB-15 | 2 | 251 | 0 |
| | Bradi3g45997 | BdDREB-32 | 3 | 171 | 0 |
| | Bradi4g21265 | BdDREB-45 | 4 | 192 | 0 |
| | Bradi5g11295 | BdDREB-55 | 5 | 192 | 0 |
| | Bradi2g25050 | BdDREB-18 | 2 | 179 | 0 |
| | Bradi2g57201 | BdDREB-21 | 2 | 210 | 2 |
| | Bradi3g37544 | BdDREB-31 | 3 | 214 | 0 |
| | Bradi3g60018 | BdDREB-44 | 3 | 216 | 0 |
| | Bradi3g56801 | BdDREB-40 | 3 | 223 | 0 |
| | Bradi5g24700 | BdDREB-63 | 5 | 142 | 0 |
| | Bradi1g67350 | BdDREB-10 | 1 | 246 | 0 |
| | Bradi3g57862 | BdDREB-42 | 3 | 232 | 1 |
| | Bradi1g46120 | BdDREB-4 | 1 | 235 | 0 |
| | Bradi1g48320 | BdDREB-6 | 1 | 176 | 0 |
| | Bradi5g24110 | BdDREB-62 | 5 | 229 | 5 |
| | Bradi5g24710 | BdDREB-64 | 5 | 162 | 0 |
| | Bradi3g54160 | BdDREB-39 | 3 | 168 | 0 |
| | Bradi1g47480 | BdDREB-5 | 1 | 153 | 0 |
| | Bradi5g24720 | BdDREB-65 | 5 | 143 | 0 |
| | Bradi1g36590 | BdDREB-1 | 1 | 225 | 0 |
| | Bradi3g07450 | BdDREB-24 | 3 | 211 | 20 |
| | Bradi2g29010 | BdDREB-19 | 2 | 229 | 1 |
| | Bradi1g72990 | BdDREB-12 | 1 | 332 | 0 |
| | Bradi3g12680 | BdDREB-26 | 3 | 213 | 0 |
| | Bradi2g04000 | BdDREB-14 | 2 | 255 | 1 |
| | Bradi2g29960 | BdDREB-20 | 2 | 381 | 13 |
| | Bradi2g15847 | BdDREB-16 | 2 | 284 | 0 |
| | Bradi5g16450 | BdDREB-56 | 5 | 487 | 0 |
| | Bradi3g49810 | BdDREB-33 | 3 | 470 | 1 |
| | Bradi3g35560 | BdDREB-30 | 3 | 274 | 7 |
| | Bradi4g29010 | BdDREB-46 | 4 | 282 | 2 |
| | Bradi1g71740 | BdDREB-11 | 1 | 287 | 1 |
| | Bradi3g24000 | BdDREB-27 | 3 | 228 | 0 |
| | Bradi3g58980 | BdDREB-43 | 3 | 315 | 8 |
| | Bradi1g45470 | BdDREB-3 | 1 | 351 | 0 |
| | Bradi2g22505 | BdDREB-17 | 2 | 257 | 0 |
| ERF | Bradi1g53756 | BdERF-11 | 1 | 289 | 0 |

| | | | | |
|---|---|---|---|---|
| Bradi1g53753 | BdERF-10 | 1 | 337 | 0 |
| Bradi1g23756 | BdERF-5 | 1 | 293 | 0 |
| Bradi1g54450 | BdERF-12 | 1 | 256 | 0 |
| Bradi1g38238 | BdERF-7 | 1 | 288 | 0 |
| Bradi4g03371 | BdERF-46 | 4 | 420 | 0 |
| Bradi4g35655 | BdERF-49 | 4 | 227 | 0 |
| Bradi2g24175 | BdERF-21 | 2 | 345 | 0 |
| Bradi2g56145 | BdERF-28 | 2 | 377 | 3 |
| Bradi4g41616 | BdERF-51 | 4 | 353 | 0 |
| Bradi5g08380 | BdERF-52 | 5 | 285 | 3 |
| Bradi3g58015 | BdERF-44 | 3 | 248 | 0 |
| Bradi3g38140 | BdERF-38 | 3 | 279 | 1 |
| Bradi4g27850 | BdERF-47 | 4 | 314 | 3 |
| Bradi3g40343 | BdERF-39 | 3 | 212 | 0 |
| Bradi2g02100 | BdERF-15 | 2 | 336 | 5 |
| Bradi3g60120 | BdERF-45 | 3 | 394 | 7 |
| Bradi4g31040 | BdERF-48 | 4 | 401 | 10 |
| Bradi1g46690 | BdERF-8 | 1 | 351 | 23 |
| Bradi1g72457 | BdERF-13 | 1 | 307 | 0 |
| Bradi2g27920 | BdERF-22 | 2 | 168 | 4 |
| Bradi2g11890 | BdERF-17 | 2 | 197 | 3 |
| Bradi1g17961 | BdERF-3 | 1 | 219 | 0 |
| Bradi5g17480 | BdERF-54 | 5 | 289 | 0 |
| Bradi3g50490 | BdERF-42 | 3 | 296 | 5 |
| Bradi3g15084 | BdERF-34 | 3 | 228 | 0 |
| Bradi1g00666 | BdERF-1 | 1 | 284 | 0 |
| Bradi3g12566 | BdERF-32 | 3 | 294 | 0 |
| Bradi3g12567 | BdERF-33 | 3 | 172 | 0 |
| Bradi4g38941 | BdERF-50 | 4 | 129 | 0 |
| Bradi3g12565 | BdERF-31 | 3 | 123 | 0 |
| Bradi3g27690 | BdERF-37 | 3 | 121 | 0 |
| Bradi1g75040 | BdERF-14 | 1 | 131 | 0 |
| Bradi3g50508 | BdERF-43 | 3 | 335 | 0 |
| Bradi5g17490 | BdERF-55 | 5 | 364 | 0 |
| Bradi5g25570 | BdERF-57 | 5 | 178 | 1 |
| Bradi2g16442 | BdERF-18 | 2 | 295 | 0 |
| Bradi2g07357 | BdERF-16 | 2 | 359 | 0 |
| Bradi2g45530 | BdERF-24 | 2 | 300 | 0 |
| Bradi1g48625 | BdERF-9 | 1 | 289 | 0 |
| Bradi2g31480 | BdERF-23 | 2 | 271 | 1 |
| Bradi2g50035 | BdERF-25 | 2 | 191 | 0 |
| Bradi1g04110 | BdERF-2 | 1 | 301 | 15 |
| Bradi3g47610 | BdERF-41 | 3 | 351 | 0 |
| Bradi2g21060 | BdERF-19 | 2 | 236 | 1 |
| Bradi2g52370 | BdERF-26 | 2 | 243 | 6 |
| Bradi3g06562 | BdERF-30 | 3 | 202 | 0 |
| Bradi5g21250 | BdERF-56 | 5 | 254 | 8 |
| Bradi3g18070 | BdERF-35 | 3 | 170 | 1 |

| | | | | |
|---|---|---|---|---|
| Bradi3g18073 | BdERF-36 | 3 | 202 | 0 |
| Bradi5g08471 | BdERF-53 | 5 | 260 | 0 |
| Bradi3g44470 | BdERF-40 | 3 | 248 | 0 |
| Bradi1g18580 | BdERF-4 | 1 | 319 | 0 |
| Bradi1g33550 | BdERF-6 | 1 | 188 | 0 |
| Bradi3g04410 | BdERF-29 | 3 | 193 | 1 |
| Bradi2g21067 | BdERF-20 | 2 | 195 | 0 |
| Bradi2g52380 | BdERF-27 | 2 | 174 | 0 |
| Bradi3g04370 | | | | |
| Bradi3g36820 | | | | |
| Bradi1g69208 | | | | |
| Bradi3g33670 | | | | |
| Bradi1g69214 | | | | |
| Bradi3g42627 | | | | |
| Bradi3g43822 | | | | |
| Bradi1g35410 | | | | |
| Bradi1g35420 | | | | |
| Bradi1g35400 | | | | |
| Bradi3g15591 | | | | |
| Bradi1g18870 | | | | |
| Bradi3g04381 | | | | |
| Bradi3g04375 | | | | |
| Bradi2g09434 | | | | |
| Bradi3g41543 | | | | |
| Bradi3g41547 | | | | |

Note: The gene models in red were excluded from further analysis because their AP2 domains were too short or divergent to fit into an acceptable phylogeny.

Supplementary Table 2: The comparison of BdDREB/CBFs among three papers.

| genes identified by us | subgroups | genes identified by Li et al[54] | subgroups classified by us | genes identified by Ryu et al[55] | subgroupsclassified by us |
|---|---|---|---|---|---|
| Bradi2g60340.1 | A1 | Bradi4g35630.1 | A1 | Bradi3g51630.1 | A1 |
| Bradi2g60331.1 | A1 | Bradi1g77120.1 | A1 | Bradi1g49560.1 | A1 |
| Bradi3g57360.1 | A1 | Bradi4g35580.1 | A1 | Bradi1g49570.1 | A1 |
| Bradi4g35610.1 | A1 | Bradi4g35570.1 | A1 | Bradi4g35650.1 | A1 |
| Bradi4g35570.1 | A1 | Bradi3g57360.1 | A1 | Bradi4g35600.1 | A1 |
| Bradi4g35580.1 | A1 | Bradi1g57970.1 | A1 | Bradi1g57970.1 | A1 |
| Bradi4g35600.1 | A1 | Bradi2g60331.1 | A1 | Bradi4g35590.1 | A1 |
| Bradi4g35620.1 | A1 | Bradi2g60340.1 | A1 | Bradi4g35610.1 | A1 |
| Bradi4g35590.1 | A1 | Bradi4g35600.1 | A1 | Bradi4g35620.1 | A1 |
| Bradi1g57970.1 | A1 | Bradi4g35620.1 | A1 | Bradi4g35630.1 | A1 |
| Bradi1g77120.1 | A1 | Bradi4g35610.1 | A1 | Bradi4g35570.1 | A1 |
| Bradi4g35650.1 | A1 | Bradi4g35590.1 | A1 | Bradi3g57360.1 | A1 |
| Bradi4g35630.1 | A1 | Bradi4g35650.1 | A1 | Bradi2g60340.1 | A1 |
| Bradi3g51630.1 | A1 | Bradi3g51630.1 | A1 | Bradi4g35580.1 | A1 |
| Bradi1g49570.1 | A1 | Bradi3g45997.1 | A4 | Bradi1g77120.1 | A1 |
| Bradi1g49560.1 | A1 | Bradi3g45997.1 | A4 | Bradi3g50630.1 | A1 |
| Bradi3g50620.1 | A1 | Bradi4g21265.1 | A4 | Bradi5g17610.1 | A1 |
| Bradi5g17610.1 | A1 | Bradi4g35640.1 | without AP2 domain | Bradi5g17640.1 | A1 |
| Bradi5g17630.1 | A1 | | | Bradi5g17630.1 | A1 |
| Bradi3g50625.1 | A1 | | | | |
| Bradi5g17640.1 | A1 | | | | |
| Bradi3g50630.1 | A1 | | | | |
| Bradi2g29010.1 | A2 | | | | |
| Bradi1g72990.1 | A2 | | | | |
| Bradi3g12680.1 | A2 | | | | |
| Bradi2g04000.2 | A2 | | | | |
| Bradi2g29960.1 | A2 | | | | |

| | |
|---|---|
| Bradi5g24710.1 | A3 |
| Bradi3g54160.1 | A3 |
| Bradi1g47480.1 | A3 |
| Bradi5g24720.1 | A3 |
| Bradi1g36590.1 | A3 |
| Bradi3g07450.1 | A3 |
| Bradi5g18850.1 | A4 |
| Bradi3g51610.1 | A4 |
| Bradi3g31600.1 | A4 |
| Bradi5g17620.1 | A4 |
| Bradi3g33355.1 | A4 |
| Bradi3g08790.1 | A4 |
| Bradi1g38110.1 | A4 |
| Bradi2g06180.1 | A4 |
| Bradi3g45997.1 | A4 |
| Bradi4g21265.1 | A4 |
| Bradi5g11295.1 | A4 |
| Bradi2g25050.1 | A5 |
| Bradi2g57201.1 | A5 |
| Bradi3g37544.1 | A5 |
| Bradi3g60018.1 | A5 |
| Bradi3g56801.1 | A5 |
| Bradi5g24700.1 | A5 |
| Bradi1g67350.1 | A5 |
| Bradi3g57862.1 | A5 |
| Bradi1g46120.1 | A5 |
| Bradi1g48320.1 | A5 |
| Bradi5g24110.1 | A5 |
| Bradi2g15847.1 | A6 |
| Bradi5g16450.1 | A6 |
| Bradi3g49810.1 | A6 |
| Bradi3g35560.1 | A6 |

| | |
|---|---|
| Bradi4g29010.1 | A6 |
| Bradi1g71740.1 | A6 |
| Bradi3g24000.1 | A6 |
| Bradi3g58980.1 | A6 |
| Bradi1g45470.1 | A6 |
| Bradi2g22505.1 | A6 |

Note: The genes with red color represent the genes that were found in two papers but not found in three papers.The gene with blue color represents the gene that was only found in the paper written by Li et al[54]. The genes with orange backgroud represent the genes that were found in three papers.

Supplementary Table 3:

Summary of the AP2/EREBP family of *Arabidopsis*, sorghum, rice, wheat and *B. distachyon*.

| Plant classification | | *A.thaliana* [28] | sorghum[59] | rice[7] | wheat [60] | *B. distachyon* |
|---|---|---|---|---|---|---|
| subfamily | Group | Number | Number | Number | Number | Number |
| DREB subfamily | A1 | 6 | 8 | 10 | 39 | 22 |
| | A2 | 8 | 11 | 6 | 5 | 5 |
| | A3 | 16 | 5 | 5 | 0 | 6 |
| | A4 | 16 | 2 | 12 | 1 | 11 |
| | A5 | 16 | 20 | 15 | 4 | 11 |
| | A6 | 10 | 6 | 9 | 8 | 10 |
| | Total | 57 | 52 | 57 | 57 | 65 |
| ERF subfamily | Bl | 15 | 8 | 18 | 7 | 16 |
| | B2 | 5 | 9 | 12 | 19 | 7 |
| | B3 | 18 | 5 | 19 | 8 | 14 |
| | B4 | 7 | 14 | 12 | 4 | 6 |
| | B5 | 8 | 6 | 4 | 0 | 4 |
| | B6 | 12 | 11 | 3 | 9 | 7 |
| | B7 | 0 | 0 | 9 | 0 | 3 |
| | Total | 65 | 53 | 77 | 47 | 57 |
| AP2 subfamily | | 18 | 16 | 24 | 9 | 23 |
| RAV subfamily | | 6 | 4 | 5 | 3 | 4 |
| Soloist | | 1 | 1 | 0 | 1 | 1 |
| Total | | 147 | 126 | 163 | 117 | 150 |

Supplementary Table 4: The genes derived from gene duplication event.

a:The genes derived from tandom gene duplication event.

| gene cluster No. | Pair | Gene 1 | Gene 1 subfamily | Gene 2 | Gene 2 subfamily | Identity (%) |
|---|---|---|---|---|---|---|
| 1 | 1 | Bradi4g35600.1 | DREB | Bradi4g35610.1 | DREB | 75 |
| | 2 | Bradi4g35600.1 | DREB | Bradi4g35590.1 | DREB | 74 |
| | 3 | Bradi4g35600.1 | DREB | Bradi4g35620.1 | DREB | 76 |
| | 4 | Bradi4g35600.1 | DREB | Bradi4g35570.1 | DREB | 70 |
| | 5 | Bradi4g35570.1 | DREB | Bradi4g35580.1 | DREB | 86 |
| 2 | 6 | Bradi2g60340.1 | DREB | Bradi2g60331.1 | DREB | 94 |
| 3 | 7 | Bradi1g49570.1 | DREB | Bradi1g49560.1 | DREB | 78 |
| 4 | 8 | Bradi5g24720.1 | DREB | Bradi5g24710.1 | DREB | 71 |

b:The genes derived from segmental gene duplication event.

| Gene 1 | Gene 1 subfamily | Gene 2 | Gene 2 subfamily | Identity (%) |
|---|---|---|---|---|
| Bradi1g36590.1 | DREB | Bradi3g07450.1 | DREB | 78 |
| Bradi1g47480.1 | DREB | Bradi3g54160.1 | DREB | 73 |
| Bradi3g12565.1 | ERF | Bradi4g38941.1 | ERF | 74 |
| Bradi2g21060.1 | ERF | Bradi2g52370.1 | ERF | 72 |

Supplementary Table 5:

The expression profiles ($Log_2$-based RPKM value) of BdAP2/EREBP in different tissues.

| subfamil | gene | Root | Stem | Leaf | Root_VS_Stem q value | Root_VS_Leaf q value | Stem_VS_Leaf q value |
|---|---|---|---|---|---|---|---|
| RAV | Bradi2g17610 | 6.07 | -0.02 | 2.16 | 0.06 | 0.05 | 0.22 |
| | Bradi2g47220 | 8.46 | 3.89 | 2.98 | 0.04 | 0.07 | 0.88 |
| | Bradi2g02720 | 3.22 | -19.93 | -2.45 | 0.00 | 0.08 | 1.00 |
| | Bradi2g02710 | 1.57 | -2.24 | -19.93 | 0.30 | 0.01 | 1.00 |
| AP2 | Bradi2g37800 | 2.74 | 2.23 | 5.66 | 0.34 | 0.50 | 0.10 |
| | Bradi5g24100 | 1.11 | 1.09 | 1.84 | 0.92 | 0.63 | 0.66 |
| | Bradi1g53650 | 4.89 | 2.23 | 3.19 | 0.18 | 0.43 | 0.92 |
| | Bradi1g03880 | 4.20 | 1.85 | 2.17 | 0.13 | 0.14 | 0.75 |
| | Bradi1g30337 | -19.93 | -2.96 | -19.93 | 1.00 | 1.00 | 1.00 |
| | Bradi2g53070 | -19.93 | -19.93 | -19.93 | 1.00 | 1.00 | 1.00 |
| | Bradi3g59300 | 1.63 | 1.07 | 2.34 | 0.77 | 0.97 | 0.83 |
| | Bradi3g15880 | 2.96 | 2.38 | 3.34 | 0.65 | 0.64 | 0.95 |
| | Bradi2g48130 | -3.47 | -2.40 | 0.39 | 1.00 | 0.44 | 0.58 |
| | Bradi1g51045 | -19.93 | -19.93 | -19.93 | 1.00 | 1.00 | 1.00 |
| | Bradi2g18570 | -1.38 | -19.93 | -19.93 | 1.00 | 1.00 | 1.00 |
| | Bradi4g30617 | -19.93 | -0.50 | -1.82 | 1.00 | 1.00 | 1.00 |
| | Bradi2g26987 | 0.96 | 2.34 | 2.17 | 0.54 | 0.68 | 0.45 |
| | Bradi4g43877 | 0.94 | 0.66 | -1.97 | 0.94 | 0.17 | 0.12 |
| | Bradi1g57560 | -19.93 | -1.72 | -19.93 | 1.00 | 1.00 | 1.00 |
| | Bradi1g07290 | -19.93 | -2.14 | -19.93 | 1.00 | 1.00 | 1.00 |
| | Bradi1g72890 | -1.53 | -19.93 | -19.93 | 1.00 | 1.00 | 1.00 |
| | Bradi5g24360 | 1.67 | -3.94 | -19.93 | 0.25 | 0.00 | 1.00 |
| | Bradi2g57747 | -1.17 | -19.93 | -19.93 | 1.00 | 1.00 | 1.00 |
| | Bradi1g64240 | -1.64 | -19.93 | -19.93 | 1.00 | 1.00 | 1.00 |
| | Bradi5g14960 | 0.25 | -19.93 | -19.93 | 1.00 | 1.00 | 1.00 |
| | Bradi1g31337 | -0.69 | -19.93 | -19.93 | 0.12 | 1.00 | 1.00 |
| | Bradi3g48697 | -3.59 | -19.93 | -19.93 | 1.00 | 1.00 | 1.00 |
| DREB | Bradi2g60340 | -19.93 | 2.16 | 3.27 | 0.01 | 0.00 | 0.60 |
| | Bradi2g60331 | 1.25 | 1.54 | 3.13 | 1.00 | 0.24 | 0.58 |
| | Bradi3g57360 | -19.93 | -19.93 | -19.93 | 1.00 | 1.00 | 1.00 |
| | Bradi4g35610 | -19.93 | -19.93 | -19.93 | 1.00 | 1.00 | 1.00 |
| | Bradi4g35570 | -1.98 | -19.93 | -19.93 | 1.00 | 1.00 | 1.00 |
| | Bradi4g35580 | -19.93 | -19.93 | -19.93 | 1.00 | 1.00 | 1.00 |
| | Bradi4g35600 | -19.93 | -19.93 | -19.93 | 1.00 | 1.00 | 1.00 |
| | Bradi4g35620 | -19.93 | 1.56 | -19.93 | 1.00 | 1.00 | 1.00 |
| | Bradi4g35590 | -19.93 | -19.93 | -19.93 | 1.00 | 1.00 | 1.00 |
| | Bradi1g57970 | -19.93 | -19.93 | -19.93 | 1.00 | 1.00 | 1.00 |
| | Bradi1g77120 | 0.78 | 3.59 | -0.32 | 0.25 | 1.00 | 0.14 |
| | Bradi4g35650 | 9.20 | 5.94 | 4.27 | 0.09 | 0.10 | 0.48 |
| | Bradi4g35630 | 4.29 | 1.37 | -19.93 | 0.10 | 0.01 | 1.00 |
| | Bradi3g51630 | 6.67 | 4.62 | 2.61 | 0.36 | 0.06 | 0.21 |
| | Bradi1g49570 | 5.77 | -0.68 | -19.93 | 0.02 | 0.00 | 1.00 |
| | Bradi1g49560 | 7.33 | 1.13 | -19.93 | 0.05 | 0.00 | 0.02 |
| | Bradi3g50620 | 1.37 | -19.93 | -19.93 | 1.00 | 1.00 | 1.00 |

| | | | | | | |
|---|---|---|---|---|---|---|
| Bradi5g17610 | 4.18 | 4.32 | -0.59 | 0.97 | 0.04 | 0.19 |
| Bradi5g17630 | -19.93 | -19.93 | -19.93 | 1.00 | 1.00 | 1.00 |
| Bradi3g50625 | 3.49 | -19.93 | -19.93 | 0.02 | 0.01 | 1.00 |
| Bradi5g17640 | 4.92 | 2.08 | 1.49 | 0.18 | 0.10 | 0.79 |
| Bradi3g50630 | 3.78 | 3.15 | 2.26 | 0.78 | 0.46 | 0.63 |
| Bradi5g18850 | -19.93 | 0.64 | -19.93 | 1.00 | 1.00 | 1.00 |
| Bradi3g51610 | 7.24 | 2.16 | -3.31 | 0.02 | 0.21 | 0.19 |
| Bradi3g31600 | -19.93 | 0.10 | -19.93 | 1.00 | 1.00 | 1.00 |
| Bradi5g17620 | 2.14 | -19.93 | -19.93 | 0.01 | 0.01 | 1.00 |
| Bradi3g33355 | 2.93 | 4.78 | 1.91 | 0.36 | 1.00 | 0.38 |
| Bradi3g08790 | -19.93 | -19.93 | -19.93 | 1.00 | 1.00 | 1.00 |
| Bradi1g38110 | -19.93 | -19.93 | -2.47 | 1.00 | 1.00 | 1.00 |
| Bradi2g06180 | -19.93 | -19.93 | -19.93 | 1.00 | 1.00 | 1.00 |
| Bradi3g45997 | -19.93 | -19.93 | -19.93 | 1.00 | 1.00 | 1.00 |
| Bradi4g21265 | 3.71 | -19.93 | -19.93 | 0.03 | 0.02 | 1.00 |
| Bradi5g11295 | -19.93 | -1.71 | -19.93 | 1.00 | 1.00 | 1.00 |
| Bradi2g25050 | -19.93 | -19.93 | -19.93 | 1.00 | 1.00 | 1.00 |
| Bradi2g57201 | 11.13 | 3.61 | -19.93 | 0.05 | 0.00 | 0.02 |
| Bradi3g37544 | 6.19 | -19.93 | -19.93 | 0.00 | 0.00 | 1.00 |
| Bradi3g60018 | 1.83 | -19.93 | 0.05 | 1.00 | 1.00 | 1.00 |
| Bradi3g56801 | 4.54 | 1.62 | -19.93 | 0.10 | 0.01 | 1.00 |
| Bradi5g24700 | -19.93 | -19.93 | -19.93 | 1.00 | 1.00 | 1.00 |
| Bradi1g67350 | 2.85 | 4.26 | -19.93 | 0.58 | 0.01 | 0.00 |
| Bradi3g57862 | 4.74 | 2.13 | -2.30 | 0.30 | 0.02 | 0.19 |
| Bradi1g46120 | 6.77 | -19.93 | -19.93 | 0.01 | 0.00 | 1.00 |
| Bradi1g48320 | 6.63 | 1.11 | -1.08 | 0.10 | 0.20 | 0.20 |
| Bradi5g24110 | 5.32 | 3.00 | -0.24 | 0.23 | 0.49 | 0.50 |
| Bradi5g24710 | -19.93 | -19.93 | -19.93 | 1.00 | 1.00 | 1.00 |
| Bradi3g54160 | 0.83 | -19.93 | -19.93 | 1.00 | 1.00 | 1.00 |
| Bradi1g47480 | -2.24 | 0.35 | -19.93 | 0.33 | 1.00 | 0.02 |
| Bradi5g24720 | -19.93 | -19.93 | -19.93 | 1.00 | 1.00 | 1.00 |
| Bradi1g36590 | -19.93 | 1.51 | -19.93 | 0.01 | 1.00 | 0.01 |
| Bradi3g07450 | -19.93 | 2.40 | -2.77 | 0.00 | 1.00 | 0.25 |
| Bradi2g29010 | -19.93 | -19.93 | -19.93 | 1.00 | 1.00 | 1.00 |
| Bradi1g72990 | -19.93 | -19.93 | -19.93 | 1.00 | 1.00 | 1.00 |
| Bradi3g12680 | -19.93 | -19.93 | -19.93 | 1.00 | 1.00 | 1.00 |
| Bradi2g04000 | 1.65 | -0.49 | -3.59 | 0.30 | 0.26 | 0.32 |
| Bradi2g29960 | 6.78 | 4.91 | 4.83 | 0.26 | 0.24 | 0.53 |
| Bradi2g15847 | 2.52 | -0.72 | 0.59 | 0.33 | 0.60 | 1.00 |
| Bradi5g16450 | 0.07 | -2.89 | -1.65 | 1.00 | 1.00 | 1.00 |
| Bradi3g49810 | 3.71 | 2.58 | -0.14 | 0.66 | 0.12 | 0.17 |
| Bradi3g35560 | 7.47 | 7.99 | 7.47 | 0.53 | 0.41 | 0.69 |
| Bradi4g29010 | 6.08 | 5.78 | 5.72 | 0.88 | 0.62 | 0.86 |
| Bradi1g71740 | 4.05 | 2.97 | 0.70 | 0.63 | 0.09 | 0.14 |
| Bradi3g24000 | 0.90 | 0.83 | 0.42 | 1.00 | 0.26 | 0.34 |
| Bradi3g58980 | 8.89 | 5.64 | 7.87 | 0.09 | 0.44 | 0.42 |
| Bradi1g45470 | 3.26 | 0.65 | 2.24 | 0.30 | 0.50 | 0.49 |
| Bradi2g22505 | -19.93 | -1.60 | -19.93 | 1.00 | 1.00 | 1.00 |

| | | | | | | | |
|---|---|---|---|---|---|---|---|
| ERF | Bradi1g53756 | -19.93 | -19.93 | -19.93 | 1.00 | 1.00 | 1.00 |
| | Bradi1g53753 | -19.93 | -19.93 | -19.93 | 1.00 | 1.00 | 1.00 |
| | Bradi1g23756 | -19.93 | -0.41 | -2.02 | 1.00 | 1.00 | 1.00 |
| | Bradi1g54450 | -1.86 | -19.93 | -19.93 | 1.00 | 1.00 | 1.00 |
| | Bradi1g38238 | -19.93 | -19.93 | -19.93 | 1.00 | 1.00 | 1.00 |
| | Bradi4g03371 | 0.00 | -19.93 | -2.32 | 0.01 | 0.14 | 1.00 |
| | Bradi4g35655 | -19.93 | -19.93 | -19.93 | 1.00 | 1.00 | 1.00 |
| | Bradi2g24175 | 5.11 | -19.93 | -19.93 | 0.00 | 0.00 | 1.00 |
| | Bradi2g56145 | 3.49 | -19.93 | -1.45 | 0.00 | 0.07 | 1.00 |
| | Bradi4g41616 | 3.92 | -19.93 | -0.17 | 0.01 | 0.11 | 1.00 |
| | Bradi5g08380 | -1.79 | -5.56 | -19.93 | 1.00 | 1.00 | 1.00 |
| | Bradi3g58015 | 10.93 | 3.32 | -19.93 | 0.04 | 0.08 | 0.01 |
| | Bradi3g38140 | 9.46 | 3.33 | 0.40 | 0.02 | 0.21 | 0.29 |
| | Bradi4g27850 | 0.42 | -0.59 | 1.36 | 0.61 | 0.89 | 0.54 |
| | Bradi3g40343 | -19.93 | -19.93 | -19.93 | 1.00 | 1.00 | 1.00 |
| | Bradi2g02100 | 4.69 | 4.50 | 2.63 | 0.98 | 0.90 | 0.97 |
| | Bradi3g60120 | 6.39 | 3.62 | 4.12 | 0.13 | 0.24 | 0.96 |
| | Bradi4g31040 | 6.32 | 4.69 | 4.75 | 0.50 | 0.25 | 0.52 |
| | Bradi1g46690 | 6.44 | 4.65 | 3.11 | 0.46 | 0.16 | 0.33 |
| | Bradi1g72457 | 7.64 | 3.67 | -0.25 | 0.41 | 0.33 | 0.68 |
| | Bradi2g27920 | 0.73 | -1.13 | -19.93 | 0.14 | 0.01 | 1.00 |
| | Bradi2g11890 | 4.90 | 0.43 | 0.60 | 0.12 | 0.06 | 0.80 |
| | Bradi1g17961 | 5.12 | -19.93 | -19.93 | 0.00 | 0.00 | 1.00 |
| | Bradi5g17480 | 5.11 | 2.62 | 1.44 | 0.26 | 0.42 | 0.52 |
| | Bradi3g50490 | 6.22 | 4.85 | 2.49 | 0.37 | 0.07 | 0.18 |
| | Bradi3g15084 | 3.34 | -19.93 | -19.93 | 0.02 | 0.02 | 1.00 |
| | Bradi1g00666 | 5.18 | 0.10 | -1.87 | 0.09 | 0.02 | 1.00 |
| | Bradi3g12566 | -19.93 | -19.93 | -19.93 | 1.00 | 1.00 | 1.00 |
| | Bradi3g12567 | -19.93 | -19.93 | -19.93 | 1.00 | 1.00 | 1.00 |
| | Bradi4g38941 | -2.00 | -19.93 | -19.93 | 1.00 | 1.00 | 1.00 |
| | Bradi3g12565 | -19.93 | -19.93 | -19.93 | 1.00 | 1.00 | 1.00 |
| | Bradi3g27690 | -19.93 | -19.93 | -19.93 | 1.00 | 1.00 | 1.00 |
| | Bradi1g75040 | -19.93 | -19.93 | -19.93 | 1.00 | 1.00 | 1.00 |
| | Bradi3g50508 | 2.51 | -19.93 | -19.93 | 0.01 | 0.01 | 1.00 |
| | Bradi5g17490 | 2.55 | -19.93 | -19.93 | 0.02 | 0.01 | 1.00 |
| | Bradi5g25570 | 4.87 | 2.34 | 2.86 | 0.34 | 0.10 | 0.58 |
| | Bradi2g16442 | 0.95 | -19.93 | -19.93 | 0.02 | 0.01 | 1.00 |
| | Bradi2g07357 | 0.12 | -0.12 | -4.60 | 1.00 | 1.00 | 1.00 |
| | Bradi2g45530 | 1.37 | 2.56 | -19.93 | 0.40 | 1.00 | 0.00 |
| | Bradi1g48625 | -19.93 | -19.93 | -19.93 | 1.00 | 1.00 | 1.00 |
| | Bradi2g31480 | 1.33 | 1.49 | -0.81 | 0.94 | 0.72 | 0.53 |
| | Bradi2g50035 | 2.98 | -19.93 | -19.93 | 1.00 | 1.00 | 1.00 |
| | Bradi1g04110 | 6.85 | 6.87 | 7.78 | 0.94 | 0.68 | 0.77 |
| | Bradi3g47610 | -19.93 | -19.93 | -19.93 | 1.00 | 1.00 | 1.00 |
| | Bradi2g21060 | 9.18 | 6.46 | 3.71 | 0.17 | 0.11 | 0.78 |
| | Bradi2g52370 | 7.83 | 3.34 | 4.23 | 0.07 | 0.06 | 0.77 |
| | Bradi3g06562 | 2.91 | 1.89 | 3.47 | 0.56 | 0.80 | 0.40 |
| | Bradi5g21250 | 7.73 | 4.63 | 2.37 | 0.15 | 0.09 | 0.46 |

| | | | | | | |
|---|---|---|---|---|---|---|
| Bradi3g18070 | 6.75 | 0.76 | -0.11 | 0.04 | 0.08 | 1.00 |
| Bradi3g18073 | 1.52 | 0.46 | -19.93 | 1.00 | 1.00 | 1.00 |
| Bradi5g08471 | -0.47 | -19.93 | -19.93 | 1.00 | 1.00 | 1.00 |
| Bradi3g44470 | -19.93 | -19.93 | -19.93 | 1.00 | 1.00 | 1.00 |
| Bradi1g18580 | -19.93 | -19.93 | -19.93 | 1.00 | 1.00 | 1.00 |
| Bradi1g33550 | 4.02 | 2.49 | -0.25 | 0.22 | 0.30 | 1.00 |
| Bradi3g04410 | 2.79 | 2.89 | 3.27 | 0.97 | 0.57 | 0.58 |
| Bradi2g21067 | 2.59 | 2.27 | -3.50 | 0.81 | 0.27 | 0.34 |
| Bradi2g52380 | 1.60 | -19.93 | -19.93 | 1.00 | 1.00 | 1.00 |

Note: if the q value was less than 0.05, it was marked in red.

Supplementary Table 6: The expression profiles ($Log_2$-based fold change and the corresponding q value ) of BdAP2/EREBP under four abiotic stress conditions.

| | | $Log_2$ based fold change of treatment/control at each time point under the corresponding treatment | | | | | | | | | | | | | | | | | | | |
|---|---|---|---|---|---|---|---|---|---|---|---|---|---|---|---|---|---|---|---|---|---|
| sub_ family | gene | cold 1h | cold 2h | cold 5h | cold 10h | cold 24 | heat 1h | heat 2h | heat 5h | heat 10 | heat 24 | drought | drought | drought 5 | drought 1 | drought 2 | salt 1h | salt 2h | salt 5h | salt 10h | salt 24h |
| RAV | Bradi2g17610 | -0.23 | -0.24 | -0.45 | -0.98 | -0.10 | 0.66 | 0.02 | -0.28 | -0.47 | -0.38 | 0.53 | 0.75 | 0.71 | 0.80 | 0.54 | 0.24 | 1.06 | 2.23 | 1.77 | 1.64 |
| | Bradi2g47220 | -0.40 | 0.27 | 0.55 | 0.63 | 0.19 | 0.35 | 0.46 | 0.29 | -0.08 | -0.02 | 0.82 | 1.22 | 0.81 | 0.99 | 1.03 | 0.15 | 0.69 | 1.16 | 0.83 | 0.70 |
| | Bradi2g02720 | -0.62 | -0.43 | 0.99 | 2.91 | 1.94 | 1.43 | 0.89 | -1.44 | -0.56 | -0.43 | 0.72 | -0.06 | 0.00 | -0.49 | 1.27 | -1.94 | -1.90 | -2.08 | -0.17 | -0.06 |
| | Bradi2g02710 | -0.40 | 0.00 | -0.39 | 0.43 | -0.04 | 0.26 | -0.02 | -0.61 | -0.30 | -0.91 | 0.70 | 0.45 | 0.89 | -0.10 | -0.07 | 0.10 | 0.39 | 0.82 | 0.36 | -1.02 |
| AP2 | Bradi2g37800 | -0.50 | -0.33 | -0.25 | 0.19 | -0.18 | -1.60 | -0.82 | -0.87 | -0.48 | -1.00 | -0.56 | -0.43 | -1.03 | -0.63 | -1.04 | 0.37 | 0.08 | -0.04 | -0.09 | -0.06 |
| | Bradi5g24100 | 0.76 | -0.18 | 1.58 | 2.39 | 1.84 | -0.20 | -0.29 | 0.34 | 0.61 | 0.65 | -0.16 | -0.36 | -0.45 | 0.02 | 0.12 | 0.39 | 0.44 | 0.62 | 0.07 | 0.21 |
| | Bradi1g53650 | 0.21 | -0.07 | -0.13 | -0.29 | 0.48 | -1.48 | -1.25 | -1.27 | -1.48 | -1.11 | -1.00 | -1.15 | -1.67 | -1.56 | -1.86 | -0.02 | -0.26 | -0.35 | -0.38 | -1.15 |
| | Bradi1g03880 | -0.44 | -0.40 | -0.29 | -0.22 | -0.36 | -0.14 | -0.29 | -0.55 | -0.04 | -0.20 | -1.07 | -0.64 | -1.27 | -0.71 | -0.61 | -0.46 | -0.56 | -0.98 | -0.96 | -0.96 |
| | Bradi1g30337 | -0.22 | -0.38 | 0.10 | 0.63 | 0.05 | -0.19 | -0.15 | -0.44 | -0.56 | -1.01 | 0.20 | 0.72 | 0.66 | 0.59 | 0.82 | -0.93 | -0.73 | 0.01 | -0.55 | 0.06 |
| | Bradi2g53070 | -0.12 | 0.67 | 0.51 | -0.84 | 0.15 | -0.30 | -0.38 | -0.40 | -1.61 | -1.05 | 1.40 | 0.75 | 1.59 | 0.92 | 1.78 | 0.68 | 0.48 | -0.45 | -1.90 | -0.20 |
| | Bradi3g59300 | 0.29 | 0.14 | 0.02 | 0.73 | 1.23 | -0.34 | -0.14 | -0.16 | 0.01 | 0.17 | 0.89 | 0.30 | -0.16 | 0.02 | 0.29 | 0.13 | 0.01 | -0.11 | -0.08 | 0.56 |
| | Bradi3g15880 | 0.01 | -0.09 | -0.13 | 0.03 | 0.39 | 1.02 | 0.44 | 0.64 | 0.34 | 0.47 | -1.07 | -1.55 | -1.14 | -1.51 | -0.95 | -0.08 | 0.04 | -0.45 | 0.13 | 0.08 |
| | Bradi2g48130 | 0.56 | 0.58 | 1.14 | 1.12 | -0.31 | -0.78 | -0.46 | -0.44 | -0.60 | -0.76 | -1.23 | -0.65 | -0.36 | -0.07 | -0.69 | -0.55 | -0.80 | -0.99 | -0.66 | -0.10 |
| | Bradi1g51045 | 0.07 | 0.13 | -0.24 | 0.03 | 0.09 | 0.08 | 0.02 | -0.32 | -0.23 | -0.28 | 0.00 | -0.48 | -0.34 | -0.62 | 0.07 | -0.25 | -0.59 | -0.11 | 0.04 | 0.01 |
| | Bradi2g18570 | -0.17 | 0.13 | -0.11 | 0.01 | 0.12 | 0.62 | 0.44 | 0.41 | -0.78 | -0.04 | 0.64 | -0.03 | -0.03 | -0.63 | 0.62 | -0.39 | -0.05 | 0.37 | 0.00 | 0.06 |
| | Bradi4g30617 | -0.01 | -0.44 | 0.26 | 0.18 | 0.10 | -0.95 | -0.89 | -0.21 | -0.78 | -0.13 | -0.07 | 0.28 | -0.02 | 0.77 | 0.27 | -0.41 | -0.16 | -0.45 | -0.12 | -0.48 |
| | Bradi2g26987 | 0.18 | 0.12 | 0.34 | 0.24 | -0.37 | 0.22 | 0.30 | 0.24 | 0.07 | -0.27 | -0.52 | -0.26 | 0.09 | -0.36 | 0.34 | -0.52 | -0.41 | -0.75 | -0.67 | -0.53 |
| | Bradi4g43877 | 0.03 | -0.29 | 0.14 | 0.21 | -0.03 | 0.11 | 0.13 | 0.25 | 0.52 | 0.51 | -0.11 | 0.21 | -0.04 | 0.33 | 0.41 | 0.26 | 0.61 | 0.43 | 0.07 | -0.26 |
| | Bradi1g57560 | -0.17 | -0.10 | -0.06 | 0.06 | 0.15 | 0.09 | 0.20 | -0.19 | -0.26 | -0.36 | 0.01 | 0.23 | 0.16 | 0.12 | 0.20 | -0.10 | -0.22 | 0.47 | 0.02 | -0.17 |
| | Bradi1g07290 | 0.15 | 0.43 | -0.17 | -0.02 | 0.26 | 0.40 | 0.38 | -0.15 | -0.28 | -0.21 | 0.09 | 0.24 | 0.49 | 0.15 | 0.22 | -0.16 | -0.14 | 0.33 | -0.12 | 0.34 |
| | Bradi1g72890 | 0.26 | 0.05 | 0.05 | 0.53 | 1.49 | -0.21 | -0.30 | -0.65 | -0.11 | -0.55 | 0.37 | 0.61 | 0.41 | 0.67 | 0.00 | -0.06 | 0.13 | 0.61 | 0.49 | 0.50 |
| | Bradi5g24360 | -0.12 | -0.08 | 0.14 | 0.07 | 0.07 | -0.23 | 0.14 | -0.28 | -0.57 | -0.12 | -0.27 | 0.30 | 0.18 | -0.25 | 0.38 | 0.39 | -0.04 | 0.33 | 0.07 | 0.04 |
| | Bradi2g57747 | 0.30 | -0.55 | -0.61 | 0.14 | 0.12 | -0.28 | -0.47 | -0.18 | -0.58 | -0.41 | -0.25 | 0.37 | -0.55 | -0.26 | 0.07 | -0.21 | -0.55 | 1.02 | 0.25 | 0.13 |
| | Bradi1g64240 | -0.10 | 0.44 | 0.81 | 0.46 | -0.13 | 0.24 | 0.64 | -0.23 | 0.08 | -0.41 | -0.21 | 0.49 | 0.34 | 0.36 | 0.32 | 1.04 | 0.42 | 0.48 | 0.48 | 0.04 |
| | Bradi5g14960 | 0.09 | 0.32 | 0.52 | -0.01 | 0.31 | 0.43 | 0.23 | 0.25 | -0.23 | -0.71 | 0.25 | -0.09 | 1.76 | 0.39 | 0.51 | -0.08 | 0.33 | 0.60 | -0.11 | 0.45 |
| | Bradi1g31337 | 0.46 | -0.43 | -0.58 | 0.00 | 0.29 | 1.08 | 0.48 | -0.59 | -0.06 | -0.34 | -0.20 | 0.07 | 0.37 | 0.31 | 0.51 | -0.01 | 0.01 | -0.17 | -0.15 | -0.64 |
| | Bradi3g48697 | -0.48 | -0.08 | -0.52 | -0.12 | 0.01 | -0.17 | 0.20 | -0.07 | -0.26 | 0.32 | -0.33 | -0.29 | -1.04 | -0.58 | 0.27 | -0.22 | -0.15 | -0.96 | -0.99 | -0.41 |
| DREB | Bradi2g60340 | 2.91 | 3.12 | 1.89 | 1.92 | 0.55 | 0.25 | 1.31 | 0.31 | 0.53 | 0.15 | 0.12 | -0.08 | -0.04 | -0.30 | 0.70 | -0.66 | -0.40 | -0.50 | -0.33 | -0.24 |

| | | | | | | | | | | | | | | | | | | | | |
|---|---|---|---|---|---|---|---|---|---|---|---|---|---|---|---|---|---|---|---|---|
| Bradi2g60331 | 2.89 | 3.58 | 2.28 | 2.37 | 1.32 | -0.18 | 2.21 | 1.17 | 1.20 | 1.38 | -0.69 | -0.82 | -2.26 | -0.32 | -0.13 | -0.32 | -0.14 | -0.62 | -0.92 | -0.18 |
| Bradi3g57360 | -0.44 | -0.74 | -0.30 | 0.00 | 0.10 | -0.47 | -1.57 | -0.64 | -1.39 | -0.94 | 0.35 | -0.30 | -0.28 | 0.41 | 0.96 | -0.13 | -0.82 | 0.09 | -0.66 | -0.75 |
| Bradi4g35610 | 4.42 | 3.99 | 3.43 | 1.97 | -0.29 | 0.53 | 1.28 | -0.36 | -0.33 | -0.68 | 0.28 | 0.17 | -0.27 | 0.33 | 0.12 | 0.11 | -0.78 | 0.35 | 0.03 | -0.13 |
| Bradi4g35570 | 5.48 | 5.63 | 5.94 | 3.12 | 0.52 | -0.02 | 1.14 | 0.04 | -0.44 | -0.56 | 0.34 | 0.48 | 0.45 | 0.05 | 1.05 | -0.27 | -0.52 | 0.37 | 0.39 | 0.11 |
| Bradi4g35580 | #N/A | #N/A | #N/A | #N/A | #N/A | #N/A | #N/A | #N/A | #N/A | #N/A | #N/A | #N/A | #N/A | #N/A | #N/A | #N/A | #N/A | #N/A | #N/A | #N/A |
| Bradi4g35600 | 6.21 | 5.96 | 4.58 | 4.62 | 1.95 | 0.52 | 2.32 | -0.80 | 0.46 | -0.14 | 0.37 | 0.51 | -0.25 | -0.03 | 0.78 | 0.09 | 0.02 | 0.29 | -0.57 | -0.02 |
| Bradi4g35620 | 3.53 | 3.54 | 3.10 | 2.48 | 0.38 | 0.05 | 1.22 | -0.21 | -0.05 | -0.06 | 0.54 | 0.73 | 1.01 | 1.63 | 0.52 | 0.38 | 0.20 | 0.10 | 0.56 | -0.50 |
| Bradi4g35590 | 2.10 | 1.84 | 1.60 | 1.18 | -0.13 | 0.14 | 0.60 | -0.32 | -0.47 | -0.10 | 0.20 | -0.16 | 0.24 | 0.14 | 0.61 | 0.04 | 0.19 | 0.09 | -0.23 | 0.66 |
| Bradi1g57970 | 0.21 | 0.62 | 1.38 | 1.36 | 1.13 | -0.02 | 0.70 | -0.09 | -0.25 | -0.08 | 0.51 | 0.43 | 0.35 | -0.19 | 1.08 | 0.81 | 0.53 | 0.83 | 0.41 | 0.01 |
| Bradi1g77120 | 2.39 | 2.46 | 2.04 | 1.63 | 0.53 | -0.25 | 0.42 | -0.32 | -0.55 | -0.48 | 0.36 | -0.10 | -0.16 | -0.09 | 0.87 | -0.07 | 0.01 | 0.64 | 1.06 | 0.95 |
| Bradi4g35650 | 2.95 | 2.84 | 1.93 | 0.15 | 0.03 | 0.54 | 1.39 | -0.19 | 0.32 | 1.03 | 0.89 | 0.27 | 0.58 | 1.47 | 1.62 | -0.03 | 0.35 | 0.45 | 0.75 | 0.76 |
| Bradi4g35630 | 4.06 | 4.05 | 2.20 | 0.42 | -0.03 | 0.46 | 1.27 | 0.16 | -0.20 | -0.06 | 0.40 | 0.58 | 0.32 | 0.51 | 0.86 | -0.06 | -0.46 | 0.45 | 0.25 | -0.17 |
| Bradi3g51630 | 2.08 | 1.97 | 2.72 | 3.70 | 0.16 | -0.90 | -0.08 | -0.62 | 0.21 | -1.04 | 2.42 | -0.38 | -0.45 | 2.04 | 1.65 | 2.33 | -4.47 | 0.04 | -0.10 | -0.16 |
| Bradi1g49570 | 2.35 | 3.03 | 3.32 | 3.09 | 0.25 | 0.31 | 1.27 | -0.51 | -0.31 | 0.20 | 2.67 | 2.28 | 1.49 | 2.08 | 2.62 | 0.45 | -1.39 | 1.33 | 0.71 | 0.48 |
| Bradi1g49560 | 0.56 | 1.22 | 0.99 | 0.65 | -0.46 | -0.18 | 0.51 | 0.33 | -0.12 | -0.33 | 4.30 | 3.67 | 3.71 | 3.59 | 3.77 | 1.34 | 0.41 | 2.05 | 1.21 | 1.60 |
| Bradi3g50620 | 0.28 | -0.04 | -0.36 | 0.21 | 0.33 | 1.10 | 0.50 | -0.19 | -0.11 | 0.11 | 0.24 | 0.17 | 0.60 | 0.36 | 0.95 | -0.13 | -0.29 | -0.22 | 0.11 | -0.27 |
| Bradi5g17610 | 0.58 | 0.39 | 0.38 | 0.81 | 0.29 | -0.38 | -0.48 | -1.15 | -0.82 | -1.05 | 0.18 | 0.32 | 0.23 | 0.55 | 0.03 | 0.06 | -0.39 | 0.62 | -0.22 | -0.83 |
| Bradi5g17630 | 0.12 | 0.22 | 0.31 | 0.54 | -0.11 | -0.57 | -0.20 | -0.11 | -0.52 | -0.86 | -0.22 | -0.54 | -0.22 | -0.54 | 0.39 | 0.18 | -0.02 | 0.69 | -0.01 | 0.46 |
| Bradi3g50625 | -0.38 | 0.07 | -0.16 | -0.20 | -0.17 | -0.03 | -0.48 | -0.16 | -0.33 | -0.64 | -0.06 | 0.09 | 0.37 | -0.05 | 0.54 | -0.06 | -0.35 | -0.54 | -0.29 | -0.30 |
| Bradi5g17640 | 0.65 | 0.69 | 1.28 | 1.22 | 0.65 | 0.19 | 0.40 | 0.79 | 0.51 | 0.35 | 1.44 | 1.14 | 1.59 | 1.13 | 1.18 | 1.77 | 0.98 | 1.43 | 1.35 | 1.29 |
| Bradi3g50630 | -0.22 | -0.06 | 0.18 | -0.15 | -0.64 | -0.57 | -0.59 | 0.04 | -0.55 | -0.39 | -0.13 | -0.15 | -0.09 | -0.99 | 0.52 | -0.27 | -0.78 | -0.63 | -0.65 | -0.82 |
| Bradi5g18850 | 0.23 | 0.07 | 0.21 | 0.25 | 0.20 | 0.02 | 0.10 | -0.01 | 0.01 | 0.02 | -0.10 | -0.01 | -0.04 | -0.15 | -0.37 | -0.16 | 0.06 | 0.20 | 0.03 | 0.18 |
| Bradi3g51610 | 0.00 | 0.39 | 0.15 | 0.53 | 0.27 | 0.59 | 0.88 | -0.31 | -0.33 | 0.28 | 0.39 | 0.13 | 0.23 | 1.05 | 0.58 | 0.05 | -0.07 | 1.00 | 0.04 | 0.42 |
| Bradi3g31600 | -0.09 | 0.12 | -0.06 | 0.18 | 0.10 | 0.08 | 0.16 | 0.13 | -0.05 | -0.12 | 0.38 | 0.49 | 0.56 | 0.68 | 0.70 | 0.45 | 0.36 | 0.64 | 0.24 | 0.27 |
| Bradi5g17620 | 0.11 | 0.01 | -0.05 | 0.13 | 0.25 | 0.39 | 1.25 | 1.61 | 1.19 | 0.97 | 0.07 | 0.02 | 0.06 | -0.19 | 0.59 | 0.44 | 0.43 | 0.40 | -0.17 | 0.35 |
| Bradi3g33355 | -0.71 | 0.77 | 1.47 | 0.20 | 0.14 | -1.00 | 0.58 | -0.13 | -0.61 | -1.37 | -0.91 | -0.47 | -0.62 | -0.25 | -0.16 | -0.48 | -0.30 | -1.33 | -0.37 | -1.14 |
| Bradi3g08790 | 0.94 | 0.21 | 1.33 | 2.17 | 1.53 | 0.47 | -0.37 | -0.50 | 0.19 | -0.57 | -0.49 | -0.44 | -0.23 | -0.72 | -0.93 | -0.24 | -0.25 | 0.01 | -0.53 | -1.13 |
| Bradi1g38110 | -0.37 | 0.07 | -0.18 | 0.01 | 0.04 | 0.56 | 0.31 | 0.06 | -0.27 | -0.37 | -0.28 | 0.23 | 0.32 | 0.28 | 0.32 | 0.01 | -0.10 | 0.02 | -0.10 | -0.09 |
| Bradi2g06180 | -0.12 | -0.29 | -0.19 | 0.24 | 0.05 | 0.02 | 0.00 | 0.02 | 0.16 | -0.01 | -0.15 | 0.48 | -0.21 | -0.09 | 0.52 | 0.36 | 0.47 | 0.37 | 0.05 | 0.56 |
| Bradi3g45997 | -0.03 | 0.05 | -0.21 | 0.06 | 0.03 | 0.27 | 0.13 | -0.23 | -0.17 | -0.41 | -0.34 | 0.42 | 0.22 | 0.75 | 0.21 | 0.04 | 0.00 | 0.25 | 0.02 | -0.19 |
| Bradi4g21265 | 0.13 | 0.96 | -0.19 | 0.36 | 0.44 | 0.28 | 0.11 | -0.16 | 0.15 | 0.10 | 0.10 | 0.17 | 0.01 | -0.25 | 0.22 | -0.19 | -0.60 | 0.28 | 0.11 | -0.12 |
| Bradi5g11295 | 0.42 | -0.07 | 0.09 | -0.13 | 0.34 | 0.26 | 0.21 | -0.22 | -0.17 | -0.31 | 0.23 | -0.18 | 0.37 | -0.48 | 0.89 | -0.18 | -0.11 | 0.04 | -0.01 | 0.28 |

| | | | | | | | | | | | | | | | | | | | | |
|---|---|---|---|---|---|---|---|---|---|---|---|---|---|---|---|---|---|---|---|---|
| Bradi2g25050 | 0.03 | -0.12 | 0.11 | 0.62 | -0.26 | 0.36 | -0.08 | -0.44 | -0.06 | -0.34 | 0.28 | -0.01 | -0.10 | -0.06 | 0.36 | -0.10 | -0.13 | 0.44 | 0.35 | -0.09 |
| Bradi2g57201 | -0.53 | 0.10 | -0.26 | 0.48 | 0.03 | 0.16 | 0.04 | 0.33 | -0.30 | -0.32 | 0.33 | -0.12 | 0.10 | -0.02 | 0.33 | 0.37 | -0.38 | 0.30 | 0.19 | 0.04 |
| Bradi3g37544 | 0.29 | 1.25 | 0.30 | 0.51 | 1.31 | 0.35 | 0.95 | 0.78 | 0.08 | 0.45 | 0.37 | 0.57 | 0.66 | 0.23 | 0.51 | -0.20 | -1.52 | 0.87 | -0.46 | -0.59 |
| Bradi3g60018 | 0.20 | 0.03 | 0.02 | 0.14 | 0.34 | -0.14 | 0.21 | 0.23 | 0.05 | -0.38 | 0.06 | 0.56 | -0.12 | 1.13 | 0.80 | -0.22 | -0.10 | 0.22 | -0.08 | 0.08 |
| Bradi3g56801 | 0.90 | 0.48 | 0.52 | 0.03 | -0.17 | 0.09 | -0.26 | -0.33 | 0.14 | -0.35 | 0.55 | 0.38 | 0.60 | 0.60 | 0.97 | 0.47 | -0.66 | 0.14 | -0.16 | -0.12 |
| Bradi5g24700 | 0.46 | -0.48 | 0.84 | -1.29 | -1.89 | 0.90 | 0.07 | 0.41 | -0.59 | -1.22 | 1.98 | 0.12 | -0.25 | -0.17 | 0.55 | -0.16 | 0.24 | 1.43 | 0.72 | 1.04 |
| Bradi1g67350 | -0.02 | 0.01 | -0.24 | 0.17 | -0.25 | 3.52 | 1.93 | 1.58 | 1.55 | 0.33 | -0.36 | -0.16 | 0.36 | 0.22 | 0.82 | 0.48 | 0.02 | -0.14 | -0.32 | -0.12 |
| Bradi3g57862 | -0.18 | -0.08 | -0.18 | -0.19 | -0.16 | -0.37 | -0.21 | -0.07 | -0.10 | -0.01 | -0.05 | -0.40 | -0.86 | -0.74 | -0.80 | -0.23 | -0.38 | -0.36 | -0.26 | -0.08 |
| Bradi1g46120 | 0.24 | 0.40 | 0.42 | -0.05 | 0.09 | 0.15 | 0.13 | -0.02 | -0.24 | -0.25 | 0.07 | 0.18 | 0.09 | 0.17 | 0.33 | 0.10 | 0.18 | 0.00 | -0.01 | -0.19 |
| Bradi1g48320 | 0.29 | -0.01 | -0.21 | 0.42 | 0.50 | 0.30 | 0.05 | -0.49 | -0.01 | -0.21 | 0.92 | 0.87 | 0.57 | 1.47 | 0.67 | 0.92 | 0.52 | 1.82 | 1.46 | 1.82 |
| Bradi5g24110 | 0.38 | -0.11 | 0.46 | 1.01 | 0.44 | 0.21 | 0.05 | 0.37 | 0.33 | -0.34 | 1.01 | 0.38 | 0.72 | 0.62 | 0.87 | 0.77 | 0.75 | 0.90 | 0.98 | 1.02 |
| Bradi5g24710 | 0.34 | -0.01 | -0.01 | 0.27 | -0.09 | 0.62 | 0.25 | -0.35 | -0.05 | -0.04 | 0.15 | 0.31 | -0.13 | -0.34 | 0.34 | -0.48 | 0.08 | 0.45 | 0.28 | 0.52 |
| Bradi3g54160 | 0.20 | 0.22 | -0.14 | -0.11 | 0.02 | 0.23 | 0.23 | 0.06 | 0.22 | 0.33 | -0.36 | 0.26 | -0.39 | 0.79 | -0.01 | -0.02 | -0.14 | -0.09 | 0.18 | 0.07 |
| Bradi1g47480 | 0.00 | -1.18 | -0.76 | 0.21 | 0.33 | 0.21 | -0.69 | -1.00 | -0.24 | -0.82 | -0.05 | -0.19 | 1.17 | -0.81 | 0.77 | -0.61 | -0.44 | 0.00 | -0.63 | -0.20 |
| Bradi5g24720 | 0.17 | 1.21 | 0.32 | -1.08 | 0.73 | -0.17 | 1.27 | 1.35 | -0.34 | -0.99 | 0.11 | 0.17 | -0.36 | 0.05 | 0.48 | -0.31 | 1.42 | -1.86 | -1.02 | 1.00 |
| Bradi1g36590 | 0.07 | 0.20 | 0.63 | 0.24 | 0.20 | -0.48 | -0.86 | -0.74 | -0.76 | -0.75 | -0.13 | -0.53 | -1.08 | -1.72 | -0.48 | -0.02 | -0.20 | -1.37 | -1.36 | -1.63 |
| Bradi3g07450 | -0.30 | -0.11 | 0.10 | 0.21 | 0.38 | -0.45 | -0.30 | -0.84 | -1.19 | -1.11 | -0.03 | 0.09 | 0.03 | 0.12 | 0.14 | -0.17 | 0.27 | -0.45 | -0.62 | -0.25 |
| Bradi2g29010 | -0.58 | -0.20 | -0.02 | 0.42 | -0.02 | 0.17 | 0.70 | -0.28 | -0.06 | 0.29 | 0.39 | 0.23 | 0.28 | -0.23 | 1.13 | 0.42 | 0.05 | 0.25 | 0.13 | -0.29 |
| Bradi1g72990 | -0.21 | 0.05 | 0.11 | 0.15 | 0.09 | -0.21 | -0.04 | -0.32 | -0.24 | -0.26 | -0.06 | 0.09 | 0.20 | 0.15 | 0.48 | -0.28 | 0.10 | 0.20 | -0.01 | 0.08 |
| Bradi3g12680 | 0.08 | -0.23 | -0.13 | 0.19 | 0.20 | 0.39 | -0.21 | 0.08 | 0.26 | -0.44 | 0.37 | 0.29 | 0.11 | 0.64 | 0.55 | 0.13 | -0.07 | 0.62 | 0.42 | -0.25 |
| Bradi2g04000 | 0.40 | -0.19 | 0.00 | -0.13 | -0.06 | 0.28 | 0.44 | 0.70 | 1.19 | 1.02 | 0.28 | 0.15 | 0.25 | 0.61 | 0.18 | 0.17 | 0.46 | 0.58 | 0.72 | 0.75 |
| Bradi2g29960 | 0.60 | 0.53 | 0.22 | 0.36 | 0.78 | 1.56 | 1.68 | 1.65 | 1.18 | 1.08 | 0.79 | 1.09 | 1.80 | 3.09 | 3.17 | 0.61 | 1.05 | 1.79 | 1.67 | 1.21 |
| Bradi2g15847 | 0.23 | 0.25 | 0.36 | 0.65 | 0.00 | 0.98 | 0.40 | -0.35 | -0.24 | -0.10 | 0.25 | 0.04 | 0.33 | 0.35 | 0.71 | 0.51 | -0.62 | 0.87 | 1.84 | 0.71 |
| Bradi5g16450 | 0.76 | 0.49 | 0.46 | 0.57 | 0.13 | 0.35 | 0.70 | 0.72 | 0.43 | 0.00 | 0.49 | 0.85 | 0.91 | 1.25 | 1.20 | 0.30 | 0.19 | -0.07 | 0.41 | 0.63 |
| Bradi3g49810 | 0.05 | 0.84 | 0.32 | 0.42 | 0.40 | 1.93 | 2.01 | 3.11 | 1.37 | 1.46 | 3.88 | 3.41 | 3.26 | 3.46 | 4.05 | 3.50 | -0.36 | 3.20 | 3.08 | 1.40 |
| Bradi3g35560 | 0.94 | 0.44 | 0.27 | -0.03 | -0.43 | 1.27 | 0.77 | 1.85 | 0.63 | 0.77 | 4.25 | 3.68 | 3.43 | 3.94 | 3.76 | 2.38 | 1.25 | 2.29 | 1.79 | 1.56 |
| Bradi4g29010 | 0.19 | 0.09 | 0.15 | 0.15 | 0.26 | -0.66 | -0.61 | -0.39 | -0.54 | -0.68 | 0.64 | 0.08 | -0.13 | 0.32 | 0.42 | 0.13 | -0.16 | 0.29 | 0.27 | 0.22 |
| Bradi1g71740 | 0.21 | 0.34 | -0.01 | -0.54 | -0.41 | 1.36 | 0.71 | 0.26 | -0.36 | 0.00 | 3.92 | 2.44 | 2.10 | 2.42 | 1.85 | 1.80 | 0.41 | 1.41 | 0.98 | 0.51 |
| Bradi3g24000 | -0.22 | -0.19 | -0.48 | -0.32 | -0.21 | -0.21 | -0.43 | 0.20 | -0.89 | 0.21 | 0.43 | 0.57 | 0.14 | 0.06 | 0.44 | 0.13 | -0.36 | 0.75 | 0.62 | -0.45 |
| Bradi3g58980 | 0.36 | 0.19 | 0.16 | 0.86 | 0.67 | 1.72 | 1.04 | 1.28 | 1.72 | 1.22 | 1.32 | 1.28 | 1.89 | 2.43 | 1.93 | 0.41 | -0.23 | 1.06 | 1.15 | 1.02 |
| Bradi1g45470 | -0.11 | -0.06 | -0.16 | 0.56 | 0.48 | -0.42 | -0.57 | -0.40 | 0.14 | 0.01 | 1.30 | 1.35 | 2.48 | 2.43 | 2.95 | 0.92 | 0.62 | 1.37 | 2.29 | 1.04 |
| Bradi2g22505 | 0.32 | 0.32 | 0.04 | 0.46 | -0.22 | 0.41 | 0.22 | 0.16 | 0.51 | 0.14 | -0.10 | -0.04 | -0.04 | -0.05 | -0.03 | 0.00 | -0.26 | 0.92 | 0.21 | -0.12 |

| | | | | | | | | | | | | | | | | | | | | | |
|---|---|---|---|---|---|---|---|---|---|---|---|---|---|---|---|---|---|---|---|---|---|
| ERF | Bradi1g53756 | 0.06 | 0.20 | 0.17 | 0.32 | 0.07 | 0.12 | 0.64 | 0.07 | 0.09 | -0.47 | 0.32 | 0.03 | 1.07 | -0.41 | 0.85 | 0.19 | -0.27 | 0.10 | 0.08 | 0.31 |
| | Bradi1g53753 | 0.01 | -0.17 | -0.49 | 0.02 | 0.40 | -0.07 | 0.06 | -0.32 | 0.18 | 0.07 | -0.19 | 0.35 | 0.12 | 0.54 | -0.26 | 0.08 | 0.36 | 0.30 | 0.40 | 0.36 |
| | Bradi1g23756 | -0.53 | -0.01 | -0.33 | 0.49 | 0.81 | 0.88 | 0.01 | 0.29 | -0.16 | 0.15 | 0.26 | 0.00 | 0.60 | 0.43 | 0.27 | 0.04 | -0.08 | 0.38 | 0.07 | -0.19 |
| | Bradi1g54450 | 0.20 | 0.00 | -0.11 | 0.51 | 0.07 | 0.64 | 0.64 | 1.18 | 1.49 | 1.50 | 0.33 | -0.03 | 0.41 | -0.19 | 0.89 | 0.54 | 0.31 | 1.89 | 2.35 | 2.22 |
| | Bradi1g38238 | -0.26 | 0.25 | -0.19 | 0.05 | -0.31 | -0.53 | -0.63 | -0.94 | -0.70 | -0.91 | 0.23 | 0.02 | 0.07 | 0.29 | 0.20 | -0.06 | -0.22 | -0.56 | -0.51 | -0.44 |
| | Bradi4g03371 | -0.31 | -0.17 | -0.24 | 0.01 | 0.18 | -0.30 | 0.02 | 0.13 | -0.07 | -0.32 | 0.25 | 0.26 | 0.17 | 0.69 | 0.70 | -0.11 | -0.15 | 0.20 | -0.09 | 0.07 |
| | Bradi4g35655 | -0.22 | -0.01 | 0.02 | 0.02 | -0.47 | 0.01 | 0.00 | -0.54 | -0.61 | -0.74 | -0.20 | -0.33 | 0.08 | 0.27 | 0.01 | 0.21 | 0.13 | 0.35 | -0.55 | -0.66 |
| | Bradi2g24175 | 0.20 | 0.06 | -0.05 | -0.19 | 0.39 | 1.94 | 0.09 | -0.12 | -0.63 | -0.10 | 1.21 | 1.48 | 2.31 | 2.50 | 2.57 | 1.45 | 2.20 | 1.99 | 1.29 | 0.97 |
| | Bradi2g56145 | -0.64 | 0.10 | 0.21 | 1.32 | 1.33 | 0.75 | 1.46 | 0.29 | 0.58 | 0.54 | 0.45 | 1.35 | 1.42 | 1.39 | 2.09 | 1.92 | 2.56 | 2.30 | 2.24 | 2.18 |
| | Bradi4g41616 | 0.20 | 0.40 | -0.17 | 0.93 | 1.68 | 0.20 | 0.55 | 0.29 | 0.88 | 1.56 | 3.09 | 4.90 | 5.31 | 5.96 | 5.81 | 3.10 | 3.13 | 3.93 | 3.12 | 3.91 |
| | Bradi5g08380 | 0.27 | -1.09 | -0.45 | 0.60 | 0.74 | 0.62 | 0.34 | 0.49 | 1.16 | 1.45 | 0.07 | 0.19 | 0.23 | 0.48 | 0.52 | 1.47 | 2.21 | 3.60 | 3.05 | 2.32 |
| | Bradi3g58015 | 0.88 | 2.01 | 2.16 | 1.88 | -0.27 | 0.59 | 1.35 | 0.42 | 0.01 | -0.26 | 3.24 | 4.26 | 5.43 | 5.34 | 5.45 | 1.93 | 1.19 | 2.67 | 2.74 | 3.29 |
| | Bradi3g38140 | 1.23 | 1.80 | 1.23 | 0.71 | 0.15 | 0.42 | 0.23 | -0.09 | -0.18 | 0.13 | 1.18 | 0.63 | 1.16 | 0.82 | 0.57 | 1.12 | -0.58 | 0.42 | 0.51 | -0.18 |
| | Bradi4g27850 | 0.26 | -0.08 | -0.24 | 0.06 | 0.14 | -0.08 | 0.16 | 0.16 | 0.46 | 0.43 | -0.01 | 0.32 | 0.50 | 0.61 | -0.16 | -0.05 | -0.08 | 0.25 | 0.62 | 0.11 |
| | Bradi3g40343 | -0.10 | -0.20 | -0.29 | -0.11 | 0.74 | 0.19 | 0.19 | 0.90 | 0.28 | -0.27 | 0.14 | 0.29 | 0.15 | 0.16 | 0.57 | 0.11 | -0.23 | 0.41 | -0.22 | -0.03 |
| | Bradi2g02100 | 0.14 | 0.33 | 0.39 | 0.50 | 0.50 | 0.18 | 0.35 | 0.42 | 0.53 | 0.97 | -0.39 | -0.63 | -0.40 | -0.41 | -0.58 | -0.28 | -0.35 | -0.19 | -0.19 | 0.02 |
| | Bradi3g60120 | 0.22 | 0.20 | 0.46 | 0.60 | 0.23 | -0.81 | -0.86 | -1.16 | -0.58 | -0.88 | 0.40 | 0.09 | 1.32 | 0.43 | 0.54 | 0.26 | -0.12 | 0.79 | 0.34 | 0.50 |
| | Bradi4g31040 | 0.02 | 0.10 | 0.32 | 0.81 | 0.69 | 0.23 | 0.27 | 0.37 | 0.45 | 0.44 | 0.03 | 0.20 | 0.25 | 0.70 | -0.68 | 0.18 | 0.29 | 0.60 | 0.35 | 0.12 |
| | Bradi1g46690 | 0.18 | 0.23 | 0.21 | 0.51 | 0.88 | -0.06 | -0.02 | 0.19 | 0.46 | 0.19 | 0.19 | 0.46 | 0.24 | 0.45 | 0.50 | 0.45 | 0.28 | 0.51 | 0.58 | 0.35 |
| | Bradi1g72457 | #N/A | #N/A | #N/A | #N/A | #N/A | #N/A | #N/A | #N/A | #N/A | #N/A | #N/A | #N/A | #N/A | #N/A | #N/A | #N/A | #N/A | #N/A | #N/A | #N/A |
| | Bradi2g27920 | 0.55 | 0.14 | -0.04 | 0.08 | 0.32 | 0.70 | -0.29 | 0.10 | 0.44 | 0.22 | 0.35 | 0.03 | -0.08 | -0.19 | 0.40 | -0.18 | 0.52 | 0.40 | 0.36 | 0.43 |
| | Bradi2g11890 | 0.30 | 0.19 | -0.66 | 0.17 | -0.15 | 2.08 | 1.35 | 0.38 | -0.25 | -0.29 | -0.30 | 0.20 | -0.01 | 0.31 | 0.81 | 1.07 | 0.81 | 1.43 | 0.96 | 0.98 |
| | Bradi1g17961 | 0.02 | -0.02 | 0.02 | -0.03 | 0.09 | 0.05 | -0.12 | 0.08 | -0.20 | -0.26 | 0.32 | 0.21 | 0.02 | -0.06 | 0.56 | -0.25 | 0.04 | 0.12 | 0.04 | 0.43 |
| | Bradi5g17480 | -0.34 | -0.05 | 0.05 | 1.17 | 1.57 | 1.29 | 0.63 | 0.14 | 0.53 | 1.19 | 0.65 | 1.03 | 0.32 | 2.11 | 1.57 | 0.07 | 0.11 | 0.06 | 0.48 | 1.00 |
| | Bradi3g50490 | 1.75 | 1.68 | 0.26 | -0.14 | -0.97 | 1.57 | 1.63 | 0.94 | 1.64 | 1.91 | 1.74 | 0.60 | 1.32 | 0.93 | 1.00 | 1.69 | 1.76 | 3.15 | 2.01 | -0.04 |
| | Bradi3g15084 | -0.62 | -0.50 | 0.24 | -0.25 | 0.27 | -0.32 | -0.35 | 0.27 | -0.17 | 0.39 | -0.02 | 0.36 | 0.62 | 0.53 | 0.18 | 0.42 | 0.15 | -0.19 | -0.31 | 0.21 |
| | Bradi1g00666 | 0.15 | 0.08 | -0.49 | -0.11 | 0.34 | 0.13 | 0.10 | -0.07 | -0.50 | -0.06 | -0.12 | -0.18 | -0.26 | -0.04 | -0.08 | -0.11 | -0.10 | 0.41 | 0.55 | 0.35 |
| | Bradi3g12566 | 0.06 | -0.13 | -0.29 | -0.07 | -0.06 | -0.39 | -0.27 | -0.57 | -0.44 | -0.46 | -0.12 | -0.11 | -0.27 | -0.19 | 0.10 | -0.35 | -0.20 | -0.12 | -0.15 | -0.40 |
| | Bradi3g12567 | 0.03 | 0.19 | 0.07 | 0.26 | 0.37 | 0.22 | 0.42 | 0.12 | 0.20 | 0.16 | 0.06 | 0.12 | 0.48 | 0.13 | 1.04 | -0.27 | 0.17 | 0.42 | 0.05 | 0.05 |
| | Bradi4g38941 | -0.62 | 0.47 | 0.27 | 0.42 | 0.53 | -0.17 | 0.68 | -0.07 | 0.42 | 0.59 | 0.42 | -0.02 | -0.25 | 1.33 | 0.48 | -0.04 | 0.35 | 0.01 | 0.28 | 0.72 |
| | Bradi3g12565 | 0.20 | 0.02 | 0.15 | 0.18 | 0.25 | 0.41 | 0.26 | 0.35 | 0.20 | 0.01 | -0.36 | 0.04 | 0.30 | 0.57 | 0.59 | 0.14 | 0.08 | 0.46 | 0.02 | -0.04 |
| | Bradi3g27690 | -0.63 | -1.23 | -0.91 | -0.23 | 0.06 | 0.91 | -0.84 | -0.54 | -0.37 | -0.16 | 0.51 | -0.46 | -0.28 | -0.90 | 0.69 | -0.51 | -0.74 | 1.77 | 0.54 | 0.14 |

| | | | | | | | | | | | | | | | | | | | | |
|---|---|---|---|---|---|---|---|---|---|---|---|---|---|---|---|---|---|---|---|---|
| Bradi1g75040 | 0.94 | -0.31 | 1.32 | 3.99 | 3.67 | 0.60 | 0.04 | -0.22 | -0.24 | -0.43 | 0.39 | 0.39 | 0.44 | -0.07 | 0.12 | 0.18 | -0.13 | 0.08 | -0.18 | -0.14 |
| Bradi3g50508 | -0.14 | -0.41 | -0.31 | 0.00 | -0.47 | 0.44 | 0.18 | 0.09 | -0.43 | -0.38 | 0.25 | 0.12 | 0.31 | -0.53 | 0.57 | 0.88 | 0.04 | 0.68 | 1.30 | 0.83 |
| Bradi5g17490 | -0.21 | -0.01 | 0.07 | 0.13 | -0.33 | 1.05 | 0.70 | 1.10 | 0.60 | 0.35 | 0.22 | 0.07 | 0.44 | -0.42 | 0.80 | -0.04 | 0.06 | 0.85 | 0.44 | 0.13 |
| Bradi5g25570 | 3.32 | 3.47 | 1.84 | 0.35 | -0.06 | 0.24 | 1.39 | 0.07 | 0.20 | 0.45 | 3.54 | 2.31 | 2.77 | 3.53 | 3.52 | 1.36 | -0.10 | 1.87 | 0.97 | 1.41 |
| Bradi2g16442 | -0.21 | -0.44 | -0.04 | 0.12 | -0.01 | -0.21 | 0.06 | -0.40 | -0.35 | -0.40 | 0.04 | 0.27 | 0.37 | 0.35 | 0.19 | -0.07 | -0.12 | 0.17 | 0.20 | -0.31 |
| Bradi2g07357 | -0.07 | 0.02 | -0.27 | -0.03 | 0.09 | 0.16 | 0.18 | 0.10 | -0.01 | 0.26 | 0.10 | 0.28 | -0.18 | -0.47 | 0.23 | 0.09 | -0.14 | 0.13 | -0.23 | -0.03 |
| Bradi2g45530 | 0.18 | -0.05 | -0.26 | -0.11 | 0.26 | 0.85 | 0.51 | -0.20 | -0.19 | -0.22 | 0.35 | -0.05 | 0.02 | 1.05 | 0.93 | -0.08 | -0.03 | 0.28 | -0.05 | -0.32 |
| Bradi1g48625 | 0.12 | 0.23 | 0.11 | 0.23 | 1.02 | 0.92 | 0.74 | 0.63 | 0.48 | 0.28 | -0.22 | 0.04 | -0.12 | -0.13 | 0.09 | -0.56 | -0.04 | 0.14 | -0.08 | 0.26 |
| Bradi2g31480 | 0.77 | 0.54 | 0.42 | 0.59 | 0.85 | 1.29 | 1.53 | 2.30 | 1.93 | 2.39 | 0.11 | -0.65 | 0.54 | 0.04 | -0.27 | 0.82 | 0.61 | 0.11 | 0.37 | 0.18 |
| Bradi2g50035 | 1.17 | 0.14 | 0.43 | -0.13 | -0.23 | 0.45 | 0.24 | 0.52 | 0.35 | 1.24 | 0.46 | -0.17 | 1.13 | 1.25 | 0.68 | 1.18 | 0.85 | -0.19 | 0.21 | 1.51 |
| Bradi1g04110 | -0.27 | -0.02 | -0.08 | -0.05 | -0.18 | -0.01 | 0.19 | 0.59 | 0.37 | 0.44 | -0.32 | 0.21 | 0.24 | 0.58 | 0.59 | 0.14 | 0.35 | 0.31 | 0.44 | 0.72 |
| Bradi3g47610 | 0.07 | 0.23 | 0.12 | 0.37 | 0.05 | 0.85 | 1.15 | -0.32 | -0.31 | -0.29 | -0.32 | 0.10 | 0.53 | 0.27 | 0.34 | -1.47 | -0.05 | 0.39 | 0.12 | -0.21 |
| Bradi2g21060 | 2.08 | 2.21 | 1.76 | 1.25 | 0.36 | -0.08 | 1.40 | 1.10 | 0.94 | 0.57 | 2.52 | 2.53 | 2.95 | 2.99 | 2.24 | 1.66 | 0.23 | 1.61 | 1.47 | 1.35 |
| Bradi2g52370 | 1.87 | 1.83 | 2.29 | 2.15 | 0.41 | -0.67 | 0.66 | 0.11 | 0.25 | -0.38 | 1.60 | 1.00 | 0.17 | 1.53 | 1.18 | -0.10 | -0.83 | 0.49 | 0.94 | -0.19 |
| Bradi3g06562 | -0.04 | -0.15 | -0.27 | -0.16 | 0.23 | 0.11 | 0.03 | 0.31 | 0.30 | 0.31 | 0.28 | 0.81 | 1.06 | 0.98 | 0.77 | 0.70 | 0.67 | 0.90 | 0.82 | 0.40 |
| Bradi5g21250 | 2.58 | 2.63 | 2.39 | 2.03 | 1.39 | -0.17 | 0.90 | 0.83 | 0.36 | 0.36 | 3.23 | 2.64 | 3.85 | 3.13 | 3.26 | 2.25 | 0.07 | 2.10 | 2.00 | 1.55 |
| Bradi3g18070 | 0.99 | 1.01 | 0.18 | -0.08 | 0.04 | 0.96 | 1.04 | 1.53 | 0.50 | 0.70 | 4.19 | 3.45 | 3.51 | 3.00 | 2.81 | 2.84 | 0.98 | 2.56 | 1.12 | 0.80 |
| Bradi3g18073 | 0.08 | -0.09 | -0.36 | -0.71 | 0.80 | 0.45 | 1.01 | -0.99 | -1.05 | 0.55 | 0.62 | 0.32 | -0.15 | -1.36 | 0.31 | 0.28 | -0.02 | 1.33 | 0.16 | -0.43 |
| Bradi5g08471 | -0.08 | -0.11 | -0.39 | 0.13 | 0.21 | 0.07 | -0.08 | -0.37 | -0.21 | -0.34 | -0.01 | 0.26 | 0.34 | 0.64 | 0.66 | 0.15 | 0.16 | 0.23 | 0.01 | 0.19 |
| Bradi3g44470 | 0.07 | 0.42 | 0.00 | 0.31 | -0.06 | 0.60 | 1.17 | 0.92 | 0.76 | -0.16 | -0.03 | 0.24 | -0.28 | -0.11 | 0.16 | 0.48 | 0.10 | 0.60 | 0.04 | -0.40 |
| Bradi1g18580 | -0.19 | 0.24 | 0.29 | 0.21 | 0.73 | -0.25 | 0.33 | -0.11 | 0.05 | -0.40 | -0.47 | 0.13 | 0.60 | 0.65 | -0.07 | 0.03 | 0.07 | 0.01 | -0.32 | 0.40 |
| Bradi1g33550 | 0.18 | 0.32 | 0.10 | -0.06 | -0.17 | 0.50 | 0.72 | 0.73 | 0.64 | 0.61 | -0.33 | 0.09 | 0.08 | -0.02 | -0.19 | -0.53 | -0.22 | 0.10 | 0.11 | -0.21 |
| Bradi3g04410 | -0.26 | -0.12 | 0.13 | 0.52 | 0.42 | 0.13 | 0.21 | 0.39 | 0.44 | 0.34 | 0.34 | 0.25 | 0.03 | -0.51 | 0.29 | 0.44 | -0.17 | 0.60 | 0.20 | 0.27 |
| Bradi2g21067 | 1.00 | 1.08 | 1.30 | 1.04 | 0.58 | -0.15 | 0.55 | 0.70 | 0.33 | -0.65 | 1.39 | -0.12 | -0.20 | -0.13 | 0.79 | 0.18 | -0.64 | 0.10 | -0.60 | -1.15 |
| Bradi2g52380 | -0.12 | -0.24 | 0.28 | 0.12 | -0.19 | 0.81 | 0.30 | 0.41 | -0.16 | 0.07 | -0.36 | -0.43 | -0.11 | -0.63 | 1.08 | 1.31 | -0.06 | -0.43 | -0.23 | 0.24 |

q value of treatment/control at each time point under the corresponding treatment

| sub_ family | gene | cold 1h | cold 2h | cold 5h | cold 10h | cold 24 | heat 1h | heat 2h | heat 5h | heat 10 | heat 24 | drought | drought | drought 5 | drought 1 | drought 2 | salt 1h | salt 2h | salt 5h | salt 10h | salt 24h |
|---|---|---|---|---|---|---|---|---|---|---|---|---|---|---|---|---|---|---|---|---|---|
| RAV | Bradi2g17610 | 0.60 | 0.37 | 0.15 | 0.07 | 0.46 | 0.16 | 0.72 | 0.18 | 0.13 | 0.18 | 0.25 | 0.12 | 0.23 | 0.16 | 0.09 | 0.50 | 0.01 | 0.00 | 0.00 | 0.01 |
| | Bradi2g47220 | 0.42 | 0.60 | 0.40 | 0.19 | 0.52 | 0.41 | 0.40 | 0.34 | 0.41 | 0.36 | 0.06 | 0.02 | 0.18 | 0.11 | 0.05 | 0.58 | 0.11 | 0.02 | 0.09 | 0.17 |
| | Bradi2g02720 | 0.37 | 0.19 | 0.32 | 0.00 | 0.02 | 0.11 | 0.21 | 0.03 | 0.13 | 0.18 | 0.33 | 0.66 | 0.70 | 0.46 | 0.01 | 0.01 | 0.01 | 0.00 | 0.40 | 0.43 |

| | | | | | | | | | | | | | | | | | | | | | |
|---|---|---|---|---|---|---|---|---|---|---|---|---|---|---|---|---|---|---|---|---|---|
| | Bradi2g02710 | 0.42 | 0.60 | 0.21 | 0.26 | 0.50 | 0.53 | 0.69 | 0.08 | 0.26 | 0.03 | 0.18 | 0.37 | 0.14 | 0.68 | 0.34 | 0.60 | 0.40 | 0.05 | 0.35 | 0.04 |
| AP2 | Bradi2g37800 | 0.37 | 0.25 | 0.32 | 0.53 | 0.32 | 0.02 | 0.21 | 0.03 | 0.06 | 0.02 | 0.06 | 0.30 | 0.05 | 0.23 | 0.02 | 0.11 | 0.56 | 0.51 | 0.44 | 0.43 |
| | Bradi5g24100 | 0.20 | 0.54 | 0.01 | 0.00 | 0.01 | 0.47 | 0.59 | 0.29 | 0.09 | 0.09 | 0.50 | 0.45 | 0.36 | 0.72 | 0.23 | 0.35 | 0.16 | 0.11 | 0.54 | 0.46 |
| | Bradi1g53650 | 0.60 | 0.51 | 0.32 | 0.26 | 0.13 | 0.02 | 0.17 | 0.02 | 0.00 | 0.01 | 0.01 | 0.02 | 0.01 | 0.01 | 0.00 | 0.55 | 0.24 | 0.24 | 0.20 | 0.02 |
| | Bradi1g03880 | 0.42 | 0.25 | 0.21 | 0.37 | 0.19 | 0.47 | 0.40 | 0.06 | 0.41 | 0.30 | 0.01 | 0.24 | 0.03 | 0.31 | 0.23 | 0.15 | 0.06 | 0.03 | 0.03 | 0.05 |
| | Bradi1g30337 | 0.60 | 0.25 | 0.45 | 0.26 | 0.56 | 0.47 | 0.59 | 0.13 | 0.09 | 0.01 | 0.57 | 0.15 | 0.28 | 0.38 | 0.05 | 0.05 | 0.06 | 0.49 | 0.12 | 0.50 |
| | Bradi2g53070 | 0.72 | 0.54 | 0.42 | 0.07 | 0.53 | 0.41 | 0.59 | 0.18 | 0.01 | 0.06 | 0.01 | 0.15 | 0.02 | 0.16 | 0.00 | 0.20 | 0.32 | 0.29 | 0.01 | 0.36 |
| | Bradi3g59300 | 0.42 | 0.60 | 0.45 | 0.04 | 0.01 | 0.32 | 0.55 | 0.25 | 0.48 | 0.40 | 0.01 | 0.37 | 0.67 | 0.72 | 0.20 | 0.57 | 0.56 | 0.49 | 0.44 | 0.21 |
| | Bradi3g15880 | 0.77 | 0.51 | 0.32 | 0.60 | 0.13 | 0.01 | 0.48 | 0.06 | 0.26 | 0.09 | 0.00 | 0.00 | 0.04 | 0.00 | 0.03 | 0.50 | 0.56 | 0.15 | 0.51 | 0.50 |
| | Bradi2g48130 | 0.27 | 0.11 | 0.02 | 0.02 | 0.26 | 0.11 | 0.21 | 0.08 | 0.05 | 0.03 | 0.01 | 0.15 | 0.44 | 0.68 | 0.20 | 0.11 | 0.03 | 0.02 | 0.07 | 0.43 |
| | Bradi1g51045 | 0.77 | 0.60 | 0.32 | 0.60 | 0.55 | 0.57 | 0.72 | 0.18 | 0.19 | 0.18 | 0.70 | 0.30 | 0.44 | 0.31 | 0.23 | 0.35 | 0.06 | 0.49 | 0.54 | 0.50 |
| | Bradi2g18570 | 0.60 | 0.60 | 0.39 | 0.60 | 0.55 | 0.11 | 0.35 | 0.29 | 0.05 | 0.36 | 0.25 | 0.66 | 0.72 | 0.38 | 0.09 | 0.15 | 0.51 | 0.24 | 0.50 | 0.50 |
| | Bradi4g30617 | 0.75 | 0.37 | 0.45 | 0.57 | 0.55 | 0.05 | 0.26 | 0.25 | 0.03 | 0.36 | 0.62 | 0.45 | 0.72 | 0.23 | 0.23 | 0.27 | 0.32 | 0.15 | 0.44 | 0.17 |
| | Bradi2g26987 | 0.60 | 0.60 | 0.41 | 0.46 | 0.13 | 0.47 | 0.43 | 0.32 | 0.48 | 0.18 | 0.13 | 0.59 | 0.67 | 0.51 | 0.20 | 0.08 | 0.11 | 0.06 | 0.07 | 0.13 |
| | Bradi4g43877 | 0.77 | 0.25 | 0.45 | 0.53 | 0.50 | 0.53 | 0.68 | 0.30 | 0.09 | 0.09 | 0.57 | 0.45 | 0.72 | 0.57 | 0.20 | 0.50 | 0.11 | 0.19 | 0.54 | 0.31 |
| | Bradi1g57560 | 0.60 | 0.37 | 0.39 | 0.60 | 0.50 | 0.57 | 0.48 | 0.25 | 0.19 | 0.12 | 0.70 | 0.45 | 0.63 | 0.68 | 0.23 | 0.50 | 0.32 | 0.19 | 0.54 | 0.36 |
| | Bradi1g07290 | 0.75 | 0.19 | 0.32 | 0.55 | 0.39 | 0.23 | 0.26 | 0.25 | 0.19 | 0.24 | 0.68 | 0.45 | 0.36 | 0.65 | 0.23 | 0.44 | 0.40 | 0.24 | 0.44 | 0.43 |
| | Bradi1g72890 | 0.60 | 0.60 | 0.45 | 0.37 | 0.02 | 0.41 | 0.43 | 0.06 | 0.39 | 0.12 | 0.41 | 0.24 | 0.44 | 0.31 | 0.34 | 0.55 | 0.55 | 0.11 | 0.20 | 0.31 |
| | Bradi5g24360 | 0.60 | 0.54 | 0.45 | 0.60 | 0.56 | 0.41 | 0.70 | 0.18 | 0.06 | 0.36 | 0.41 | 0.45 | 0.63 | 0.61 | 0.20 | 0.20 | 0.51 | 0.29 | 0.54 | 0.50 |
| | Bradi2g57747 | 0.60 | 0.25 | 0.21 | 0.58 | 0.50 | 0.41 | 0.30 | 0.28 | 0.06 | 0.12 | 0.50 | 0.37 | 0.23 | 0.57 | 0.23 | 0.35 | 0.16 | 0.03 | 0.44 | 0.50 |
| | Bradi1g64240 | 0.72 | 0.59 | 0.21 | 0.46 | 0.50 | 0.47 | 0.40 | 0.25 | 0.48 | 0.24 | 0.50 | 0.30 | 0.52 | 0.51 | 0.20 | 0.04 | 0.40 | 0.19 | 0.24 | 0.50 |
| | Bradi5g14960 | 0.77 | 0.60 | 0.40 | 0.55 | 0.26 | 0.23 | 0.68 | 0.32 | 0.26 | 0.04 | 0.50 | 0.66 | 0.04 | 0.51 | 0.15 | 0.55 | 0.24 | 0.11 | 0.44 | 0.36 |
| | Bradi1g31337 | 0.27 | 0.25 | 0.11 | 0.55 | 0.39 | 0.05 | 0.43 | 0.06 | 0.41 | 0.18 | 0.50 | 0.59 | 0.52 | 0.57 | 0.15 | 0.55 | 0.56 | 0.42 | 0.40 | 0.10 |
| | Bradi3g48697 | 0.37 | 0.54 | 0.15 | 0.46 | 0.56 | 0.47 | 0.62 | 0.28 | 0.26 | 0.30 | 0.33 | 0.59 | 0.05 | 0.31 | 0.23 | 0.35 | 0.32 | 0.03 | 0.03 | 0.21 |
| DREB | Bradi2g60340 | 0.00 | 0.00 | 0.01 | 0.00 | 0.06 | 0.41 | 0.30 | 0.30 | 0.13 | 0.41 | 0.66 | 0.66 | 0.72 | 0.57 | 0.09 | 0.11 | 0.11 | 0.15 | 0.24 | 0.31 |
| | Bradi2g60331 | 0.00 | 0.00 | 0.02 | 0.00 | 0.02 | 0.53 | 0.21 | 0.08 | 0.02 | 0.01 | 0.18 | 0.09 | 0.00 | 0.51 | 0.34 | 0.27 | 0.40 | 0.09 | 0.03 | 0.36 |
| | Bradi3g57360 | 0.42 | 0.25 | 0.39 | 0.60 | 0.55 | 0.32 | 0.14 | 0.13 | 0.02 | 0.04 | 0.41 | 0.59 | 0.52 | 0.51 | 0.03 | 0.50 | 0.04 | 0.46 | 0.12 | 0.10 |
| | Bradi4g35610 | 0.00 | 0.00 | 0.00 | 0.01 | 0.26 | 0.16 | 0.43 | 0.13 | 0.26 | 0.06 | 0.57 | 0.53 | 0.60 | 0.57 | 0.23 | 0.59 | 0.08 | 0.24 | 0.54 | 0.43 |
| | Bradi4g35570 | 0.00 | 0.00 | 0.00 | 0.00 | 0.26 | 0.60 | 0.59 | 0.35 | 0.13 | 0.09 | 0.41 | 0.30 | 0.44 | 0.70 | 0.03 | 0.35 | 0.16 | 0.24 | 0.35 | 0.50 |
| | Bradi4g35580 | #N/A | #N/A | #N/A | #N/A | #N/A | #N/A | #N/A | #N/A | #N/A | #N/A | #N/A | #N/A | #N/A | #N/A | #N/A | #N/A | #N/A | #N/A | #N/A | #N/A |
| | Bradi4g35600 | 0.00 | 0.00 | 0.00 | 0.00 | 0.00 | 0.11 | 0.35 | 0.06 | 0.33 | 0.30 | 0.33 | 0.30 | 0.60 | 0.71 | 0.09 | 0.60 | 0.56 | 0.29 | 0.12 | 0.43 |
| | Bradi4g35620 | 0.00 | 0.00 | 0.00 | 0.00 | 0.32 | 0.60 | 0.43 | 0.25 | 0.41 | 0.36 | 0.33 | 0.19 | 0.11 | 0.01 | 0.15 | 0.35 | 0.51 | 0.42 | 0.20 | 0.13 |

| | | | | | | | | | | | | | | | | | | | | |
|---|---|---|---|---|---|---|---|---|---|---|---|---|---|---|---|---|---|---|---|---|
| Bradi4g35590 | 0.00 | 0.00 | 0.02 | 0.04 | 0.39 | 0.53 | 0.52 | 0.18 | 0.09 | 0.36 | 0.57 | 0.64 | 0.60 | 0.68 | 0.09 | 0.60 | 0.51 | 0.46 | 0.35 | 0.17 |
| Bradi1g57970 | 0.60 | 0.11 | 0.02 | 0.02 | 0.04 | 0.60 | 0.35 | 0.28 | 0.26 | 0.36 | 0.25 | 0.37 | 0.52 | 0.61 | 0.02 | 0.04 | 0.11 | 0.06 | 0.35 | 0.50 |
| Bradi1g77120 | 0.00 | 0.00 | 0.01 | 0.00 | 0.13 | 0.41 | 0.65 | 0.18 | 0.06 | 0.09 | 0.41 | 0.66 | 0.63 | 0.68 | 0.05 | 0.55 | 0.56 | 0.09 | 0.03 | 0.05 |
| Bradi4g35650 | 0.00 | 0.01 | 0.03 | 0.60 | 0.56 | 0.23 | 0.40 | 0.28 | 0.39 | 0.03 | 0.02 | 0.45 | 0.36 | 0.01 | 0.00 | 0.55 | 0.40 | 0.24 | 0.12 | 0.17 |
| Bradi4g35630 | 0.00 | 0.00 | 0.01 | 0.37 | 0.50 | 0.16 | 0.43 | 0.35 | 0.26 | 0.36 | 0.25 | 0.24 | 0.52 | 0.38 | 0.05 | 0.50 | 0.16 | 0.19 | 0.44 | 0.36 |
| Bradi3g51630 | 0.20 | 0.07 | 0.01 | 0.00 | 0.55 | 0.23 | 0.68 | 0.13 | 0.47 | 0.04 | 0.00 | 0.53 | 0.36 | 0.00 | 0.00 | 0.00 | 0.01 | 0.49 | 0.44 | 0.40 |
| Bradi1g49570 | 0.00 | 0.00 | 0.00 | 0.00 | 0.39 | 0.32 | 0.30 | 0.06 | 0.19 | 0.40 | 0.00 | 0.00 | 0.03 | 0.00 | 0.00 | 0.15 | 0.02 | 0.01 | 0.12 | 0.31 |
| Bradi1g49560 | 0.20 | 0.09 | 0.07 | 0.10 | 0.13 | 0.53 | 0.35 | 0.30 | 0.39 | 0.18 | 0.00 | 0.00 | 0.00 | 0.00 | 0.00 | 0.00 | 0.32 | 0.00 | 0.02 | 0.01 |
| Bradi3g50620 | 0.60 | 0.54 | 0.21 | 0.55 | 0.39 | 0.08 | 0.48 | 0.25 | 0.39 | 0.44 | 0.57 | 0.53 | 0.36 | 0.57 | 0.05 | 0.44 | 0.24 | 0.38 | 0.53 | 0.31 |
| Bradi5g17610 | 0.42 | 0.59 | 0.45 | 0.04 | 0.50 | 0.32 | 0.35 | 0.02 | 0.01 | 0.03 | 0.66 | 0.37 | 0.60 | 0.38 | 0.23 | 0.60 | 0.16 | 0.11 | 0.35 | 0.05 |
| Bradi5g17630 | 0.77 | 0.60 | 0.45 | 0.26 | 0.39 | 0.23 | 0.55 | 0.28 | 0.13 | 0.02 | 0.50 | 0.24 | 0.60 | 0.38 | 0.15 | 0.59 | 0.51 | 0.11 | 0.50 | 0.40 |
| Bradi3g50625 | 0.42 | 0.60 | 0.39 | 0.37 | 0.39 | 0.60 | 0.40 | 0.28 | 0.19 | 0.06 | 0.66 | 0.59 | 0.52 | 0.70 | 0.15 | 0.55 | 0.16 | 0.11 | 0.29 | 0.31 |
| Bradi5g17640 | 0.14 | 0.15 | 0.02 | 0.02 | 0.10 | 0.47 | 0.43 | 0.08 | 0.09 | 0.30 | 0.00 | 0.02 | 0.02 | 0.05 | 0.01 | 0.00 | 0.01 | 0.01 | 0.01 | 0.02 |
| Bradi3g50630 | 0.60 | 0.54 | 0.45 | 0.46 | 0.06 | 0.11 | 0.30 | 0.35 | 0.06 | 0.09 | 0.57 | 0.64 | 0.70 | 0.11 | 0.15 | 0.35 | 0.06 | 0.15 | 0.09 | 0.07 |
| Bradi5g18850 | 0.72 | 0.60 | 0.44 | 0.46 | 0.46 | 0.60 | 0.62 | 0.28 | 0.48 | 0.44 | 0.62 | 0.66 | 0.72 | 0.61 | 0.28 | 0.35 | 0.56 | 0.34 | 0.54 | 0.47 |
| Bradi3g51610 | 0.75 | 0.60 | 0.45 | 0.37 | 0.46 | 0.23 | 0.30 | 0.18 | 0.19 | 0.38 | 0.33 | 0.53 | 0.60 | 0.07 | 0.15 | 0.60 | 0.48 | 0.03 | 0.54 | 0.40 |
| Bradi3g31600 | 0.60 | 0.60 | 0.39 | 0.55 | 0.53 | 0.53 | 0.52 | 0.35 | 0.41 | 0.30 | 0.25 | 0.30 | 0.36 | 0.31 | 0.09 | 0.11 | 0.24 | 0.09 | 0.44 | 0.44 |
| Bradi5g17620 | 0.77 | 0.60 | 0.39 | 0.58 | 0.46 | 0.32 | 0.18 | 0.01 | 0.02 | 0.02 | 0.68 | 0.61 | 0.67 | 0.61 | 0.09 | 0.27 | 0.16 | 0.24 | 0.40 | 0.43 |
| Bradi3g33355 | 0.37 | 0.54 | 0.01 | 0.58 | 0.52 | 0.08 | 0.55 | 0.28 | 0.09 | 0.01 | 0.02 | 0.30 | 0.18 | 0.57 | 0.34 | 0.20 | 0.24 | 0.01 | 0.24 | 0.03 |
| Bradi3g08790 | 0.20 | 0.60 | 0.02 | 0.00 | 0.01 | 0.32 | 0.52 | 0.13 | 0.43 | 0.09 | 0.25 | 0.30 | 0.63 | 0.23 | 0.05 | 0.35 | 0.32 | 0.49 | 0.12 | 0.03 |
| Bradi1g38110 | 0.42 | 0.60 | 0.32 | 0.60 | 0.56 | 0.16 | 0.43 | 0.35 | 0.19 | 0.12 | 0.33 | 0.45 | 0.52 | 0.57 | 0.20 | 0.60 | 0.40 | 0.49 | 0.44 | 0.43 |
| Bradi2g06180 | 0.60 | 0.25 | 0.21 | 0.53 | 0.56 | 0.61 | 0.72 | 0.35 | 0.43 | 0.36 | 0.50 | 0.30 | 0.63 | 0.68 | 0.15 | 0.27 | 0.11 | 0.24 | 0.54 | 0.31 |
| Bradi3g45997 | 0.72 | 0.60 | 0.21 | 0.60 | 0.56 | 0.32 | 0.62 | 0.18 | 0.26 | 0.09 | 0.25 | 0.30 | 0.60 | 0.16 | 0.23 | 0.60 | 0.56 | 0.29 | 0.54 | 0.36 |
| Bradi4g21265 | 0.77 | 0.11 | 0.32 | 0.37 | 0.26 | 0.47 | 0.68 | 0.28 | 0.46 | 0.44 | 0.68 | 0.53 | 0.70 | 0.57 | 0.23 | 0.44 | 0.11 | 0.29 | 0.53 | 0.43 |
| Bradi5g11295 | 0.72 | 0.51 | 0.45 | 0.46 | 0.26 | 0.41 | 0.48 | 0.18 | 0.26 | 0.18 | 0.57 | 0.63 | 0.52 | 0.46 | 0.05 | 0.44 | 0.40 | 0.49 | 0.50 | 0.45 |
| Bradi2g25050 | 0.77 | 0.51 | 0.45 | 0.26 | 0.32 | 0.23 | 0.55 | 0.13 | 0.41 | 0.18 | 0.50 | 0.66 | 0.70 | 0.68 | 0.20 | 0.44 | 0.40 | 0.19 | 0.35 | 0.43 |
| Bradi2g57201 | 0.42 | 0.60 | 0.32 | 0.37 | 0.56 | 0.53 | 0.72 | 0.29 | 0.26 | 0.12 | 0.50 | 0.66 | 0.67 | 0.71 | 0.20 | 0.35 | 0.24 | 0.29 | 0.49 | 0.50 |
| Bradi3g37544 | 0.60 | 0.25 | 0.45 | 0.53 | 0.03 | 0.32 | 0.35 | 0.18 | 0.48 | 0.36 | 0.50 | 0.24 | 0.28 | 0.61 | 0.15 | 0.44 | 0.01 | 0.06 | 0.16 | 0.17 |
| Bradi3g60018 | 0.72 | 0.60 | 0.45 | 0.57 | 0.32 | 0.47 | 0.59 | 0.32 | 0.48 | 0.12 | 0.70 | 0.24 | 0.70 | 0.11 | 0.09 | 0.35 | 0.40 | 0.34 | 0.44 | 0.50 |
| Bradi3g56801 | 0.14 | 0.54 | 0.40 | 0.60 | 0.32 | 0.53 | 0.30 | 0.13 | 0.46 | 0.18 | 0.13 | 0.37 | 0.28 | 0.31 | 0.05 | 0.20 | 0.04 | 0.38 | 0.40 | 0.40 |
| Bradi5g24700 | 0.75 | 0.37 | 0.43 | 0.10 | 0.04 | 0.16 | 0.72 | 0.30 | 0.19 | 0.03 | 0.00 | 0.59 | 0.60 | 0.61 | 0.15 | 0.55 | 0.53 | 0.01 | 0.20 | 0.10 |
| Bradi1g67350 | 0.75 | 0.60 | 0.32 | 0.57 | 0.26 | 0.00 | 0.35 | 0.01 | 0.02 | 0.24 | 0.33 | 0.64 | 0.52 | 0.61 | 0.05 | 0.20 | 0.56 | 0.46 | 0.24 | 0.43 |

| | | | | | | | | | | | | | | | | | | | | | |
|---|---|---|---|---|---|---|---|---|---|---|---|---|---|---|---|---|---|---|---|---|---|
| | Bradi3g57862 | 0.42 | 0.51 | 0.32 | 0.26 | 0.32 | 0.16 | 0.40 | 0.28 | 0.33 | 0.36 | 0.62 | 0.37 | 0.11 | 0.16 | 0.09 | 0.20 | 0.11 | 0.19 | 0.29 | 0.43 |
| | Bradi1g46120 | 0.42 | 0.25 | 0.39 | 0.53 | 0.53 | 0.47 | 0.62 | 0.28 | 0.19 | 0.24 | 0.69 | 0.53 | 0.67 | 0.65 | 0.20 | 0.58 | 0.48 | 0.52 | 0.50 | 0.36 |
| | Bradi1g48320 | 0.42 | 0.54 | 0.32 | 0.37 | 0.19 | 0.41 | 0.72 | 0.08 | 0.41 | 0.24 | 0.02 | 0.09 | 0.36 | 0.01 | 0.09 | 0.02 | 0.08 | 0.00 | 0.01 | 0.01 |
| | Bradi5g24110 | 0.42 | 0.54 | 0.32 | 0.04 | 0.19 | 0.47 | 0.71 | 0.28 | 0.33 | 0.12 | 0.01 | 0.37 | 0.23 | 0.31 | 0.05 | 0.05 | 0.04 | 0.03 | 0.03 | 0.04 |
| | Bradi5g24710 | 0.42 | 0.54 | 0.39 | 0.53 | 0.46 | 0.23 | 0.62 | 0.18 | 0.41 | 0.36 | 0.66 | 0.45 | 0.70 | 0.51 | 0.20 | 0.15 | 0.56 | 0.19 | 0.44 | 0.36 |
| | Bradi3g54160 | 0.72 | 0.59 | 0.39 | 0.46 | 0.56 | 0.47 | 0.55 | 0.35 | 0.41 | 0.24 | 0.25 | 0.45 | 0.44 | 0.31 | 0.34 | 0.55 | 0.40 | 0.50 | 0.49 | 0.50 |
| | Bradi1g47480 | 0.77 | 0.15 | 0.07 | 0.55 | 0.39 | 0.47 | 0.21 | 0.02 | 0.26 | 0.03 | 0.66 | 0.63 | 0.14 | 0.16 | 0.05 | 0.11 | 0.16 | 0.49 | 0.12 | 0.36 |
| | Bradi5g24720 | 0.77 | 0.25 | 0.45 | 0.07 | 0.39 | 0.53 | 0.21 | 0.04 | 0.13 | 0.09 | 0.68 | 0.53 | 0.44 | 0.71 | 0.15 | 0.35 | 0.04 | 0.01 | 0.03 | 0.13 |
| | Bradi1g36590 | 0.77 | 0.60 | 0.32 | 0.55 | 0.46 | 0.23 | 0.26 | 0.04 | 0.02 | 0.04 | 0.57 | 0.30 | 0.05 | 0.00 | 0.26 | 0.55 | 0.32 | 0.02 | 0.01 | 0.01 |
| | Bradi3g07450 | 0.42 | 0.51 | 0.45 | 0.53 | 0.26 | 0.16 | 0.43 | 0.03 | 0.01 | 0.01 | 0.68 | 0.59 | 0.70 | 0.68 | 0.23 | 0.35 | 0.40 | 0.15 | 0.09 | 0.31 |
| | Bradi2g29010 | 0.42 | 0.37 | 0.39 | 0.46 | 0.50 | 0.47 | 0.40 | 0.25 | 0.41 | 0.36 | 0.33 | 0.45 | 0.60 | 0.61 | 0.02 | 0.27 | 0.56 | 0.34 | 0.53 | 0.31 |
| | Bradi1g72990 | 0.60 | 0.60 | 0.45 | 0.57 | 0.53 | 0.41 | 0.65 | 0.13 | 0.26 | 0.18 | 0.62 | 0.59 | 0.60 | 0.65 | 0.15 | 0.27 | 0.55 | 0.34 | 0.50 | 0.50 |
| | Bradi3g12680 | 0.77 | 0.37 | 0.39 | 0.55 | 0.46 | 0.23 | 0.40 | 0.35 | 0.39 | 0.09 | 0.33 | 0.45 | 0.63 | 0.38 | 0.15 | 0.58 | 0.48 | 0.11 | 0.29 | 0.31 |
| | Bradi2g04000 | 0.20 | 0.37 | 0.45 | 0.46 | 0.50 | 0.41 | 0.48 | 0.06 | 0.01 | 0.01 | 0.50 | 0.53 | 0.60 | 0.31 | 0.23 | 0.55 | 0.11 | 0.11 | 0.09 | 0.13 |
| | Bradi2g29960 | 0.20 | 0.04 | 0.45 | 0.37 | 0.04 | 0.00 | 0.18 | 0.01 | 0.02 | 0.01 | 0.18 | 0.03 | 0.01 | 0.00 | 0.00 | 0.07 | 0.01 | 0.00 | 0.01 | 0.03 |
| | Bradi2g15847 | 0.60 | 0.60 | 0.43 | 0.13 | 0.56 | 0.08 | 0.48 | 0.13 | 0.19 | 0.36 | 0.50 | 0.61 | 0.52 | 0.57 | 0.09 | 0.08 | 0.06 | 0.03 | 0.00 | 0.13 |
| | Bradi5g16450 | 0.27 | 0.56 | 0.41 | 0.13 | 0.53 | 0.41 | 0.23 | 0.08 | 0.13 | 0.44 | 0.13 | 0.12 | 0.14 | 0.01 | 0.02 | 0.35 | 0.48 | 0.51 | 0.29 | 0.17 |
| | Bradi3g49810 | 0.77 | 0.37 | 0.45 | 0.46 | 0.39 | 0.01 | 0.23 | 0.00 | 0.01 | 0.01 | 0.00 | 0.00 | 0.00 | 0.00 | 0.00 | 0.00 | 0.16 | 0.00 | 0.00 | 0.03 |
| | Bradi3g35560 | 0.02 | 0.19 | 0.45 | 0.55 | 0.13 | 0.05 | 0.23 | 0.01 | 0.06 | 0.06 | 0.00 | 0.00 | 0.00 | 0.00 | 0.00 | 0.00 | 0.01 | 0.00 | 0.00 | 0.01 |
| | Bradi4g29010 | 0.72 | 0.60 | 0.45 | 0.55 | 0.32 | 0.08 | 0.21 | 0.08 | 0.05 | 0.02 | 0.04 | 0.59 | 0.70 | 0.51 | 0.15 | 0.55 | 0.32 | 0.29 | 0.40 | 0.45 |
| | Bradi1g71740 | 0.75 | 0.55 | 0.39 | 0.19 | 0.13 | 0.02 | 0.26 | 0.32 | 0.13 | 0.44 | 0.00 | 0.00 | 0.00 | 0.00 | 0.00 | 0.00 | 0.16 | 0.01 | 0.03 | 0.31 |
| | Bradi3g24000 | 0.60 | 0.51 | 0.21 | 0.26 | 0.39 | 0.47 | 0.35 | 0.35 | 0.05 | 0.43 | 0.18 | 0.24 | 0.63 | 0.71 | 0.15 | 0.58 | 0.24 | 0.06 | 0.16 | 0.21 |
| | Bradi3g58980 | 0.42 | 0.60 | 0.45 | 0.05 | 0.04 | 0.00 | 0.23 | 0.02 | 0.00 | 0.01 | 0.00 | 0.02 | 0.00 | 0.00 | 0.00 | 0.20 | 0.24 | 0.02 | 0.02 | 0.03 |
| | Bradi1g45470 | 0.72 | 0.54 | 0.32 | 0.13 | 0.32 | 0.32 | 0.26 | 0.13 | 0.44 | 0.44 | 0.00 | 0.01 | 0.00 | 0.00 | 0.00 | 0.02 | 0.08 | 0.01 | 0.00 | 0.04 |
| | Bradi2g22505 | 0.60 | 0.55 | 0.45 | 0.19 | 0.32 | 0.32 | 0.59 | 0.35 | 0.13 | 0.43 | 0.62 | 0.66 | 0.72 | 0.70 | 0.34 | 0.60 | 0.32 | 0.03 | 0.49 | 0.43 |
| ERF | Bradi1g53756 | 0.77 | 0.60 | 0.45 | 0.46 | 0.55 | 0.53 | 0.30 | 0.35 | 0.48 | 0.12 | 0.50 | 0.61 | 0.11 | 0.46 | 0.05 | 0.50 | 0.24 | 0.42 | 0.54 | 0.43 |
| | Bradi1g53753 | 0.77 | 0.37 | 0.09 | 0.60 | 0.26 | 0.53 | 0.70 | 0.13 | 0.43 | 0.44 | 0.50 | 0.37 | 0.63 | 0.38 | 0.34 | 0.60 | 0.32 | 0.29 | 0.29 | 0.43 |
| | Bradi1g23756 | 0.42 | 0.54 | 0.21 | 0.19 | 0.04 | 0.11 | 0.72 | 0.32 | 0.33 | 0.44 | 0.57 | 0.61 | 0.28 | 0.51 | 0.23 | 0.60 | 0.48 | 0.24 | 0.54 | 0.36 |
| | Bradi1g54450 | 0.60 | 0.54 | 0.39 | 0.26 | 0.55 | 0.11 | 0.23 | 0.02 | 0.01 | 0.01 | 0.50 | 0.66 | 0.44 | 0.61 | 0.05 | 0.15 | 0.48 | 0.00 | 0.00 | 0.00 |
| | Bradi1g38238 | 0.60 | 0.56 | 0.32 | 0.60 | 0.19 | 0.11 | 0.21 | 0.02 | 0.03 | 0.01 | 0.57 | 0.61 | 0.67 | 0.57 | 0.23 | 0.55 | 0.32 | 0.11 | 0.12 | 0.17 |
| | Bradi4g03371 | 0.42 | 0.37 | 0.21 | 0.60 | 0.46 | 0.23 | 0.72 | 0.35 | 0.41 | 0.12 | 0.57 | 0.45 | 0.63 | 0.23 | 0.09 | 0.44 | 0.32 | 0.34 | 0.44 | 0.50 |
| | Bradi4g35655 | 0.60 | 0.54 | 0.45 | 0.60 | 0.13 | 0.61 | 0.69 | 0.08 | 0.05 | 0.03 | 0.50 | 0.53 | 0.67 | 0.57 | 0.23 | 0.50 | 0.53 | 0.24 | 0.09 | 0.13 |

| | | | | | | | | | | | | | | | | | | | | |
|---|---|---|---|---|---|---|---|---|---|---|---|---|---|---|---|---|---|---|---|---|
| Bradi2g24175 | 0.72 | 0.60 | 0.39 | 0.37 | 0.39 | 0.08 | 0.69 | 0.28 | 0.05 | 0.36 | 0.00 | 0.01 | 0.00 | 0.00 | 0.00 | 0.01 | 0.00 | 0.00 | 0.01 | 0.05 |
| Bradi2g56145 | 0.42 | 0.60 | 0.45 | 0.04 | 0.06 | 0.16 | 0.21 | 0.34 | 0.19 | 0.24 | 0.33 | 0.02 | 0.03 | 0.01 | 0.00 | 0.01 | 0.00 | 0.00 | 0.00 | 0.00 |
| Bradi4g41616 | 0.75 | 0.37 | 0.39 | 0.19 | 0.02 | 0.47 | 0.40 | 0.30 | 0.05 | 0.01 | 0.00 | 0.00 | 0.00 | 0.00 | 0.00 | 0.00 | 0.00 | 0.00 | 0.00 | 0.00 |
| Bradi5g08380 | 0.72 | 0.19 | 0.11 | 0.13 | 0.07 | 0.16 | 0.62 | 0.18 | 0.02 | 0.01 | 0.69 | 0.53 | 0.60 | 0.46 | 0.15 | 0.01 | 0.00 | 0.00 | 0.00 | 0.00 |
| Bradi3g58015 | 0.37 | 0.05 | 0.05 | 0.00 | 0.26 | 0.23 | 0.21 | 0.30 | 0.48 | 0.18 | 0.00 | 0.00 | 0.00 | 0.00 | 0.00 | 0.00 | 0.01 | 0.00 | 0.00 | 0.00 |
| Bradi3g38140 | 0.07 | 0.15 | 0.07 | 0.37 | 0.55 | 0.32 | 0.65 | 0.28 | 0.33 | 0.43 | 0.08 | 0.24 | 0.11 | 0.31 | 0.15 | 0.02 | 0.11 | 0.19 | 0.29 | 0.36 |
| Bradi4g27850 | 0.60 | 0.51 | 0.32 | 0.60 | 0.52 | 0.53 | 0.59 | 0.35 | 0.13 | 0.18 | 0.68 | 0.37 | 0.44 | 0.31 | 0.34 | 0.55 | 0.48 | 0.29 | 0.12 | 0.50 |
| Bradi3g40343 | 0.60 | 0.51 | 0.21 | 0.46 | 0.04 | 0.53 | 0.65 | 0.04 | 0.33 | 0.18 | 0.66 | 0.45 | 0.63 | 0.65 | 0.09 | 0.58 | 0.32 | 0.24 | 0.35 | 0.43 |
| Bradi2g02100 | 0.72 | 0.51 | 0.39 | 0.13 | 0.13 | 0.47 | 0.30 | 0.25 | 0.09 | 0.01 | 0.18 | 0.15 | 0.36 | 0.38 | 0.23 | 0.20 | 0.16 | 0.38 | 0.35 | 0.50 |
| Bradi3g60120 | 0.42 | 0.56 | 0.15 | 0.13 | 0.39 | 0.02 | 0.23 | 0.02 | 0.05 | 0.01 | 0.18 | 0.59 | 0.08 | 0.46 | 0.15 | 0.27 | 0.40 | 0.05 | 0.35 | 0.26 |
| Bradi4g31040 | 0.77 | 0.60 | 0.44 | 0.03 | 0.03 | 0.32 | 0.40 | 0.29 | 0.09 | 0.12 | 0.70 | 0.45 | 0.60 | 0.16 | 0.15 | 0.50 | 0.24 | 0.09 | 0.29 | 0.50 |
| Bradi1g46690 | 0.60 | 0.56 | 0.45 | 0.13 | 0.02 | 0.53 | 0.68 | 0.35 | 0.13 | 0.38 | 0.57 | 0.30 | 0.60 | 0.46 | 0.15 | 0.15 | 0.32 | 0.11 | 0.16 | 0.40 |
| Bradi1g72457 | #N/A | #N/A | #N/A | #N/A | #N/A | #N/A | #N/A | #N/A | #N/A | #N/A | #N/A | #N/A | #N/A | #N/A | #N/A | #N/A | #N/A | #N/A | #N/A | #N/A |
| Bradi2g27920 | 0.20 | 0.60 | 0.39 | 0.60 | 0.32 | 0.16 | 0.52 | 0.35 | 0.13 | 0.40 | 0.41 | 0.61 | 0.70 | 0.61 | 0.20 | 0.44 | 0.32 | 0.24 | 0.35 | 0.40 |
| Bradi2g11890 | 0.72 | 0.60 | 0.09 | 0.57 | 0.32 | 0.01 | 0.23 | 0.29 | 0.26 | 0.18 | 0.33 | 0.45 | 0.72 | 0.57 | 0.05 | 0.07 | 0.06 | 0.01 | 0.05 | 0.05 |
| Bradi1g17961 | 0.77 | 0.54 | 0.45 | 0.55 | 0.56 | 0.60 | 0.55 | 0.35 | 0.26 | 0.30 | 0.50 | 0.45 | 0.70 | 0.70 | 0.09 | 0.27 | 0.56 | 0.42 | 0.54 | 0.36 |
| Bradi5g17480 | 0.60 | 0.54 | 0.45 | 0.04 | 0.02 | 0.02 | 0.30 | 0.35 | 0.19 | 0.02 | 0.06 | 0.05 | 0.52 | 0.00 | 0.00 | 0.60 | 0.55 | 0.46 | 0.20 | 0.04 |
| Bradi3g50490 | 0.00 | 0.01 | 0.45 | 0.46 | 0.03 | 0.00 | 0.14 | 0.04 | 0.01 | 0.00 | 0.00 | 0.24 | 0.05 | 0.07 | 0.03 | 0.01 | 0.00 | 0.00 | 0.00 | 0.43 |
| Bradi3g15084 | 0.42 | 0.25 | 0.45 | 0.37 | 0.39 | 0.32 | 0.40 | 0.32 | 0.33 | 0.24 | 0.68 | 0.37 | 0.28 | 0.38 | 0.23 | 0.20 | 0.53 | 0.42 | 0.29 | 0.47 |
| Bradi1g00666 | 0.60 | 0.60 | 0.09 | 0.46 | 0.32 | 0.47 | 0.70 | 0.28 | 0.09 | 0.36 | 0.57 | 0.63 | 0.60 | 0.70 | 0.34 | 0.44 | 0.40 | 0.24 | 0.20 | 0.43 |
| Bradi3g12566 | 0.77 | 0.51 | 0.21 | 0.53 | 0.46 | 0.23 | 0.30 | 0.06 | 0.09 | 0.09 | 0.57 | 0.66 | 0.52 | 0.61 | 0.23 | 0.20 | 0.32 | 0.46 | 0.40 | 0.21 |
| Bradi3g12567 | 0.77 | 0.55 | 0.45 | 0.46 | 0.19 | 0.32 | 0.26 | 0.35 | 0.41 | 0.41 | 0.69 | 0.53 | 0.36 | 0.65 | 0.03 | 0.27 | 0.48 | 0.19 | 0.54 | 0.50 |
| Bradi4g38941 | 0.42 | 0.56 | 0.45 | 0.37 | 0.32 | 0.41 | 0.35 | 0.28 | 0.26 | 0.24 | 0.33 | 0.66 | 0.60 | 0.01 | 0.15 | 0.55 | 0.40 | 0.49 | 0.40 | 0.21 |
| Bradi3g12565 | 0.42 | 0.60 | 0.45 | 0.55 | 0.32 | 0.23 | 0.35 | 0.29 | 0.39 | 0.44 | 0.25 | 0.61 | 0.52 | 0.31 | 0.09 | 0.55 | 0.56 | 0.19 | 0.54 | 0.43 |
| Bradi3g27690 | 0.42 | 0.25 | 0.15 | 0.46 | 0.56 | 0.32 | 0.48 | 0.06 | 0.26 | 0.30 | 0.25 | 0.37 | 0.60 | 0.11 | 0.09 | 0.35 | 0.11 | 0.00 | 0.20 | 0.50 |
| Bradi1g75040 | 0.07 | 0.37 | 0.21 | 0.00 | 0.00 | 0.11 | 0.72 | 0.25 | 0.19 | 0.18 | 0.41 | 0.37 | 0.44 | 0.68 | 0.23 | 0.50 | 0.40 | 0.46 | 0.40 | 0.40 |
| Bradi3g50508 | 0.60 | 0.37 | 0.21 | 0.55 | 0.13 | 0.16 | 0.59 | 0.35 | 0.13 | 0.18 | 0.57 | 0.59 | 0.60 | 0.38 | 0.15 | 0.04 | 0.56 | 0.09 | 0.02 | 0.10 |
| Bradi5g17490 | 0.42 | 0.54 | 0.45 | 0.58 | 0.19 | 0.02 | 0.30 | 0.02 | 0.06 | 0.30 | 0.62 | 0.59 | 0.44 | 0.51 | 0.05 | 0.55 | 0.56 | 0.05 | 0.24 | 0.50 |
| Bradi5g25570 | 0.00 | 0.00 | 0.02 | 0.46 | 0.50 | 0.41 | 0.26 | 0.35 | 0.43 | 0.18 | 0.00 | 0.00 | 0.00 | 0.00 | 0.00 | 0.01 | 0.48 | 0.00 | 0.03 | 0.01 |
| Bradi2g16442 | 0.60 | 0.15 | 0.39 | 0.58 | 0.50 | 0.47 | 0.71 | 0.08 | 0.13 | 0.09 | 0.70 | 0.45 | 0.52 | 0.51 | 0.23 | 0.50 | 0.40 | 0.38 | 0.49 | 0.26 |
| Bradi2g07357 | 0.72 | 0.60 | 0.21 | 0.55 | 0.55 | 0.47 | 0.55 | 0.35 | 0.41 | 0.36 | 0.66 | 0.45 | 0.63 | 0.38 | 0.23 | 0.59 | 0.40 | 0.42 | 0.29 | 0.43 |
| Bradi2g45530 | 0.60 | 0.54 | 0.21 | 0.46 | 0.39 | 0.02 | 0.40 | 0.18 | 0.26 | 0.24 | 0.41 | 0.66 | 0.70 | 0.11 | 0.05 | 0.50 | 0.51 | 0.29 | 0.50 | 0.31 |

| | | | | | | | | | | | | | | | | | | | | |
|---|---|---|---|---|---|---|---|---|---|---|---|---|---|---|---|---|---|---|---|---|
| Bradi1g48625 | 0.77 | 0.60 | 0.45 | 0.55 | 0.02 | 0.05 | 0.40 | 0.13 | 0.13 | 0.36 | 0.41 | 0.61 | 0.70 | 0.65 | 0.23 | 0.08 | 0.51 | 0.38 | 0.44 | 0.44 |
| Bradi2g31480 | 0.20 | 0.54 | 0.45 | 0.07 | 0.06 | 0.02 | 0.16 | 0.01 | 0.00 | 0.00 | 0.66 | 0.15 | 0.44 | 0.71 | 0.34 | 0.03 | 0.06 | 0.42 | 0.35 | 0.47 |
| Bradi2g50035 | 0.14 | 0.60 | 0.42 | 0.46 | 0.39 | 0.41 | 0.59 | 0.28 | 0.39 | 0.02 | 0.33 | 0.64 | 0.14 | 0.03 | 0.09 | 0.11 | 0.03 | 0.46 | 0.50 | 0.02 |
| Bradi1g04110 | 0.42 | 0.54 | 0.39 | 0.53 | 0.32 | 0.61 | 0.59 | 0.13 | 0.13 | 0.12 | 0.25 | 0.45 | 0.60 | 0.31 | 0.09 | 0.57 | 0.16 | 0.24 | 0.24 | 0.13 |
| Bradi3g47610 | 0.77 | 0.60 | 0.45 | 0.37 | 0.56 | 0.11 | 0.18 | 0.13 | 0.26 | 0.24 | 0.33 | 0.59 | 0.36 | 0.57 | 0.20 | 0.11 | 0.51 | 0.29 | 0.53 | 0.36 |
| Bradi2g21060 | 0.00 | 0.00 | 0.01 | 0.02 | 0.26 | 0.53 | 0.23 | 0.02 | 0.02 | 0.09 | 0.00 | 0.00 | 0.00 | 0.00 | 0.00 | 0.00 | 0.40 | 0.00 | 0.01 | 0.01 |
| Bradi2g52370 | 0.00 | 0.00 | 0.01 | 0.00 | 0.32 | 0.11 | 0.52 | 0.35 | 0.41 | 0.12 | 0.00 | 0.06 | 0.63 | 0.00 | 0.01 | 0.50 | 0.04 | 0.15 | 0.04 | 0.36 |
| Bradi3g06562 | 0.72 | 0.37 | 0.21 | 0.37 | 0.46 | 0.53 | 0.72 | 0.29 | 0.26 | 0.24 | 0.41 | 0.09 | 0.08 | 0.07 | 0.05 | 0.03 | 0.04 | 0.03 | 0.05 | 0.36 |
| Bradi5g21250 | 0.00 | 0.00 | 0.00 | 0.00 | 0.01 | 0.41 | 0.43 | 0.06 | 0.33 | 0.24 | 0.00 | 0.00 | 0.00 | 0.00 | 0.00 | 0.00 | 0.56 | 0.00 | 0.00 | 0.01 |
| Bradi3g18070 | 0.07 | 0.15 | 0.45 | 0.55 | 0.56 | 0.08 | 0.23 | 0.01 | 0.26 | 0.06 | 0.00 | 0.00 | 0.00 | 0.00 | 0.00 | 0.00 | 0.02 | 0.00 | 0.03 | 0.10 |
| Bradi3g18073 | 0.77 | 0.54 | 0.32 | 0.13 | 0.26 | 0.47 | 0.20 | 0.02 | 0.02 | 0.30 | 0.25 | 0.45 | 0.67 | 0.03 | 0.20 | 0.44 | 0.51 | 0.02 | 0.51 | 0.21 |
| Bradi5g08471 | 0.72 | 0.51 | 0.15 | 0.58 | 0.46 | 0.57 | 0.59 | 0.18 | 0.26 | 0.18 | 0.68 | 0.45 | 0.52 | 0.31 | 0.09 | 0.57 | 0.51 | 0.34 | 0.54 | 0.47 |
| Bradi3g44470 | 0.77 | 0.15 | 0.39 | 0.53 | 0.50 | 0.11 | 0.16 | 0.06 | 0.06 | 0.30 | 0.66 | 0.45 | 0.60 | 0.65 | 0.23 | 0.08 | 0.55 | 0.09 | 0.54 | 0.26 |
| Bradi1g18580 | 0.42 | 0.56 | 0.41 | 0.55 | 0.06 | 0.32 | 0.43 | 0.28 | 0.48 | 0.18 | 0.25 | 0.53 | 0.36 | 0.38 | 0.34 | 0.60 | 0.56 | 0.49 | 0.24 | 0.40 |
| Bradi1g33550 | 0.60 | 0.37 | 0.45 | 0.53 | 0.32 | 0.16 | 0.16 | 0.04 | 0.05 | 0.06 | 0.25 | 0.59 | 0.67 | 0.71 | 0.34 | 0.08 | 0.24 | 0.42 | 0.51 | 0.36 |
| Bradi3g04410 | 0.42 | 0.51 | 0.45 | 0.13 | 0.19 | 0.53 | 0.55 | 0.25 | 0.19 | 0.30 | 0.41 | 0.45 | 0.70 | 0.38 | 0.20 | 0.20 | 0.32 | 0.11 | 0.49 | 0.44 |
| Bradi2g21067 | 0.10 | 0.09 | 0.02 | 0.05 | 0.07 | 0.47 | 0.43 | 0.08 | 0.41 | 0.04 | 0.00 | 0.66 | 0.63 | 0.65 | 0.05 | 0.57 | 0.08 | 0.46 | 0.12 | 0.03 |
| Bradi2g52380 | 0.60 | 0.37 | 0.41 | 0.60 | 0.32 | 0.08 | 0.35 | 0.28 | 0.33 | 0.44 | 0.25 | 0.37 | 0.70 | 0.31 | 0.03 | 0.15 | 0.51 | 0.19 | 0.35 | 0.46 |

Footnote:

1. $Log_2$ based fold changes of expression profiles of BdAP2/EREBP genes using the Affymetrix Brachypodium Genome Array (BradiAR1b520742) under four abiotic stress conditions of cold, heat, drought and salt. #N/A represents that did not be detected in the microarray data.
2. If the q value was less than 0.05, it was marked in red.
3. If the $log_2$ based fold change of treatment/control at each time point under the corresponding treatment was less than -1, it was marked in green backgroud.
4. If the $log_2$ based fold change of treatment/control at each time point under the corresponding treatment was greater than 1, it was marked in orange backgroud.

Supplementary Table 7:

The expression profiles (Log2-based fold change and the corresponding q value ) of wheat DREB/ERF under three abiotic stress conditions.

| | | $Log_2$ based fold change (logFC) and q values of treatment/control at each time point under the corresponding treatment | | | | | | | | | | | | | | | | | | | |
|---|---|---|---|---|---|---|---|---|---|---|---|---|---|---|---|---|---|---|---|---|---|
| sub_ family | gene | cold 2 d | | cold 14 d | | cold 21 d | | cold 35 d | | cold 42 d | | cold 56 d | | cold70 d | | drought 48 h | | drought 72h | | heat 1 h | |
| | | q | logFC | q | logFC | q | logFC | q | logFC | q | logFC | q | logFC | q | logFC | q | logFC | q | logFC | q | logFC |
| DREB | Ta.41731 | 0.02 | 2.23 | 0.01 | 1.51 | 0.04 | 1.36 | 0.29 | 0.48 | 0.99 | 0.01 | 0.19 | -0.55 | 0.19 | -0.50 | 1.00 | 0.05 | 1.00 | 0.10 | 0.62 | -0.41 |
| | Ta.41203 | 0.03 | 1.61 | 0.13 | 1.01 | 0.10 | 1.08 | 0.15 | 0.94 | 0.06 | 1.34 | 0.38 | 0.59 | 0.88 | 0.12 | 1.00 | -0.03 | 1.00 | 0.16 | 0.16 | 0.77 |
| | Ta.62077 | 0.07 | 1.19 | 0.28 | 0.48 | 0.07 | 0.86 | 0.16 | 0.62 | 0.12 | 0.69 | 0.51 | 0.31 | 0.86 | -0.10 | 1.00 | 0.11 | 1.00 | 0.28 | 0.76 | 0.12 |
| | Ta.49353 | 0.92 | -0.02 | 0.42 | 0.18 | 0.94 | 0.01 | 0.65 | -0.09 | 0.76 | -0.07 | 0.99 | 0.00 | 0.19 | -0.23 | 1.00 | -0.04 | 1.00 | -0.23 | 0.01 | -1.43 |
| | Ta.30516 | 0.23 | 0.19 | 0.91 | -0.02 | 0.39 | 0.10 | 0.94 | -0.02 | 0.77 | -0.06 | 0.41 | 0.15 | 0.89 | -0.03 | 1.00 | 0.32 | 1.00 | -0.20 | 0.32 | 0.96 |
| | Ta.30495 | 0.21 | 1.08 | 0.34 | 0.80 | 0.13 | 1.32 | 0.13 | 1.27 | 0.17 | 1.28 | 0.44 | 0.68 | 0.80 | -0.26 | 1.00 | 0.25 | 1.00 | 0.10 | 0.97 | -0.03 |
| | Ta.34828 | 0.10 | 1.11 | 0.02 | 1.30 | 0.02 | 1.18 | 0.11 | 0.83 | 0.25 | 0.51 | 0.71 | -0.18 | 0.23 | -0.54 | 1.00 | 0.00 | 1.00 | 0.66 | 0.86 | 0.21 |
| | Ta.5364 | 0.06 | 0.53 | 0.07 | 0.41 | 0.00 | 0.65 | 0.09 | 0.37 | 0.28 | 0.17 | 0.91 | 0.04 | 0.06 | -0.36 | 1.00 | 0.03 | 1.00 | 0.34 | 0.01 | -1.30 |
| | Ta.48248 | 0.23 | 0.15 | 0.95 | -0.01 | 0.38 | 0.12 | 0.78 | 0.05 | 0.97 | 0.01 | 0.76 | 0.06 | 0.87 | -0.05 | 1.00 | 0.08 | 1.00 | 0.46 | 1.00 | 0.00 |
| | Ta.13471 | 0.05 | -0.91 | 0.01 | -1.39 | 0.02 | -0.99 | 0.01 | -1.39 | 0.01 | -1.42 | 0.01 | -1.35 | 0.01 | -1.43 | 1.00 | -0.07 | 1.00 | 0.06 | 0.24 | -1.77 |
| | Ta.45422 | 0.06 | 1.04 | 0.05 | 0.59 | 0.03 | 0.77 | 0.00 | 0.71 | 0.01 | 0.75 | 0.02 | 0.71 | 0.45 | 0.19 | 1.00 | 0.05 | 1.00 | 0.38 | 0.05 | 0.95 |
| | Ta.50434 | 0.65 | -0.18 | 0.40 | -0.36 | 0.41 | -0.30 | 0.44 | -0.30 | 0.29 | -0.44 | 0.49 | -0.30 | 0.19 | -0.51 | 1.00 | 0.01 | 1.00 | 0.22 | 0.98 | 0.01 |
| | Ta.30470 | 0.25 | 0.22 | 0.86 | 0.04 | 0.32 | 0.20 | 0.82 | -0.05 | 0.50 | 0.13 | 0.90 | 0.03 | 0.91 | -0.05 | 1.00 | 0.12 | 1.00 | 0.19 | 0.58 | -0.80 |
| | Ta.33936 | 0.95 | 0.01 | 0.28 | 0.16 | 0.80 | -0.03 | 0.69 | 0.08 | 0.79 | 0.05 | 0.94 | -0.02 | 0.75 | 0.06 | 1.00 | 0.04 | 1.00 | -0.09 | 0.08 | 2.33 |
| | Ta.920 | 0.03 | -1.27 | 0.12 | -0.92 | 0.08 | -0.96 | 0.09 | -0.99 | 0.15 | -0.89 | 0.26 | -0.68 | 0.71 | -0.23 | 1.00 | 0.07 | 1.00 | -0.12 | 0.01 | -0.89 |
| | Ta.57800 | 0.04 | -1.01 | 0.09 | -0.83 | 0.04 | -0.94 | 0.07 | -0.91 | 0.21 | -0.63 | 0.26 | -0.57 | 0.71 | -0.19 | 1.00 | -0.26 | 1.00 | 0.18 | 0.91 | -0.02 |
| | Ta.5304 | 0.57 | 0.09 | 0.80 | -0.06 | 0.49 | 0.10 | 0.97 | -0.01 | 0.70 | 0.08 | 0.56 | 0.15 | 0.52 | 0.16 | 1.00 | -0.03 | 1.00 | 0.04 | 0.39 | -1.05 |
| | Ta.5255 | 0.05 | -0.37 | 0.09 | -0.31 | 0.05 | -0.34 | 0.13 | -0.34 | 0.58 | -0.12 | 0.80 | -0.06 | 0.34 | 0.18 | 1.00 | -0.42 | 1.00 | 0.09 | 0.08 | 0.38 |
| | Ta.26980 | 0.27 | -0.14 | 0.19 | -0.20 | 0.53 | -0.10 | 0.73 | -0.05 | 0.86 | -0.03 | 0.80 | -0.05 | 0.66 | 0.09 | 1.00 | -0.32 | 1.00 | -0.05 | 0.38 | -0.27 |
| | Ta.58074 | 0.73 | -0.11 | 0.27 | -0.34 | 0.42 | -0.21 | 0.33 | -0.29 | 0.26 | -0.35 | 0.45 | -0.24 | 0.77 | -0.10 | 1.00 | -0.17 | 1.00 | 0.56 | 0.82 | 0.07 |
| ERF | Ta.41713 | 0.13 | -0.86 | 0.02 | -1.47 | 0.03 | -1.15 | 0.01 | -1.65 | 0.02 | -1.45 | 0.02 | -1.66 | 0.05 | -1.08 | 1.00 | 0.15 | 1.00 | -0.29 | 0.02 | -0.96 |
| | Ta.14000 | 0.09 | -0.56 | 0.00 | -0.93 | 0.00 | -0.75 | 0.01 | -0.93 | 0.00 | -0.88 | 0.00 | -1.09 | 0.03 | -0.62 | 1.00 | 0.22 | 1.00 | -0.33 | 0.01 | 0.84 |
| | Ta.17378 | 0.06 | -1.24 | 0.00 | -2.93 | 0.00 | -1.98 | 0.01 | -2.46 | 0.00 | -2.52 | 0.00 | -2.44 | 0.01 | -0.87 | 1.00 | -0.30 | 1.00 | 0.01 | 0.82 | -0.06 |
| | Ta.18870 | 0.70 | -0.07 | 0.96 | 0.01 | 0.44 | 0.16 | 0.45 | 0.16 | 0.61 | 0.12 | 0.84 | 0.06 | 0.48 | 0.30 | 1.00 | 0.03 | 1.00 | 1.29 | 0.15 | 0.50 |
| | Ta.14074 | 0.61 | 0.11 | 0.82 | 0.07 | 0.53 | 0.15 | 0.97 | 0.01 | 0.47 | 0.20 | 0.65 | 0.15 | 0.29 | 0.35 | 1.00 | 0.00 | 1.00 | 0.24 | 0.07 | 0.73 |
| | Ta.58355 | 0.07 | -0.98 | 0.18 | -0.69 | 0.13 | -0.67 | 0.15 | -0.68 | 0.73 | -0.26 | 0.41 | -0.43 | 0.39 | -0.47 | 1.00 | -0.02 | 1.00 | 0.71 | 0.05 | -0.81 |
| | Ta.8281 | 0.03 | -0.92 | 0.28 | -0.49 | 0.19 | -0.49 | 0.21 | -0.52 | 0.86 | 0.19 | 0.64 | -0.23 | 0.81 | 0.21 | 1.00 | -0.28 | 1.00 | 0.17 | 0.07 | -0.43 |
| | Ta.24059 | 0.04 | -1.29 | 0.03 | -1.32 | 0.02 | -1.41 | 0.04 | -1.26 | 0.13 | -0.89 | 0.11 | -0.98 | 0.64 | -0.30 | 1.00 | 0.12 | 1.00 | 0.13 | 0.04 | 0.56 |
| | Ta.57703 | 0.03 | -0.56 | 0.01 | 0.92 | 0.17 | 0.31 | 0.01 | 0.60 | 0.01 | 1.22 | 0.00 | 0.90 | 0.05 | 0.68 | 1.00 | 0.26 | 1.00 | 0.15 | 0.27 | -0.85 |
| | Ta.59916 | 0.33 | -0.18 | 0.01 | 0.51 | 0.03 | 0.49 | 0.01 | 0.60 | 0.02 | 0.85 | 0.09 | 0.31 | 0.52 | 0.14 | 1.00 | -0.01 | 1.00 | -0.21 | 0.10 | -1.70 |
| | Ta.27144 | 0.03 | -0.65 | 0.10 | 0.62 | 0.13 | 0.45 | 0.01 | 0.95 | 0.00 | 1.57 | 0.00 | 1.42 | 0.00 | 1.27 | 1.00 | 0.57 | 1.00 | -0.38 | 0.93 | 0.04 |
| | Ta.16395 | 0.61 | -0.07 | 0.51 | -0.10 | 0.83 | 0.03 | 0.74 | 0.05 | 0.78 | -0.07 | 0.38 | 0.15 | 0.79 | 0.07 | 1.00 | -0.11 | 1.00 | 0.04 | 0.32 | 0.75 |
| | Ta.59500 | 0.63 | 0.08 | 0.33 | 0.19 | 0.46 | 0.12 | 0.25 | 0.22 | 0.95 | 0.02 | 0.41 | 0.20 | 0.89 | 0.04 | 1.00 | -0.08 | 1.00 | 0.01 | 0.09 | 1.68 |
| | Ta.33090 | 0.00 | -1.85 | 0.00 | -1.71 | 0.00 | -1.84 | 0.00 | -1.99 | 0.01 | -1.20 | 0.00 | -1.50 | 0.05 | -0.91 | 1.00 | -0.12 | 1.00 | -0.05 | 0.00 | -1.61 |
| | Ta.27555 | 0.89 | 0.03 | 0.94 | -0.02 | 0.76 | 0.05 | 0.93 | -0.02 | 0.58 | 0.15 | 0.35 | 0.20 | 0.80 | 0.07 | 1.00 | 0.03 | 1.00 | -0.11 | 0.36 | -1.48 |
| | Ta.44587 | 0.89 | -0.03 | 0.60 | 0.12 | 0.03 | 0.39 | 0.92 | 0.03 | 0.02 | 0.56 | 0.01 | 0.85 | 0.00 | 1.42 | 1.00 | -0.61 | 1.00 | -0.03 | 0.08 | -1.93 |
| | Ta.25231 | 0.59 | -0.06 | 0.38 | -0.14 | 0.94 | -0.01 | 0.15 | -0.23 | 0.69 | -0.08 | 0.38 | -0.14 | 0.74 | -0.06 | 1.00 | 0.29 | 1.00 | 0.32 | 0.44 | -0.68 |
| | Ta.4470 | 0.27 | -1.33 | 0.21 | -1.69 | 0.21 | -1.53 | 0.22 | -1.59 | 0.20 | -1.73 | 0.20 | -1.77 | 0.26 | -1.48 | 1.00 | -0.13 | 1.00 | -0.12 | 0.95 | -0.08 |
| | Ta.48436 | 0.29 | 0.12 | 0.12 | -0.20 | 0.08 | -0.21 | 0.06 | -0.30 | 0.97 | -0.01 | 0.45 | -0.13 | 0.93 | 0.02 | 1.00 | -0.08 | 1.00 | -0.13 | 0.01 | -0.91 |
| | Ta.35532 | 0.94 | 0.02 | 0.78 | -0.08 | 0.92 | 0.02 | 0.86 | -0.06 | 0.25 | -0.27 | 0.59 | -0.14 | 0.13 | -0.33 | 1.00 | -0.25 | 1.00 | 0.88 | 0.00 | 1.39 |
| | Ta.31753 | 0.32 | 0.51 | 0.11 | 0.78 | 0.09 | 0.79 | 0.11 | 0.94 | 0.01 | 1.42 | 0.10 | 0.81 | 0.56 | 0.35 | 1.00 | 0.12 | 1.00 | 0.16 | 0.53 | -0.39 |
| | Ta.44806 | 0.25 | 0.20 | 0.22 | 0.20 | 0.19 | 0.18 | 0.34 | 0.14 | 0.81 | 0.05 | 0.78 | 0.06 | 0.66 | -0.08 | 1.00 | -0.04 | 1.00 | 0.31 | 0.18 | -0.63 |

Footnote: 1. LogFC of expression profiles of TaDREB/ERF genes from Gene Expression Omnibus (dataset ID: GSE23889, Winter Manitou; GSE70443; GSE60351 ) under cold, drought and heat stresses.
2. If the q value was less than 0.05, it was marked in pink.
3. If the LogFC treatment/control at each time point under the corresponding treatment was less than -1, it was marked in green backgroud.
4. If the log2 based fold change of treatment/control at each time point under the corresponding treatment was greater than 1, it was marked in orange backgroud.

Supplementary Table 8:

The expression profiles ($Log_2$_based fold change and the corresponding q value ) of rice DREB/ERF under three abiotic stress conditions.

| sub_ family | gene | cold 3 h | | drought 3 h | | salt 3 h | |
|---|---|---|---|---|---|---|---|
| | | q | logFC | q | logFC | q | logFC |
| DREB | Os06g06540 | 0.61 | 1.37 | 0.96 | 0.12 | 0.85 | 0.67 |
| | Os08g43200 | 0.97 | -0.34 | 0.92 | 0.20 | 0.81 | -0.61 |
| | Os09g35030 | 0.18 | 2.97 | 0.56 | -0.50 | 0.65 | 0.71 |
| | Os08g43210 | 0.87 | 1.10 | 0.51 | 1.55 | 0.82 | -0.58 |
| | Os09g35010 | 0.13 | 3.83 | 0.79 | -0.31 | 0.33 | -1.72 |
| | Os02g45420 | 0.25 | 3.06 | 0.11 | 2.21 | 0.93 | -0.30 |
| | Os04g46440 | 0.68 | 0.76 | 0.04 | 1.29 | 0.82 | 0.23 |
| | Os01g04020 | 0.52 | 0.90 | 0.97 | 0.02 | 0.64 | 0.44 |
| | Os05g25260 | 0.65 | 0.70 | 0.72 | 0.21 | 0.77 | 0.29 |
| | Os08g45110 | 0.98 | -0.21 | 0.01 | 4.81 | 0.13 | 3.47 |
| | Os03g05590 | 0.77 | -0.58 | 0.51 | -0.55 | 0.84 | 0.58 |
| | Os05g27930 | 0.27 | 1.77 | 0.04 | 1.60 | 0.15 | 1.57 |
| | Os05g37640 | 0.98 | -0.25 | 0.94 | -0.21 | 0.80 | -0.83 |
| | Os02g09650 | 0.41 | -1.12 | 0.02 | 1.49 | 0.58 | 0.49 |
| | Os06g36000 | 0.96 | -0.67 | 0.28 | 1.59 | 0.79 | -0.71 |
| | Os02g54160 | 0.66 | -1.27 | 0.98 | -0.03 | 0.98 | 0.04 |
| | Os06g07030 | 0.96 | 0.23 | 0.01 | 3.52 | 0.04 | 2.92 |
| | Os04g55520 | 0.66 | 0.87 | 0.07 | 1.25 | 0.33 | 1.05 |
| | Os01g07120 | 0.85 | -0.43 | 0.01 | 2.57 | 0.09 | 1.78 |
| | Os02g10760 | 0.74 | -1.10 | 0.03 | -2.75 | 0.92 | 0.20 |
| | Os02g43940 | 0.97 | 0.18 | 0.37 | -0.65 | 0.77 | 0.35 |
| | Os04g46410 | 0.42 | 1.28 | 0.91 | -0.13 | 0.75 | 0.57 |
| | Os10g41130 | 0.64 | -1.42 | 0.84 | 0.39 | 0.96 | -0.17 |
| | Os02g43820 | 0.69 | 2.21 | 0.06 | 5.22 | 0.21 | 4.15 |
| | Os04g46250 | 0.93 | -0.77 | 0.93 | -0.24 | 0.78 | 0.88 |
| | Os06g11940 | 0.65 | -1.23 | 0.01 | -2.93 | 0.35 | -2.06 |
| | Os04g46400 | 0.33 | -1.37 | 0.27 | -0.45 | 0.73 | 0.37 |
| | Os04g34970 | 0.41 | -1.34 | 0.00 | 4.19 | 0.02 | 4.33 |
| | Os02g43970 | 0.76 | 0.64 | 0.02 | 1.69 | 0.12 | 1.66 |
| | Os10g38000 | 0.94 | 0.66 | 0.45 | 1.36 | 0.81 | 0.65 |
| | Os05g32270 | 0.97 | -0.15 | 0.33 | 0.51 | 0.58 | -0.52 |
| | Os04g52090 | 0.33 | 1.66 | 0.02 | 2.08 | 0.21 | 1.46 |
| | Os06g06970 | 0.89 | 0.67 | 0.91 | 0.23 | 0.52 | 1.04 |
| | Os02g52670 | 0.16 | 3.67 | 0.01 | 5.10 | 0.04 | 4.50 |
| | Os06g11860 | 0.98 | -0.11 | 0.41 | 0.50 | 0.95 | 0.09 |
| | Os06g09390 | 0.99 | 0.09 | 0.22 | 0.72 | 0.92 | 0.13 |
| | Os06g09790 | 0.96 | 0.51 | 0.41 | 1.56 | 0.96 | 0.11 |
| | Os08g31580 | 0.73 | 0.63 | 0.00 | 3.29 | 0.05 | 2.31 |
| | Os06g09717 | 0.93 | -0.50 | 0.79 | 0.43 | 0.90 | 0.50 |
| | Os03g09170 | 0.44 | 1.12 | 0.00 | 4.03 | 0.02 | 3.63 |
| | Os06g09810 | 1.00 | 0.00 | 0.57 | -0.33 | 0.50 | -0.58 |
| | Os02g38090 | 0.77 | 0.70 | 0.85 | 0.17 | 0.72 | 0.69 |
| | Os04g36640 | 0.98 | -0.12 | 0.95 | 0.05 | 0.99 | 0.01 |
| | Os02g45450 | 0.19 | 2.20 | 0.21 | -0.84 | 0.12 | -4.04 |
| | Os06g10780 | 0.17 | 3.42 | 0.70 | 0.57 | 0.64 | 1.14 |
| | Os10g22600 | 0.96 | -0.55 | 0.31 | 1.95 | 0.66 | 1.40 |
| | Os08g27220 | 1.00 | 0.02 | 0.16 | 0.82 | 0.92 | -0.22 |
| | Os09g20350 | 0.86 | 0.46 | 0.21 | 0.78 | 0.74 | 0.37 |
| | Os03g08500 | 0.75 | 0.57 | 0.31 | 0.42 | 0.52 | 0.58 |
| | Os05g49010 | 0.99 | 0.04 | 0.02 | -2.68 | 0.08 | -3.22 |
| ERF | Os01g10370 | 0.80 | -1.40 | 0.13 | 1.73 | 0.62 | 0.96 |
| | Os01g21120 | 0.10 | 3.14 | 0.01 | 2.58 | 0.23 | 1.45 |
| | Os10g25170 | 0.70 | 0.79 | 0.95 | 0.04 | 0.37 | 0.88 |
| | Os03g22170 | 0.83 | 0.49 | 0.18 | 0.82 | 0.21 | 1.49 |
| | Os07g10410 | 0.92 | -0.62 | 0.41 | -1.49 | 0.95 | -0.13 |
| | Os01g54890 | 0.38 | -1.43 | 0.01 | 2.48 | 0.02 | 2.93 |

| | | | | | | |
|---|---|---|---|---|---|---|
| Os05g41760 | 0.39 | 1.16 | 0.01 | 2.54 | 0.23 | 1.20 |
| Os01g73770 | 0.24 | 3.98 | 0.05 | 3.91 | 0.07 | 4.76 |
| Os06g42990 | 1.00 | 0.03 | 0.44 | -0.65 | 0.64 | 0.62 |
| Os02g06330 | 0.99 | 0.07 | 0.20 | 0.64 | 0.99 | -0.03 |
| Os04g48350 | 0.34 | 2.00 | 0.27 | -1.10 | 0.13 | -2.73 |
| Os02g29550 | 0.91 | -0.38 | 0.22 | -0.63 | 0.49 | -0.65 |
| Os04g32620 | 0.81 | 0.57 | 0.01 | 2.89 | 0.13 | 2.32 |
| Os07g42510 | 0.91 | 0.33 | 0.33 | 0.44 | 0.71 | 0.41 |
| Os07g47790 | 0.22 | 1.88 | 0.01 | 3.18 | 0.10 | 1.97 |
| Os01g12440 | 0.80 | -0.61 | 0.94 | 0.06 | 0.95 | 0.09 |
| Os03g08490 | 0.77 | 0.57 | 0.73 | 0.17 | 0.14 | 1.55 |
| Os03g15660 | 0.79 | 1.46 | 0.76 | 0.38 | 0.19 | 1.95 |
| Os07g47330 | 1.00 | -0.01 | 0.22 | -2.34 | 0.91 | -0.42 |
| Os07g38750 | 0.89 | -0.84 | 0.57 | -1.29 | 0.86 | -0.58 |
| Os05g28350 | 0.94 | -0.38 | 0.52 | 0.83 | 0.85 | -0.31 |
| Os09g11460 | 0.41 | -1.28 | 0.05 | -1.20 | 0.59 | 0.57 |
| Os09g11480 | 0.34 | -1.89 | 0.01 | -2.78 | 0.78 | 0.31 |
| Os02g54050 | 0.92 | -0.58 | 0.23 | 1.64 | 0.18 | 2.43 |
| Os06g08340 | 0.32 | -1.37 | 0.84 | 0.15 | 0.43 | 0.67 |
| Os03g08460 | 0.78 | 0.55 | 0.20 | -0.90 | 0.67 | -0.42 |
| Os09g26420 | 0.98 | 0.14 | 0.34 | -0.48 | 0.81 | -0.25 |
| Os04g44670 | 0.66 | -0.67 | 0.05 | 1.16 | 0.27 | 1.06 |
| Os02g42585 | 0.89 | 0.40 | 0.98 | 0.03 | 0.96 | -0.06 |
| Os01g46870 | 0.58 | -1.77 | 0.21 | 1.93 | 0.72 | 0.68 |
| Os02g43790 | 0.11 | 2.53 | 0.02 | 1.73 | 0.03 | 2.94 |
| Os03g64260 | 0.49 | 2.38 | 0.21 | 1.82 | 0.04 | 5.34 |
| Os03g07830 | 0.68 | -1.21 | 0.34 | -1.24 | 0.61 | -1.49 |
| Os03g08470 | 0.99 | 0.06 | 0.74 | -0.18 | 0.98 | 0.04 |
| Os03g60120 | 0.86 | 0.49 | 0.16 | 0.86 | 0.37 | 0.94 |
| Os07g12510 | 0.58 | 0.93 | 0.01 | 2.15 | 0.20 | 1.31 |
| Os07g22730 | 0.56 | 1.11 | 0.08 | 1.36 | 0.07 | 2.47 |
| Os05g36100 | 0.41 | -2.41 | 0.84 | 0.16 | 0.47 | 0.92 |
| Os09g39850 | 0.80 | 0.66 | 0.32 | 1.66 | 0.26 | 1.05 |
| Os08g44960 | 0.58 | 2.54 | 0.78 | 0.52 | 0.20 | 3.29 |
| Os09g39810 | 0.98 | 0.21 | 0.36 | 0.62 | 0.90 | -0.14 |
| Os05g41780 | 0.44 | 1.20 | 0.07 | 1.21 | 0.84 | 0.24 |
| Os06g03670 | 0.11 | 3.21 | 0.01 | 4.34 | 0.19 | 1.90 |
| Os06g40150 | 0.97 | 0.14 | 0.18 | -0.62 | 0.65 | -0.42 |
| Os01g58420 | 0.17 | 2.18 | 0.01 | 3.16 | 0.18 | 1.53 |
| Os05g34730 | 0.45 | 1.82 | 0.03 | 3.44 | 0.02 | 5.77 |
| Os04g18650 | 0.94 | 0.71 | 0.44 | 1.46 | 0.79 | 0.83 |
| Os11g06770 | 0.61 | -0.83 | 0.00 | 3.89 | 0.02 | 3.75 |
| Os08g41030 | 0.79 | 0.69 | 0.02 | 2.65 | 0.31 | 1.42 |
| Os02g32140 | 0.60 | -0.95 | 0.00 | 4.07 | 0.05 | 2.74 |
| Os02g34260 | 0.88 | 0.62 | 0.01 | 2.74 | 0.44 | 1.53 |
| Os04g32790 | 0.97 | 0.19 | 0.01 | 2.67 | 0.92 | -0.15 |
| Os02g51670 | 0.98 | -0.11 | 0.41 | 0.50 | 0.95 | 0.09 |
| Os09g28440 | 0.15 | 4.11 | 0.12 | 2.10 | 0.20 | 2.35 |
| Os08g35240 | 0.13 | 3.95 | 0.29 | 1.20 | 0.04 | 3.66 |
| Os04g46220 | 0.78 | 0.61 | 0.20 | 0.84 | 0.16 | 1.63 |
| Os04g46240 | 0.95 | 0.26 | 0.45 | -1.03 | 0.88 | -0.23 |
| Os10g41330 | 0.28 | 1.78 | 0.86 | 0.14 | 0.93 | 0.15 |
| Os05g39590 | 0.94 | 0.95 | 0.99 | -0.06 | 0.51 | 2.28 |
| Os07g03250 | 0.82 | -1.03 | 0.92 | 0.07 | 0.63 | -0.52 |
| Os07g22770 | 0.93 | -0.37 | 0.02 | -2.21 | 0.93 | 0.12 |
| Os12g39330 | 0.71 | 1.40 | 0.89 | -0.37 | 0.33 | 1.60 |
| Os08g36920 | 0.17 | 2.58 | 0.23 | 0.96 | 0.07 | 2.68 |
| Os09g13940 | 0.84 | 0.49 | 0.66 | -0.31 | 0.57 | 0.57 |
| Os08g07700 | 0.98 | -0.30 | 0.59 | 0.76 | 0.99 | 0.03 |
| Os12g41060 | 0.98 | 0.13 | 0.27 | -0.73 | 0.79 | -0.29 |
| Os05g29810 | 0.63 | -0.73 | 0.00 | 3.70 | 0.05 | 2.24 |

| | | | | | | |
|---|---|---|---|---|---|---|
| Os06g47590 | 0.96 | -0.21 | 0.66 | 0.23 | 0.57 | 0.51 |
| Os10g26590 | 0.81 | 1.40 | 0.73 | -0.85 | 0.72 | 0.89 |
| Os02g13710 | 0.77 | -0.71 | 0.23 | -0.85 | 0.95 | 0.12 |

Footnote: 1. LogFC of expression profiles of OsDREB/ERF genes from Gene Expression Omnibus (dataset ID: GSE6901 ) under cold, drought and salt stresses.
2. If the q value was less than 0.05, it was marked in pink.
3. If the LogFC treatment/control at each time point under the corresponding treatment was less than -1, it was marked in green backgroud.
4. If the log2 based fold change of treatment/control at each time point under the corresponding treatment was greater than 1, it was marked in orange backgroud.

Supplementary Table 9:

A: Primers for gene amplification and pGBKT7 vector construction

| sub-family | Gene ID | Forward primer (5'→3') | Reverse primer (5'→3') |
|---|---|---|---|
| DREB | Bradi3g57360 | CG GAATTC ATGGACGGCTCCGGTCAATGG | CG GGATCC CTATAGCCAAAGACTGCAGA |
| | Bradi4g35610 | CG GAATTC ATGGACGGCTCCGATCAACGG | CG GGATCC TCAGTAACTCCACAGGGAGGCG |
| | Bradi4g35570 | CG GAATTC ATGGACGCGGCCGACTCCCC | CG GGATCC TCAGTAGTTCCAGAGCGTGGCC |
| | Bradi4g35580 | CG GAATTC ATGGACGCGGCCAGCTCC | CG GGATCC TCAAAGCAGGATAGAGTGTT |
| | Bradi4g35600 | CG GAATTC ATGGACGGCTACGATCAATGG | CG GGATCC TCAGTAATTCCAGAGGGTGGC |
| | Bradi3g51630 | CGGAATTCATGGACCTCGGTGCTCTCAGC | CG GGATCC TCAGTAGCTCCATAGGTTGAC |
| | Bradi1g49570 | CG GAATTC ATGGATCATCACCACTACGAAG | CG GGATCC TCAGTAGCTCCAGAGTGCGACG |
| | Bradi1g49560 | CG GAATTC ATGGATCATAACCACCACTA | CG GGATCC TCAGTAGCTCCAGAGCGTGA |
| | Bradi5g17640 | CG GAATTC ATGGACGATACTCCCCACGAGC | CG GGATCC TCAGTACTCCCACGGCAGCGGC |
| | Bradi3g50630 | CG GAATTC ATGGGCGCCATGGAGGATTG | CG GGATCC CTAGTACTCCCACATCAGTG |

B: Primers for RT-PCR to investigate the expression profiles of some BdDREB genes in different tissues

| sub-group | Gene ID | Forward primer (5'→3') | Reverse primer (5'→3') |
|---|---|---|---|
| A1 | Bradi2g60340 | TCCTGCTGGGAAAGCGGAGACT | TCATCAGACGTGAACCGACGAG |
| A1 | Bradi2g60331 | GTGCCACTGCCAGTACCACC | TCAGGTGGCTTTAACGGGAT |
| A1 | Bradi4g35580 | TGGCACTACGGAACGAAACACT | TACTCGCCCTCCTGACCATACA |
| A1 | Bradi4g35600 | GTGCTGGGAAATCGGAGAAT | CAGGGAGGCACTGCGAGACA |
| A1 | Bradi1g57970 | CGATGCTGTTTTCTCGTTGG | CAACTCCAGAGGTCAGCATCA |
| A1 | Bradi1g77120 | CTCCTGCAACAAGAAGCGTG | CGGCACCTCAAAATCAAACA |
| A1 | Bradi4g35650 | ATTCGGCACTGTACGGTCAC | CTGCTTTCGTGCTTTGTGGT |
| A1 | Bradi4g35630 | CAGGCTCTGGCTCGGGACGTA | GAACAAGTCAAGCCTGCTGAACAT |
| A1 | Bradi1g49570 | CATCCGCCTCGCCGAGTCCG | GAGCGAGCCCTGCTGCCACT |
| A1 | Bradi1g49560 | GCGGTGGGTGTGCGAGGTCA | GCCAGTCTCCGCCAATGTCGCCA |
| A1 | Bradi5g17640 | ATCAGGGAGCCACGGAAGAAGT | CGAGAGCCGACGAAGACTCATATC |
| A1 | Bradi3g50630 | CCCGCAAGAAGTCCCGCATCTGGCT | CGCAGGAGAGCTGGCAGGACGGA |
| A4 | Bradi3g51610 | CAGGAACGGCAAGTGGGTCT | ACTCCTCCCCCTCATTGCCA |
| A4 | Bradi5g17620 | GCTCTTCCTCCAGCAGCACTCT | GGGCAGCTCGTGCGCGAGGG |
| A4 | Bradi3g33355 | CCTTCCCCACCGCCGACATG | CCACATGGCGGACGAGAACC |
| A4 | Bradi3g45997 | CACGACCATTACTATCAACCCG | TCCCCGCCGCTGCCATCCTG |
| A5 | Bradi2g25050 | CGCCTGTGGCTTGGCTCCTA | GCTGCCCCTTGCTCTCTTCCA |
| A5 | Bradi1g67350 | TGTGGAGCTTCTGCTAGTGTTAGG | TCCAATGTTCTCGGTTCCACTC |
| A5 | Bradi1g46120 | CGCCCACGAACTGGACTACT | CATGGCGTACTCCATGCACTT |
| A5 | Bradi5g24110 | GCAGGTGACCGTGGCTGTGGC | AGTTGAGCCTTGCCGACCTG |
| A2 | Bradi1g72990 | CGAGCCCAACAAGCGCACCC | GACGGAGGCATAGCGTTGGTG |
| A2 | Bradi3g12680 | CCAACAACCCCGTCCTCGCTCC | GTCGCAGGAGGGTTCCCAGAT |
| A2 | Bradi2g04000 | CCAAGGGTTCCAAGAAAGGG | GATGGATGTGACGCAGTGGTT |
| A2 | Bradi2g29960 | CGTCTATTCTGCGGAAAAAGCG | CATAAGCACAGGCAGCAGTTTC |
| A6 | Bradi3g35560 | CTCAGACCGGGCTGGCCTCT | GCGTTGATGGGCGTGGCCTT |
| A6 | Bradi4g29010 | CGCCATTCTCCTTCCCCCACGC | TGCCGAGCCAGAGGCGGGTGCG |
| A6 | Bradi3g58980 | GTTCCGACAACCACCGCCCTT | AAGGTGCCGAGCCACAGCCGC |
| A6 | Bradi1g45470 | ATCCCCATTATTATTCCCTTT | GCCGACAGGGACAAACAACTC |
| | β-actin | CCCGATGGACAGGTTATCACTA | ATAGAGCCACCAATCCAAACAC |
